# Conjugates of amiridine and salicylic derivatives as promising multifunctional CNS agents for potential treatment of Alzheimer's disease

**DOI:** 10.1002/ardp.202400819

**Published:** 2024-12-17

**Authors:** Galina F. Makhaeva, Maria V. Grishchenko, Nadezhda V. Kovaleva, Natalia P. Boltneva, Elena V. Rudakova, Tatiana Y. Astakhova, Elena N. Timokhina, Pavel G. Pronkin, Sofya V. Lushchekina, Olga G. Khudina, Ekaterina F. Zhilina, Evgeny V. Shchegolkov, Maria A. Lapshina, Elena S. Dubrovskaya, Eugene V. Radchenko, Vladimir A. Palyulin, Yanina V. Burgart, Victor I. Saloutin, Valery N. Charushin, Rudy J. Richardson

**Affiliations:** ^1^ Institute of Physiologically Active Compounds at Federal Research Center of Problems of Chemical Physics and Medicinal Chemistry Russian Academy of Sciences Chernogolovka Russia; ^2^ Postovsky Institute of Organic Synthesis Urals Branch of the Russian Academy of Sciences Ekaterinburg Russia; ^3^ Emanuel Institute of Biochemical Physics Russian Academy of Sciences Moscow Russia; ^4^ Department of Biomolecular Sciences Weizmann Institute of Science Rehovot Israel; ^5^ Department of Chemistry Lomonosov Moscow State University Moscow Russia; ^6^ Department of Environmental Health Sciences University of Michigan Ann Arbor Michigan USA; ^7^ Department of Neurology University of Michigan Ann Arbor Michigan USA; ^8^ Center for Computational Medicine and Bioinformatics University of Michigan Ann Arbor Michigan USA; ^9^ Michigan Institute for Computational Discovery and Engineering University of Michigan Ann Arbor Michigan USA

**Keywords:** Alzheimer's disease, amiridine, multifunctional activity, salicylic derivatives

## Abstract

New conjugates of amiridine and salicylic derivatives (salicylamide, salicylimine, and salicylamine) with different lengths of alkylene spacers were designed, synthesized, and evaluated as potential multifunctional central nervous system therapeutic agents for Alzheimer's disease (AD). Conjugates demonstrated high acetylcholinesterase (AChE) and butyrylcholinesterase (BChE) inhibition (IC_50_: AChE, 0.265−4.24 μM; BChE, 0.01−0.64 μM) but poor activity against off‐target carboxylesterase (CES). Specifically, conjugates with a (CH_2_)_8_ spacer showed the highest AChE and BChE inhibition: 3–16 times more effective than amiridine. Salicylamides **7b** and **7c** had the maximum BChE/AChE selectivity ratios: 193 and 138, respectively. Conjugates were mixed‐type reversible inhibitors of both cholinesterases and displaced propidium from the AChE peripheral anionic site (PAS) at the level of donepezil. All conjugates inhibited Aβ_42_ self‐aggregation in the thioflavin test; inhibition increased with spacer elongation, being greatest for (CH_2_)_8_. The results agreed with molecular docking to AChE, BChE, and Aβ_42_. Conjugates exhibited high 2,2′‐azino‐bis(3‐ethylbenzothiazoline‐6‐sulfonic acid) (ABTS)^•+^‐scavenging activity comparable to the standard antioxidant Trolox, and they showed the ability to bind Cu^2+^, Fe^2+^, and Zn^2+^. Conjugates had favorable predicted intestinal absorption and blood–brain barrier permeability. Altogether, the results indicate that the new conjugates possess potential for further development as multifunctional anti‐AD drug candidates.

## Graphical abstract

Novel conjugates of amiridine and salicylic derivatives linked with alkylene spacers were synthesized as potential multifunctional central nervous system (CNS) agents for Alzheimer's disease treatment. The conjugates exhibited high anti‐acetylcholinesterase (AChE) and anti‐butyrylcholinesterase (BChE) activities, inhibited Aβ_42_ self‐aggregation (up to 96.7%), showed high ABTS^•+^‐scavenging activity and the ability to bind Cu^2+^, Fe^2+^, Zn^2+^, and had favorable predicted blood‐brain barrier permeability.

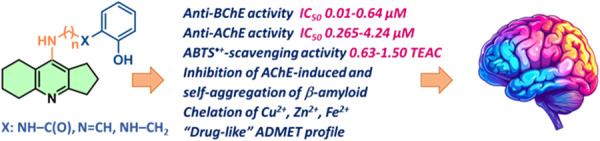

## 1 INTRODUCTION

Dementia is often called the epidemic of the 21st century, as there are currently 52 million people with dementia in the world, and almost 8 million new cases are diagnosed each year. The World Health Organization estimates that by 2050, the number of people with dementia will approach 150 million. This global medical and social problem presents colossal challenges to everyone. In particular, chemists, pharmacologists, toxicologists, and physicians face difficult technical challenges as they strive to discover and develop effective and safe therapeutic agents for dementia, including its most common form, Alzheimer's disease (AD).^[^ ^1^, ^2^ ^]^

Today, the consensus view of AD is that its etiology is multifactorial. The pathogenetic factors of the disease include disruption of neurotransmitter pathways, aggregation of β‐amyloid and tau proteins, imbalance of redox systems, and dysregulation of the homeostasis of biometal ions.^[^ ^3^ ^]^ In this regard, a creative strategy for AD therapeutics is the development of multitarget‐directed ligands (MTDLs), which can interact synergistically with two or more pathogenic components.^[^ ^1^, ^2^, ^4^, ^5^, ^6^, ^7^ ^]^

Our team actively uses a logical approach to craft MTDLs consisting of selecting two pharmacophores, each of which is known to suppress the activity of at least one of the causative agents of AD.^[^ ^6^, ^8^, ^9^ ^]^ Thus, for AD treatment, the use of an anticholinesterase compound as one of the pharmacophores is a commonly employed approach.^[^ ^1^, ^9^, ^10^, ^11^, ^12^ ^]^ The range of pharmacological activities of a parent anticholinesterase compound can be expanded by adding properties that can result in neuroprotection or disease modification, such as the prevention of protein aggregation or oxidative stress.^[^ ^9^, ^11^, ^12^, ^13^, ^14^, ^15^, ^16^, ^17^ ^]^

**Figure 1:**
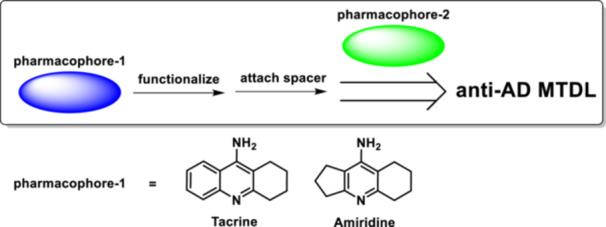
Strategy for the synthesis of multitarget‐directed ligands (anti‐AD MTDLs) and the structures of anticholinesterase pharmacophores tacrine and amiridine.

In addition to the widely used anticholinesterase compound tacrine,^[^ ^12^, ^16^, ^17^ ^]^ we have extensively applied the amiridine molecule (Figure 1) as an anticholinesterase pharmacophore. Amiridine (also known as 9‐amino‐2,3,5,6,7,8‐hexahydro‐1*H*‐cyclopenta[*b*]quinoline monohydrochloride monohydrate, axamon, ipidacrine, impgrix, neuramidin, or NIH‐247) is an anticholinesterase agent that has been employed in Eastern European countries for a variety of neurodegenerative diseases including AD.^[^ ^18^, ^19^, ^20^, ^21^, ^22^, ^23^, ^24^, ^25^ ^]^

We have obtained several classes of amiridine conjugates with linkers containing N‐acyl‐ or N‐thiourea moieties. The strategy for their synthesis involved the functionalization of the amiridine external amino group using highly reactive chlorine‐containing agents such as chloroacetic acids chloride,^[^ ^26^ ^]^ chlorocarboxylic acids chloride,^[^ ^27^ ^]^ and thiophosgene^[^ ^28^ ^]^ for introducing a spacer followed by conjugation with a second pharmacophore.

Several groups of amiridine conjugates with an N‐acyl‐containing spacer were synthesized. These included conjugates with the following second pharmacophores: N‐substituted piperazines,^[^ ^26^ ^]^ thiouracils^[^ ^27^ ^]^; memantine/adamantylamine, trolox, and substituted benzothiazoles^[^ ^29^ ^]^; and oxa/azaheterocycles.^[^ ^30^ ^]^ In addition, we have prepared conjugates with (het)arylalkylamines using a thiourea spacer,^[^ ^28^ ^]^ as well as bis‐amiridines with bis‐*N*‐acyl or bis‐*N*‐thiourea‐alkylene spacers.^[^ ^31^ ^]^ Along with inhibition of cholinesterases, the synthesized conjugates demonstrated antioxidant properties in addition to their ability to inhibit AChE‐induced spontaneous and AChE‐induced aggregation of β‐amyloid, thereby showing themselves to be multifunctional agents with potential capacities for disease modification or neuroprotection. The spectrum of pharmacological activity of the hybrid compounds was provided by both the second pharmacophore and the structure of the spacer. However, a characteristic feature of the described groups of conjugates was their low inhibitory activity against cholinesterases compared to amiridine itself. We hypothesized that the lack of high anticholinesterase potency could have resulted from the methods previously used to functionalize the amiridine molecule and to attach a suitable spacer for conjugation with favorable second pharmacophores. Therefore, an important objective of the present work was to synthesize amiridine‐based conjugates using an aminoalkylene spacer.

Salicylic derivatives, which are promising scaffolds in the design of anti‐AD MTDLs, were used as the second pharmacophore. It is known that this moiety possesses an extensive array of desirable bioactive attributes, encompassing abilities to act as antioxidants, metal chelators, and inhibitors of β‐amyloid aggregation.^[^ ^32^, ^33^, ^34^ ^]^ Earlier, we synthesized conjugates consisting of a set of salicylic acid derivatives linked to tacrine with an alkylene spacer. These hybrids proved to be effective as cholinesterase inhibitors, antioxidants, chelating agents, and inhibitors of spontaneous and AChE‐induced aggregation of β‐amyloid.^[^ ^16^, ^35^ ^]^

In the present work, we developed a novel approach for obtaining hybrids of amiridine based on its chloro derivative that allowed us to produce a new type of amiridine conjugates, whereby we could couple the amiridine molecule with a second pharmacophore via alkylene spacers of various lengths. We used salicylic derivatives (salicylamide, salicylimine, and salicylamine) as pharmacophore 2 and varied the length of the linker from four to eight methylene groups. We then assessed the synthesized conjugates as potential anti‐AD multifunctional agents by carrying out a series of biological characterizations as follows: (1) Inhibition of the cholinergic esterases (AChE and BChE) and the homologous off‐target carboxylesterase (CES); (2) Mechanism of antiesterase activity using enzyme kinetics and molecular docking assisted by quantum mechanics (QM); (3) Displacement of propidium from the AChE peripheral anionic site (PAS) as an indicator of the potential to inhibit AChE‐assisted aggregation of Aβ (1‐42); (4) Inhibition of spontaneous aggregation of Aβ_42_ via the thioflavin test and prediction of binding modes to Aβ_42_ using molecular docking; (5) Antioxidant capability by the ABTS and FRAP tests; (6) QM analysis of antioxidant structure–activity relationships; (7) Chelation of Cu^2+^, Zn^2+^, Fe^2+^ by UV‐Vis spectroscopy; (8) Cytotoxicity of the conjugates against three cell lines by the 3‐(4,5‐dimethylthiazol‐2‐yl)−2,5‐diphenyltetrazolium bromide (MTT) assay; and (9) Computational prediction of absorption, distribution, metabolism, elimination, and toxicity (ADMET) characteristics.

## 2 RESULTS AND DISCUSSION

### 2.1 Chemistry

**Scheme 1:**
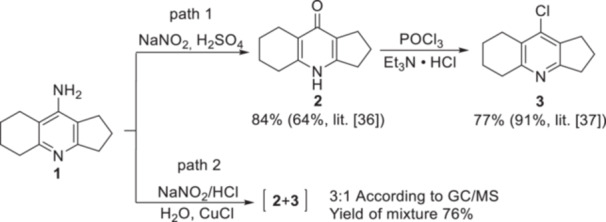
Synthesis of 9‐chloro‐2,3,5,6,7,8‐hexahydro‐1*H*‐cyclopenta[*b*]quinolone **3**.

The synthesis of target conjugates was carried out starting from the Cl‐derivative of amiridine, the preparation of which is described in previous publications.^[^ ^36^, ^37^ ^]^ At the first stage, amiridine **1** was diazotized with sodium nitrite in 10% (v/v) H_2_SO_4_ to isolate the 4‐pyridone derivative **2** from the reaction mixture. Then compound **2** was heated in POCl_3_ in the presence of Et_3_N.HCl to obtain 9‐chloro‐2,3,5,6,7,8‐hexahydro‐1*H*‐cyclopenta[*b*]quinoline **3** with an overall yield of 64% (Scheme 1, path 1).

We previously attempted to carry out a one‐pot synthesis of the Cl‐derivative of amiridine **3** using the Sandmeyer reaction by diazotization/chlorination in the presence of CuCl. According to gas chromatography‐mass spectrometry data, this reaction proceeded nonselectively. The predominant product in this case was pyridone **2** (58% yield), and the yield of the target compound **3** was only 19% (Scheme 1, path 2). Thus, the synthesis of the Cl‐derivative of amiridine **3**, described in Zhidkova et al.,^[^ ^37^ ^]^ proceeds selectively, with a higher yield and turns out to be preferable.

The next stage involved the development of a method for introducing an aminoalkylene spacer into the amiridine molecule. The only example of the chlorine atom substitution in the Cl‐amiridine **3** with an amino group was described for the reaction with methylamine.^[^ ^37^ ^]^ The methylamine group was introduced under harsh conditions by heating the compound **3** in a 25% (v/v) aqueous solution of methylamine at 210°C in an autoclave for 18 h with a yield of 97%.

Initially, we tried to substitute the chlorine atom in 9‐chloro‐2,3,5,6,7,8‐hexahydro‐1*H*‐cyclopenta[*b*]quinoline **3** with the amino group of various diaminoalkanes in pentanol‐1 at 160°C or phenol at 180°C in the presence of KI or NaI similarly to an aminoalkylene spacer introduction into the structural analog tacrine.^[^ ^38^, ^39^ ^]^ However, in pentanol‐1 at 160°C the substitution of the chlorine atom in compound **3** with the amino group of 1,4‐diaminobutane **4a**, 1,6‐diaminohexane **4b** and 1,8‐diaminooctane **4c** did not proceed, which was apparently due to the lower reactivity of the chlorine atom in derivative **3** compared to the tacrine analog.

**Scheme 2:**
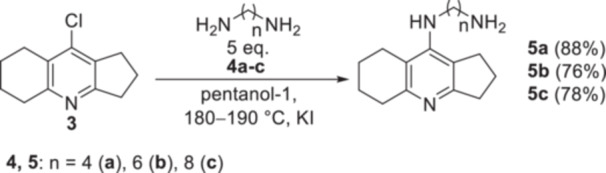
Introduction of an aminoalkylene spacer into Cl‐amiridine.

By raising the reaction temperature to 180–190°C, increasing the time to 16 h, and placing the mixture in a closed stainless‐steel vessel, the reaction of Cl‐amiridine **3** with diamines **4a–c** in pentanol‐1 was allowed to proceed. Although complete conversion of the starting compound **3** occurred under these conditions, it was accompanied by partial resinification of the mixture that decreased the yield of the target aminoalkylene derivatives **5a–c** to 55%–61%. Carrying out the reaction in an argon atmosphere increased the yield to 76%–88%, due to the prevention of oxidative processes leading to resinification (Scheme 2). In addition, phenol was tested as a solvent for the synthesis of the aminooctane derivative **5c**. The reaction proceeded efficiently, although with a slightly lower yield (70%) than in pentanol‐1 (78%) (Table 1).

**Table 1:** The yield of amine **5c** depending on the reaction conditions.

Solvent	Temperature,°C	Atmosphere	Yield, %
Pentanol‐1	160	Air	not proceed
Pentanol‐1	180–190	Air	55
Pentanol‐1	180–190	Argon	78
Phenol	180–190	Argon	70

**Scheme 3:**
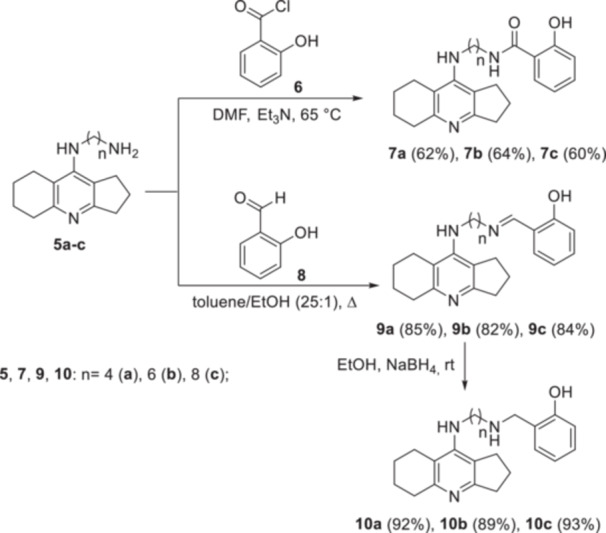
Synthesis of conjugates **7а–с**, **9а–с** and **10а–с**.

The target conjugates were obtained by introducing fragments of salicylic derivatives into the amines **5a–c**. Thus, the acylation of compounds **5a–c** with salicylic acid chloride **6** in DMF in the presence of Et_3_N at 65°C led to the formation of amiridine conjugates with salicylamide **7a–c**. The condensation of derivatives **5a–c** with salicylic aldehyde **8** in a boiling toluene/ethanol mixture (25:1 by volume) with azeotropic distillation of water resulted in the conjugates of amiridine with salicylimine **9a–c**. The reduction of imines **9a–c** with sodium borohydride in EtOH allowed the conjugates of amiridine with salicylamine **10a–c** to be obtained (Scheme 3).

Structures of conjugates **7**, **9**, **10** were confirmed by IR and ^1^H, ^13^C nuclear magnetic resonance (NMR) spectroscopy, elemental analysis, mass spectrometry, and X‐ray diffraction (XRD) analysis.

The ^1^H NMR spectra of the amides **7a–c** showed a broadened singlet signal of the amide proton at δ 6.39–6.79 ppm, and the carbon atom signal of the amide fragment was observed at δ ~169 ppm in the ^13^C NMR spectra. The signals corresponding to the proton and carbon atom of the imine group of compounds **9a–c** were characteristic at δ ~8.3 ppm in the ^1^H NMR spectra and at ~164.46–164.99 in the ^13^C NMR spectra. The singlet signal at δ ~4.0 ppm corresponding to the protons of the aminomethylene moiety was observed in the ^1^H NMR spectra of amines **10a–c**.

The IR spectra also changed depending on the structures of the conjugates. In the IR spectra of amides **7a–c**, an absorption band of the carbonyl group was observed at *ν* 1640–1647 cm^−1^, and for imines **9a–c** the characteristic band was at *ν* 1631–1632 cm^−1^, corresponding to the vibrations of the C═N group. In the IR spectrum of amines **10a–c**, no absorption band was observed in the region *ν* 1600–1700 cm^−1^, but there was a band at ν 1573–1576 cm^−1^, attributed to the secondary amino group vibrations.

**Figure 2:**
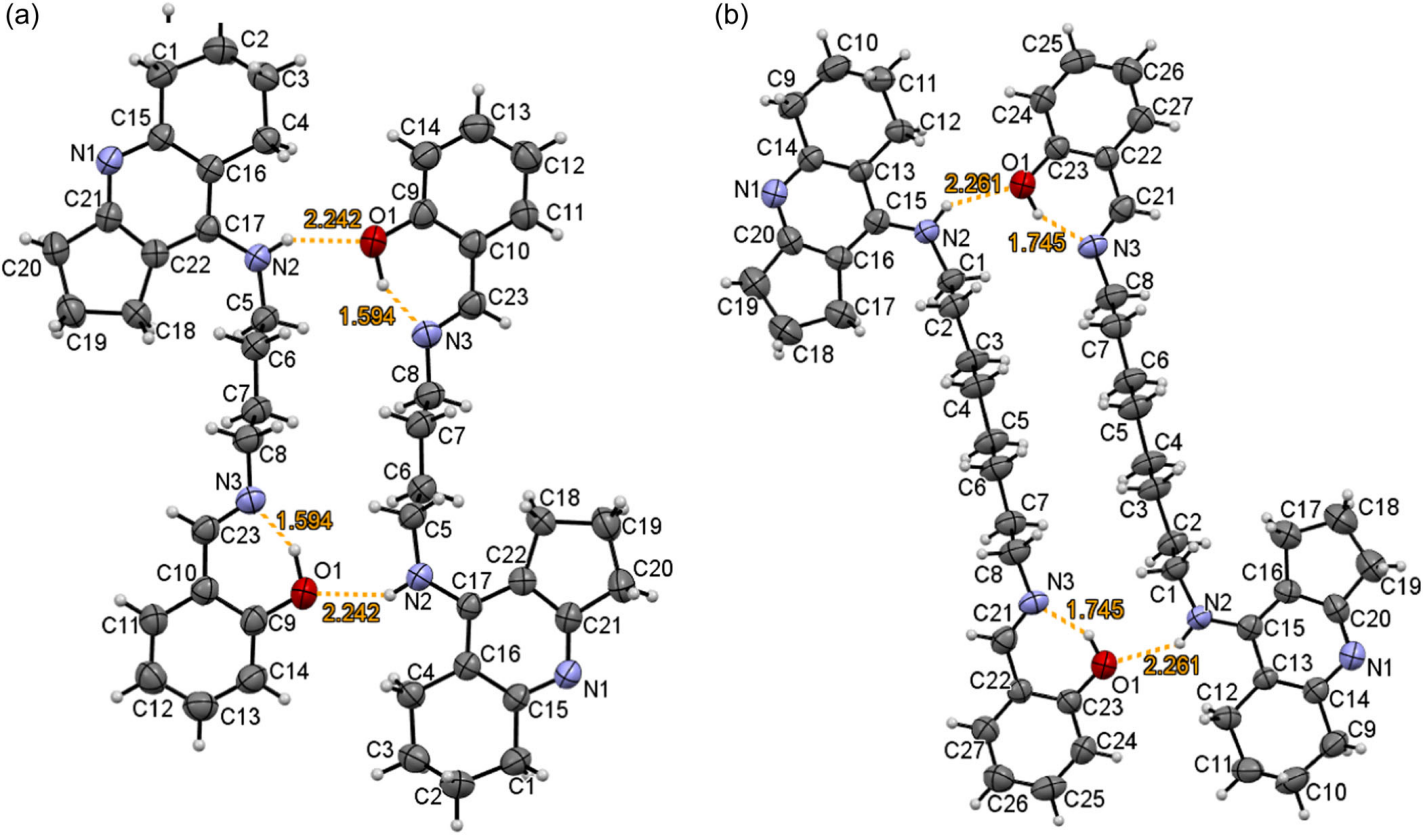
Oak Ridge Thermal‐Ellipsoid Plot Program views of conjugates **9a** (a) and **9с** (b).

Single crystals of imines **9a,c** were obtained by slow crystallization from a dimethylsulfoxide (DMSO)/CHCl_3_ mixture (1:100, v:v). According to the XRD analysis, the conjugates **9a,c** exist in the solid state in the form of dimeric structures of the “head‐tail” type, in which intermolecular hydrogen bonds between the O1 oxygen atoms of the phenolic fragment and the H2 proton of the exocyclic amino group of amiridine (for **9a**: the distance O1···H2 2.242 Å; for **9с**: the distance О1···Н2 2.261 Å) are realized. The intramolecular hydrogen bond between the H1 proton and the N3 nitrogen atom of the imine fragment also contributes to the stabilization of the molecule (for **9a**: the distance H1···N3 is 1.594 Å; for **9c**: the distance H1···N3 is 1.745 Å) (Figure 2).

**Scheme 4:**
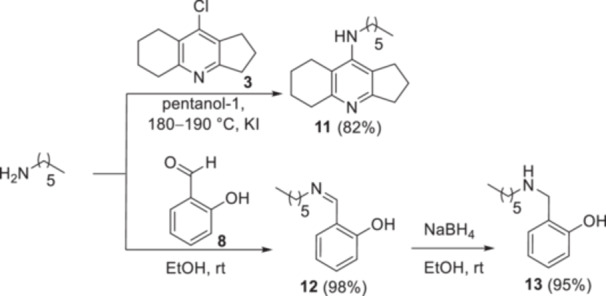
The synthesis of *N*‐hexyl‐2,3,5,6,7,8‐hexahydro‐1*H*‐cyclopenta[*b*]quinolin‐9‐amine **11**, *N*‐hexylsalicylimine **12**, and *N*‐hexylsalicylamine **13**.

In addition, *N*‐hexyl‐2,3,5,6,7,8‐hexahydro‐1*H*‐cyclopenta[*b*]quinolin‐9‐amine **11**, *N*‐hexylsalicylimine **12**, and *N*‐hexylsalicylamine **13** were obtained as model compounds. One more model compound, *N*‐hexylsalicylamide **14,** was prepared and studied in our previous work.^[^ ^16^ ^]^ Substitution of the chlorine atom in compound **3** with *N*‐hexylamine under the reaction conditions with diamines **4** allowed us to obtain the *N*‐hexyl derivative of amiridine **11**. Imine **12** and amine **13** were synthesized from salicylic aldehyde **8** by its condensation with *N*‐hexylamine followed by reduction with NaBH_4_ (Scheme 4).

### 2.2 Biology

#### 2.2.1 Inhibition studies of AChE, BChE, and CES

The esterase profile of the synthesized conjugates was determined. This included an assessment of their inhibition of cholinergic targets AChE and BChE along with a structurally related enzyme CES (EC 3.1.1.1), which hydrolyzes numerous ester‐containing drugs and is considered as an off‐target for anti‐AD agents.^[^ ^9^, ^40^ ^]^ Human erythrocyte AChE, equine serum BChE, and porcine liver CES were used.^[^ ^40^, ^41^, ^42^ ^]^ Amiridine and *N*‐hexyl‐amiridine **11** were used as reference compounds. Bis‐4‐nitrophenyl phosphate (BNPP), a selective CES inhibitor, was used as a positive control. The results are shown in Table 2.

**Table 2:** Esterase profiles of the compounds, their ability to displace propidium from the PAS of *Electrophorus electricus* AChE (*Ee*AChE), and their inhibition of Aβ_42_ self‐aggregation.

No	Compound 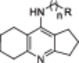		Inhibitory activity against AChE, BChE and CES (%)^a^ or IC_50_ (µM)^b^			Selectivity index IC_50_AChE/IC_50_BChE	Propidium displacement, %^c^	Inhibition of Aβ_42_ self‐aggregation, %^d^
	R	n	AChE	BChE	CES			
Salicylamides								
**7a**	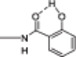	4	1.52 ± 0.14 µM	0.254 ± 0.023 µM	5.0 ± 0.1	6.0	10.8 ± 0.8	41.3 ± 3.3
**7b**		6	4.24 ± 0.08 µM	0.022 ± 0.001 µM	12.3 ± 1.2	193	11.6 ± 0.9	53.4 ± 4.2
**7с**		8	1.53 ± 0.03 µM	0.0111 ± 0.0001 µM	32.9 ± 0.9	138	11.8 ± 0.8	74.1 ± 5.1
Salicylimines								
**9a**	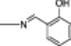	4	3.31 ± 0.02 µM	0.642 ± 0.007 µM	10.5 ± 0.9	5.2	12.5 ± 1.1	20.1 ± 1.4
**9b**		6	3.25 ± 0.08 µM	0.062 ± 0.006 µM	12.4 ± 1.2	52.4	11.7 ± 0.8	59.1 ± 4.7
**9с**		8	1.42 ± 0.14 µM	0.025 ± 0.001 µM	25.8 ± 0.7	56.8	11.6 ± 0.9	71.7 ± 5.1
Salicylamines								
**10a**	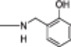	4	2.40 ± 0.15 µM	0.198 ± 0.019 µM	6.2 ± 0.5	12.1	13.6 ± 0.9	30.5 ± 2.4
**10b**		6	1.19 ± 0.05 µM	0.0294 ± 0.0006 µM	18.6 ± 0.1	40.5	15.1 ± 1.2	79.2 ± 5.5
**10с**		8	0.265 ± 0.018 µM	0.0220 ± 0.0002 µM	10.5 ± 0.1 µM	12.0	15.8 ± 1.1	96.7 ± 6.7
Amiridine			4.44 ± 0.36 µM	0.272 ± 0.015 µM	2.7 ± 0.5	16.3	12.2 ± 0.9	6.4 ± 0.5
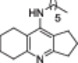 **11**			4.30 ± 0.32 µM	0.262 ± 0.008 µM	5.6 ± 0.6	16.4	n.d.	n.d.
Salicylic acid			n.a.	47.6 ± 3.2 µM	8.9 ± 0.6	‐	n.d.	n.d.
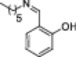 **12**			n.а.	10.6 ± 0.1	1.9 ± 0.1	‐	n.d.	n.d.
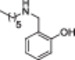 **13**			14.0 ± 0.6	26.9 ± 0.5	14.8 ± 0.5	‐	n.d.	n.d.
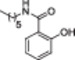 **14** ^[^ ^35^ ^]^			n.a.	10.6 ± 0.8	4.9 ± 1.0	‐	n.d.	n.d.
BNPP			n.а.	n.а.	1.80 ± 0.11 µM	‐	n.а.	n.а.
Myricetin			n.d.	n.d.	n.d.	‐	n.d.	79.4 ± 6.3
Donepezil			0.0400 ± 0.0037 µM	19.2 ± 3.0 µM	n.а.	0.002	11.9 ± 0.9	n.d.
Propidium iodide			n.d.	n.d.	n.d.	‐	n.d.	90.7 ± 7.1

*Note*: All values are presented as Mean ± SEM, *n* = 3.

Abbreviations: AChE, acetylcholinesterase; BChE, butyrylcholinesterase; CES, carboxylesterase; n.a., not active; n.d., not determined.

^a^ Values without units of measurement for AChE, BChE, and CES inhibition correspond to % inhibition at 20 μM.

^b^ For active compounds (inhibition at 20 μM ≥ 30%), AChE and BChE inhibition is presented as IC_50_ ± SEM μM, *n* = 3.

^c^ Propidium displacement from the *Ee*AChE PAS at 20 µM compound concentration.

^d^ Inhibition of Aβ_42_ (50 µM) self‐aggregation by the tested compound at 100 µM concentration.

The study of the esterase profile of conjugates **7**, **9**, **10** showed (Table 2) that all of them effectively inhibited AChE and BChE either at the same level or much higher than the original pharmacophore amiridine. Furthermore, like amiridine, the compounds were more selective toward BChE. Salicylamide conjugates **7b** and **7c** with IC_50_ 22 ± 1 and 11 ± 0.1 nM, respectively, had maximum selectivity index values of 193 and 138, respectively. All compounds rather weakly inhibited the off‐target CES. More noticeable anti‐CES activity appeared in conjugates with a (CH_2_)_8_ spacer.

For all groups of conjugates, increasing the spacer length from (CH_2_)_4_ to (CH_2_)_8_ led to a substantial increase in activity against BChE (9–26 times depending on the group of conjugates). The maximum activity against BChE was exhibited by the salicylamide derivative **7c** with spacer (CH_2_)_8_. This compound inhibited BChE with IC_50_ = 11.1 ± 0.1 nM, which is 25 times more effective than amiridine and close to the inhibitor activity of its tacrine analog (IC_50_ = 10.4 ± 1.3 nM).^[^ ^16^ ^]^

As for AChE inhibition, the most noticeable effect of the spacer length was observed in the group of salicylamine derivatives (**10a–c**). With an increase in the spacer length from (CH_2_)_4_ to (CH_2_)_8_, anti‐AChE activity increased nine times. The lead compound, salicylamine derivative **10c**, had an IC_50_ (AChE) value of 0.265 ± 0.018 μM. This was about 17 times more active than amiridine, whose IC_50_(AChE) value was 4.44 ± 0.36 μM. The results obtained, along with the close inhibitory activity of the model compound *N*‐hexyl‐amiridine **11** and amiridine (Table 2), support our hypothesis that an alkylene spacer would enhance the anticholinesterase activity of the conjugates.

In contrast, model compounds **12–14**, *N*‐hexyl derivatives of the second salicylic pharmacophore, showed no significant anticholinesterase activity.

#### 2.2.2 Kinetic studies of AChE and BChE inhibition

**Figure 3:**
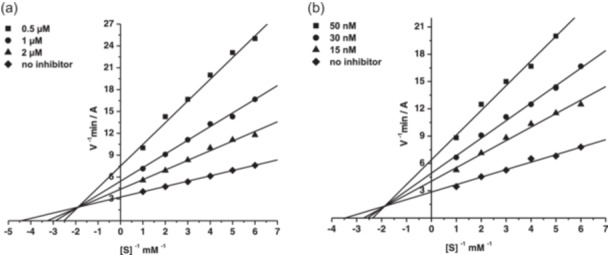
Steady‐state inhibition of acetylcholinesterase (AChE) (a) and butyrylcholinesterase (BChE) (b) by compound **10b**.

The mechanism of inhibition of AChE and BChE by the conjugates was studied using compounds **7b**, **9b,** and **10b**. Figure 3 shows Lineweaver–Burk plots of the enzyme kinetics results, taking compound **10b** as a representative example. Analysis of the plots demonstrated changes in both *K*_m_ and V_max_ values—a result consistent with a mixed type of inhibition. The values obtained for the competitive (*K*_i_) and noncompetitive (α*K*_i_) components of the constants for AChE and BChE inhibition by compounds **7b**, **9b,** and **10b** are listed in Table 3.

**Table 3:** Inhibition constants of cholinesterases by conjugates **7b**, **9b**, and **10b**.^a^

	AChE		eqBChE	
Compound	*K*_i_, µM	*αK*_i_, µM	*K*_i_, nM	*αK*_i_, nM
**7b**	2.60 ± 0.19	6.25 ± 0.44	14.2 ± 1.1	19.2 ± 0.9
**9b**	1.76 ± 0.12	4.98 ± 0.33	50.1 ± 4.8	89.0 ± 7.1
**10b**	0.656 ± 0.011	1.50 ± 0.03	21.0 ± 0.6	45.4 ± 4.1

Abbreviations: AChE, acetylcholinesterase; BChE, butyrylcholinesterase.

^a^ Values for *K*_i_ (competitive inhibition constant) and α*K*_i_ (noncompetitive inhibition constant) were determined from analyses of slopes of 1/V versus 1/S at various inhibitor concentrations. Values (means ± SEM) are from at least three separate experiments.

#### 2.2.3 Displacement of propidium from the PAS of *Ee*AChE

To evaluate the synthesized conjugates as potential inhibitors of the proaggregation activity of AChE, we used a fluorescent method to determine the ability of the compounds to competitively displace propidium, a selective ligand of the AChE PAS, responsible for binding to β‐amyloid. As can be seen from Table 2, all studied conjugates at a concentration of 20 μM displaced propidium from the *Ee*AChE PAS, reducing the fluorescence intensity at a level (10%–15%) comparable to or slightly higher than the values of the reference compound Donepezil and the original pharmacophore amiridine.

The results obtained on the displacement of propidium from the AChE PAS, along with the mixed mechanism of AChE inhibition and double‐site binding of conjugates in the catalytic active site (CAS) and PAS of AChE according to the results of molecular docking (see next Section 2.2.4), indicate that the conjugates bind to the AChE PAS and, therefore, would be expected to be able to block AChE‐induced aggregation of β‐amyloid.

#### 2.2.4 Molecular modeling: AChE and BChE inhibition

Molecular docking was performed to analyze the binding mode of the conjugates to AChE and BChE. Considering that the pKa value of the endocyclic nitrogen atom in amiridine is 9.52,^[^ ^37^ ^]^ the amiridine fragment in the conjugates should be protonated at this nitrogen atom under the experimental conditions (pH 7.5). The secondary amine group of the salicylamine moiety was fully protonated,^[^ ^17^ ^]^ while the imine nitrogen atom of salicylimine moiety (p*K*a about 6.3–6.9) was only partly protonated,^[^ ^16^, ^17^ ^]^ as it was shown in our previous works. In both AChE and BChE, the docking poses of protonated and unprotonated salicylimine conjugates were almost identical. This result may be due to the formation of an intramolecular hydrogen bond, which reduces the ability of the protonated imine group to interact with cholinesterases. In view of this, we did not present the docking results for the imine protonated conjugates **9**.

**Figure 4:**
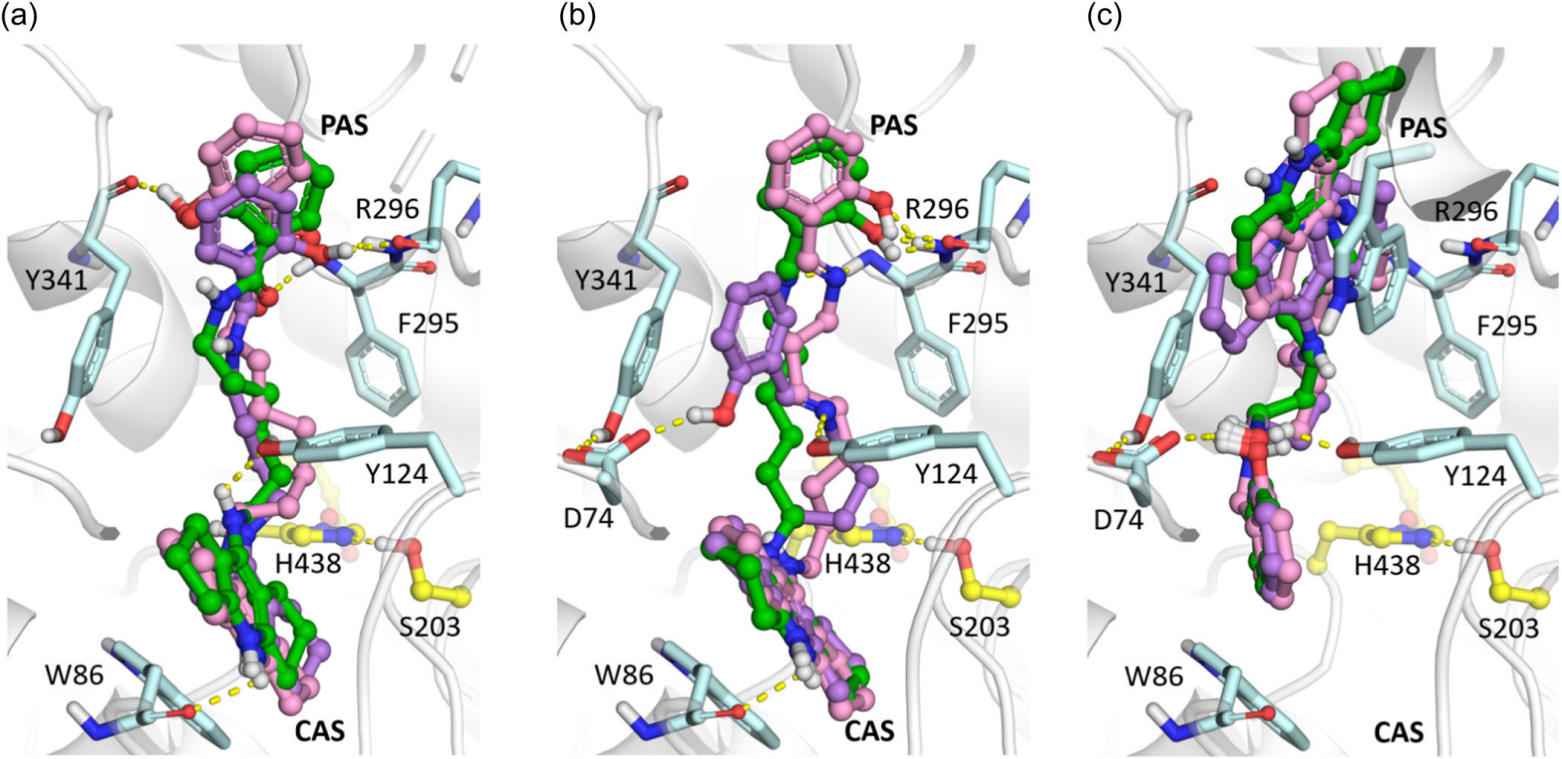
Results of molecular docking of the considered compounds to AChE: Binding poses with the strongest estimated binding affinity of compounds **7a–c** (a), **9a–c** (b), and **10a–c** (c). Carbon atoms of compounds with *n* = 4 (suffix **a**) are colored purple, *n* = 6 (suffix **b**) are colored green, and *n* = 8 (suffix **c**) are colored pink. Carbon atoms of the AChE catalytic residues are colored yellow; others are light blue. AChE, acetylcholinesterase.

With AChE, the salicylamides **7** (Figure 4a) and salicylimines **9** (Figure 4b) were bound in a similar way, with the protonated amiridine core binding in the CAS, and the salicylate fragment in the PAS. As we previously observed for other series of amiridine conjugates,^[^ ^26^, ^27^, ^28^, ^31^ ^]^ the proton of the endocyclic nitrogen atom of amiridine formed a hydrogen bond with the main chain oxygen atom of Trp86 (Figure 4a,b). The salicylamide group of compound **7a** formed hydrogen bonds with the main chain NH‐group of Phe295 and both with the main chain NH‐group and O atom of Arg296 (Figure 4a). Compounds **7b** and **7c** with longer linkers had similar interactions in the PAS; the salicylate hydroxy group interacted with the Tyr341 main chain oxygen, and the amide oxygen formed hydrogen bonds with the main chain NH groups of Phe295 and Arg296 (Figure 4a). The similar binding mode of the compounds explains similar propidium displacement efficacy from the AChE PAS for all three conjugates (Table 2). For compounds **9a–c**, the salicylimine group was bound differently: compound **9a** formed a hydrogen bond with the side chains of residues Tyr124 and Asp74 (Figure 4b), and compounds **9b,c** formed hydrogen bonds with the main chain NH groups of Phe295 and Arg296 (Figure 4b).

The binding mode of the salicylamine conjugates **10a–c** was completely different from the previous groups: the salicylamine fragment was bound in the CAS, forming hydrogen bonds interacting with side chains of Tyr124 and Asp74 (Figure 4c), and the positively charged secondary amine group formed π‐cation interactions with Tyr341. In the PAS, the amiridine core formed π–π stacking interactions with Trp286 (Figure 4c). With the increase of the linker lengths, occupation of the PAS by the amiridine fragment increased, which correlated with the increased propidium displacement ability compared with the other two groups (Table 2). In addition, compound **10c** had the best estimated binding energy to AChE, which also correlated with experimental data (Table 2).

**Figure 5:**
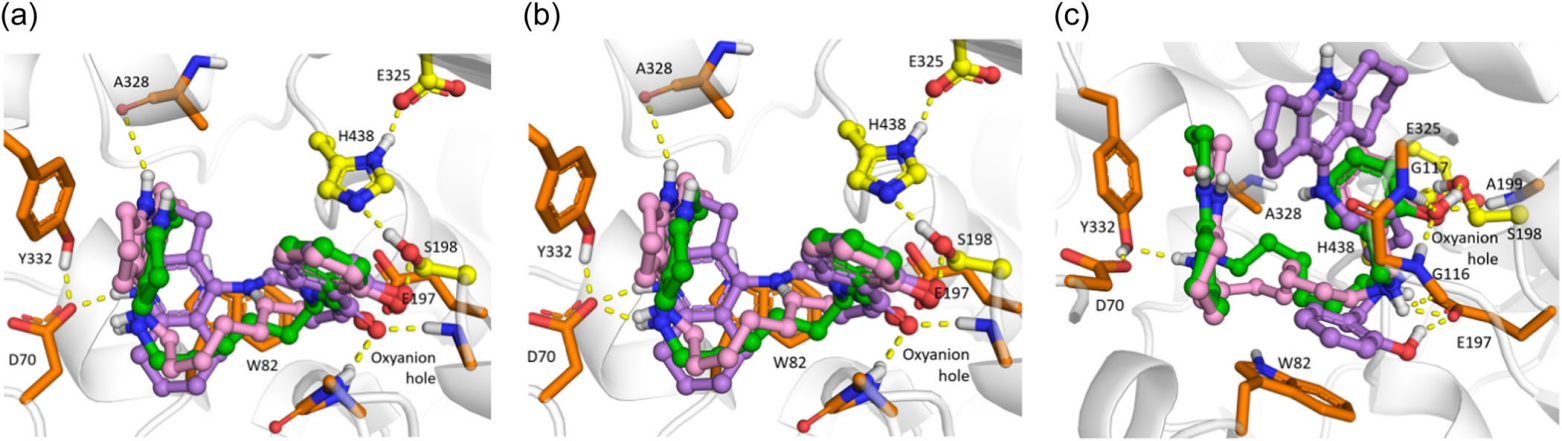
Results of molecular docking of the considered compounds to BChE: Binding poses with the strongest estimated binding affinity of compounds **7a–c** (a), **9a–c** (b), and **10a–c** (c). Carbon atoms of compounds with *n* = 4 (suffix **a**) are colored purple, *n* = 6 (suffix **b**) are colored green, and *n* = 8 (suffix **c**) are colored pink. Carbon atoms of the BChE catalytic residues are colored yellow; others are orange. BChE, butyrylcholinesterase.

In the case of docking to BChE, as for AChE, the binding modes of compounds **7a–c** and **9a–c** were similar and different from compounds **10a–c** (Figure 5). The salicylamide (Figure 5a) and salicylimine (Figure 5b) fragments were bound to the CAS, forming hydrogen bonds with the catalytic Ser198 and the oxyanion hole. The amiridine fragment of compounds **7a** and **9a** with shorter linkers formed hydrogen bonds between the protonated endocyclic nitrogen atom of amiridine and the Asp70 side chain. With the increase of the linker length, the exocyclic amino group of amiridine of compounds **7b,c** (Figures 5a) and **9b,c** (Figure 5b) formed hydrogen bonds with the Asp70 side chain, and the amiridine ring formed π–π stacking interactions with the Tyr332 side chain. With the increase of the linker length, the proton at the endocyclic nitrogen atom of amiridine also gained the ability to form a hydrogen bond with the main chain oxygen atom of Ala328.

The positively charged salicylamine group affected the binding mode of the fragment, forming an ionic pair with the Glu197 side chain (Figure 5c). The salicylamine ring of the compound **10a** with the shortest linker formed π–π stacking interactions with the Trp82 side chain, while for **10b** and **10c**, the salicylamine group was bound to the oxyanion hole and interacted with the catalytic residues. The amiridine fragment of compounds **10b** and **10c** interacted with Tyr332, forming a hydrogen bond and π–π stacking interactions (Figure 5c), but they were oriented differently compared to compounds **7b,c**, and **9b,c** (Figure 5a,b).

#### 2.2.5 Inhibition of β‐amyloid (1–42) (A**β**_42_) self‐aggregation

For conjugates **7**, **9**, **10**, the ability to inhibit self‐aggregation of β‐amyloid (1‐42) (Aβ_42_) was also studied by a fluorescent method using thioflavin T (ThT) with minor modifications. The results show (Table 2) that the studied compounds at a concentration of 100 μM inhibit the self‐aggregation of β‐amyloid at a level of 20%–96%. Inhibitory activity increased with spacer lengthening in each group. Compounds **7c**, **9c**, **10c** with spacer (CH_2_)_8_ exhibited maximum inhibitory activity against Aβ_42_ (71.7%–96.7%), which corresponds to or exceeds the effectiveness of the reference compounds myricetin (79.4 ± 6.3%) and propidium iodide (90.7 ± 7.1%).

#### 2.2.6 Molecular modeling: Interactions of conjugates with Aβ_42_

The experimental fluorescence method with thioflavin Т allows quantifying the formation of the β‐folded structure from the α‐helical soluble form of Aβ_42_ and inhibition of this process.^[^ ^43^ ^]^ For this reason, we used the monomeric α‐helical solution structure of Aβ_42_ PDB ID 1IYT as a target for molecular docking. This structure was obtained using NMR spectroscopy and contains 10 conformers, which was the result of a molecular mechanics search of structures that reproduced the NMR spectra of Aβ_42_.^[^ ^44^ ^]^ Note, in vitro, under experimental conditions (in water, at room temperature), the secondary structure of the Aβ_42_ peptide is in thermal motion and can be very different from the conformations used in docking calculations.^[^ ^45^ ^]^ Previously, we had observed that the binding of ligands to these conformers could significantly differ.^[^ ^28^ ^]^ For this reason, we used all 10 conformers as targets.

**Figure 6:**
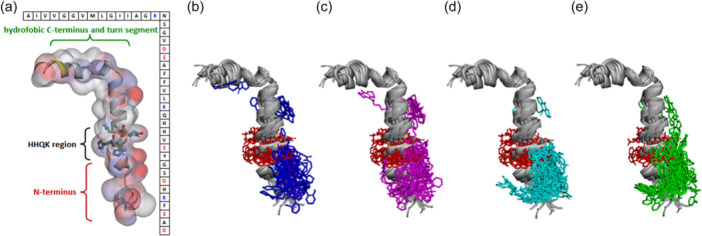
Interactions of the compounds with soluble forms of Aβ_42_. (a) A general structure of the α‐helical form Aβ_42_ with surface colored according to the charge distribution in Aβ_42_. Negatively and positively charged regions are shown in red and blue, respectively. (b–e) Binding poses of compounds **7a** (b), **7c** (c), **10a** (**d**), **7c** (e) to all 10 conformers of Aβ_42_. Amino acid residues of the HHQK region (His13, His14, Gln15, Lys16) are shown in red. Aβ, β‐amyloid.

**Figure 7:**
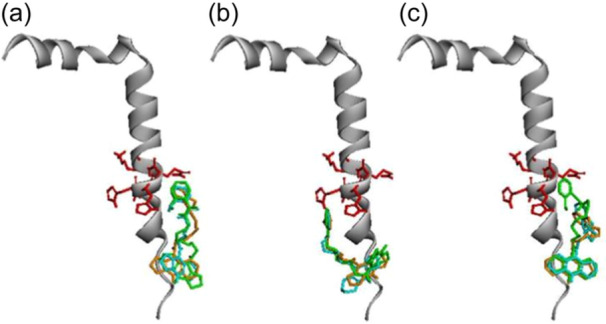
Best binding poses for conjugates of salicylamides (a), salicylimines (b) and salicylamines (c) docked to the first conformer Aβ_42_. Compounds with spacer length 4, 6, and 8 are shown in light blue, brown, and green, respectively. Amino acid residues of HHQK region (His13, His14, Gln15, Lys16) are shown in red sticks. Aβ, β‐amyloid.

The current docking results also demonstrated that the binding of the studied compounds to the individual Aβ_42_ conformers differs rather significantly (see Figure 6). To show the binding poses with the best binding energies, we chose three best poses for salicylamide and salicylamine conjugates with the shortest (**7a, 10a**) and the longest (**7c, 10c**) spacers docked to all 10 conformers of Aβ_42_. The strongest binding was observed to the first conformer of Aβ_42_. Figure 7 presents the best binding poses for all studied compounds docked to the first conformer of Aβ_42_. These poses were similar, where the amiridine fragment was bound at the N‐terminus and the salicylate pharmacophore was bound at the HHQK region.

Thus, all studied compounds preferentially were bound at the N‐terminus of the peptide structure. In addition, binding affected the HHQK domain (His13‐Lys16),^[^ ^46^ ^]^ which plays an important role in oligomerization and neurotoxicity of Aβ_42_.^[^ ^47^, ^48^ ^]^ With the increase of the linker length, coverage of this domain increased, which is in agreement with the increased anti‐aggregation potency of the compounds with linker elongation. The additional positive charge of the protonated secondary amine group of the salicylamine derivatives **10** gave them an advantage in binding and, consequently, in anti‐aggregation compared to the salicylamide **7** and salicylimine **9** derivatives.

#### 2.2.7 Antioxidant activity (AOA)

Primary AOA of the conjugates was determined using two spectrophotometric tests: the ABTS radical‐scavenging assay and the Fe^3+^‐reducing antioxidant power (FRAP) assay.

The results showed that all synthesized conjugates did not exhibit ferric‐reducing activity in the FRAP test as observed for the corresponding tacrine derivatives,^[^ ^16^ ^]^ while the results of the ABTS test demonstrate their high antiradical activity, which depends on the structure of the second pharmacophore (Table 4).

**Table 4:** Radical‐scavenging activity of conjugates **7**, **9**, **10** in the ABTS assay.

No	Compound 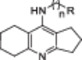		ABTS^•+^‐ scavenging activity	
	R	n	TEAC^a^	IC_50_, µM^b^
Salicylamides				
**7a**	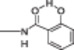	4	0.97 ± 0.04	17.6 ± 0.8
**7b**		6	0.98 ± 0.04	17.6 ± 0.7
**7с**		8	0.94 ± 0.04	19.4 ± 0.6
Salicylimines				
**9a**	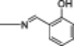	4	0.65 ± 0.03	26.6 ± 1.1
**9b**		6	0.64 ± 0.03	28.2 ± 1.3
**9с**		8	0.63 ± 0.02	29.6 ± 1.2
Salicylamines				
**10a**	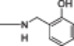	4	1.5 ± 0.08	10.1 ± 0.5
**10b**		6	1.48 ± 0.07	9.5 ± 0.6
**10с**		8	1.48 ± 0.05	10.1 ± 0.6
Amiridine			0.040 ± 0.003	n.d.
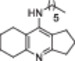 **11**			n.a.	n.d.
Salicylic acid			n.a.	n.d.
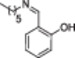 **12**			0.15 ± 0.008	93.3 ± 4.1
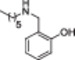 **13**			1.52 ± 0.07	10.7 ± 0.4
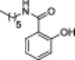 **14** ^[^ ^35^ ^]^			0.39 ± 0.04	46.5 ± 2.3
Trolox			1.0	19.7 ± 1.4

*Note*: Data are expressed as mean ± SEM, *n* = 3.

Abbreviations: ABTS, 2,2′‐azino‐bis(3‐ethylbenzothiazoline‐6‐sulfonic acid); n.a., inactive; n.d., not determined; TEAC, Trolox equivalent antioxidant capacity.

^a^ TEAC—activity of the compounds relative to Trolox (for calculation, see Section 4).

^b^ IC_50_—concentration of the compound required to reduce the concentration of the ABTS radical by 50%.

As can be seen from Table 4, salicylimine derivatives **9a–c** were quite active in the ABTS test (Trolox equivalent antioxidant capacity [TEAC] = 0.64 ± 0.01), while changing the length of the spacer of conjugates did not affect the activity of compounds. We observed significant changes when replacing the imine spacer with an amine one (compounds **10a–c**), whereupon the radical‐scavenging activity increased by 2.3 times (TEAC = 1.50 ± 0.07). A rather high initial rate of the ABTS^•+^‐binding reaction was also observed (within 1 min).

Conjugates of amiridine and salicylamide **7a–c** exhibited higher ABTS^•+^‐scavenging activity (TEAC = 0.94–0.98) than that of salicylimines (TEAC = 0.64), close to the activity of the standard antioxidant Trolox (TEAC = 1.00), but they were less active compared to salicylamine analog.

As for model compounds **12–14** based on salicylic derivatives, *N*‐hexylsalicylamine **13** demonstrated the highest radical‐scavenging (ABTS^•+^‐scavenging) activity (TEAC = 1.52 ± 0.07), equal to the activity of the corresponding conjugates, and a rather high initial rate of the ABTS^•+^‐ binding reaction (within 1 min), while its salicylimine analog **12** had a radical‐scavenging activity that was 10‐fold lower (TEAC = 0.15 ± 0.008). Activity of *N*‐hexylsalicylamide **14** was 2.6 times higher (TEAC = 0.39 ± 0.04),^[^ ^35^ ^]^ while salicylic acid itself did not show activity in the studied concentration range (from 1 to 100 μM), which is consistent with literature data.^[^ ^33^ ^]^ Interestingly, the model compounds demonstrated the same range of activities as their conjugates, while the effect of structure changes was more pronounced.

Thus, amiridine conjugates with salicylic acid derivatives, as the previously studied tacrine conjugates,^[^ ^16^ ^]^ demonstrated high radical‐scavenging activity in the ABTS test and the absence of Fe^3+^‐reducing activity in the FRAP test. ABTS^•+^‐scavenging activity depended on the structure of the salicylic derivative fragment. The lead compounds were conjugates of salicylamine **10a–c**.

#### 2.2.8 Quantum chemical analyses of AOA

Quantum chemical calculations (Supporting Information S1: Tables S1–S2 for details) showed that under ABTS assay conditions, conjugates of salicylamide **7** and salicylimine **9** are protonated once at the endocyclic N‐atom of the amiridine moiety (marked with the subscript “a”), and conjugates of salicylamine **10** are protonated twice: at the endocyclic N‐atom of the amiridine moiety and at the amine N‐atom of the salicylic moiety (marked with the subscript “s”).

Given that in the experiment no dependence of AOA on the spacer length was observed, one compound of each type with the shortest spacer (CH_2_)_4_, namely, **7a**_**a**_, **9a**_**a**_, **10a**_**as**_ (here subscripts mark protonation states as described above) was taken for calculations.

Quantum chemical calculations showed that the bond dissociation enthalpy (BDE) of the OH group agrees with the AOA of the compounds under study (see Table 5). The lowest BDE value was found for compound **10a**_**as**_ (83.5 kcal/mol) with the highest AOA (TEAC = 1.5), and the highest BDE value was found for compound **9a**_**a**_ (90.2 kcal/mol) with the lowest AOA (TEAC = 0.65).

**Table 5:** Calculated BDE values for the investigated compounds.

Compound	7a_a_	9a_a_	10a_as_
**BDE, kcal/mol**	87.4	90.2	83.5

Abbreviation: BDE, bond dissociation enthalpy.

**Figure 8:**
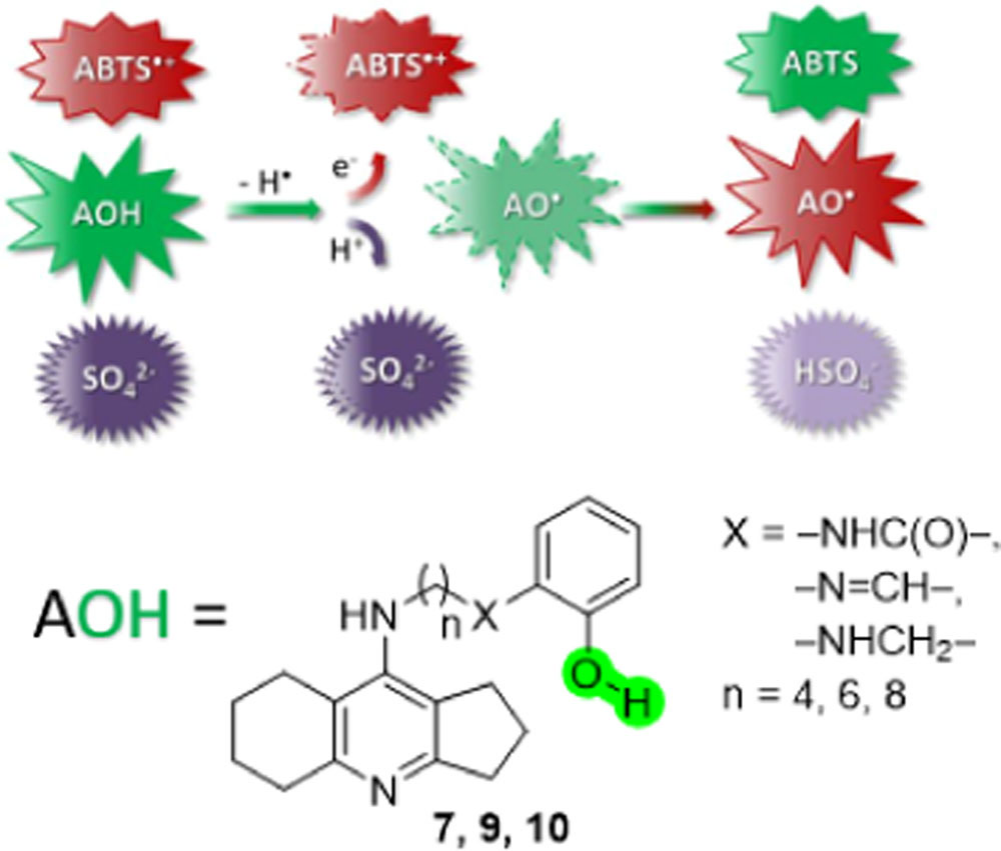
Scheme of ABTS^•+^ reactions with amiridine‐salicylate conjugates. The antioxidant molecule (AOH) loses an H‐atom, so that the electron is transferred to the ABTS^•+^ cation‐radical, and the proton is simultaneously transferred to the SO_4_^2‐^ anion of the solvent. ABTS, 2,2′‐azino‐bis(3‐ethylbenzothiazoline‐6‐sulfonic acid).

It can be assumed that the mechanism of the antiradical action of the studied compounds is H‐atom abstraction from the OH group with electron transfer to the ABTS^•+^ radical and simultaneous proton loss to the solvent (see Figure 8). Note that the proton acceptors in the solvent are SO_4_^2–^ anions formed during the generation of ABTS^•+^ radicals (one SO_4_^2‐^ anion per one ABTS^•+^ radical).^[^ ^49^ ^]^

#### 2.2.9 Metal‐chelating properties

UV‐Vis spectroscopy was used to evaluate the ability of compounds to form metal complexes, as shown previously by other groups^[^ ^50^, ^51^ ^]^ and by us.^[^ ^16^ ^]^ In the present work, the ability of compounds **7c**, **9c**, **10c**, which belong to three different groups of conjugates, to bind Cu^2+^, Fe^2+^, and Zn^2+^ ions in EtOH solution was studied. The results are shown in Figures 9, 10, 11, Supporting Information S1: Figures S2–S55, and Table 6.

**Figure 9:**
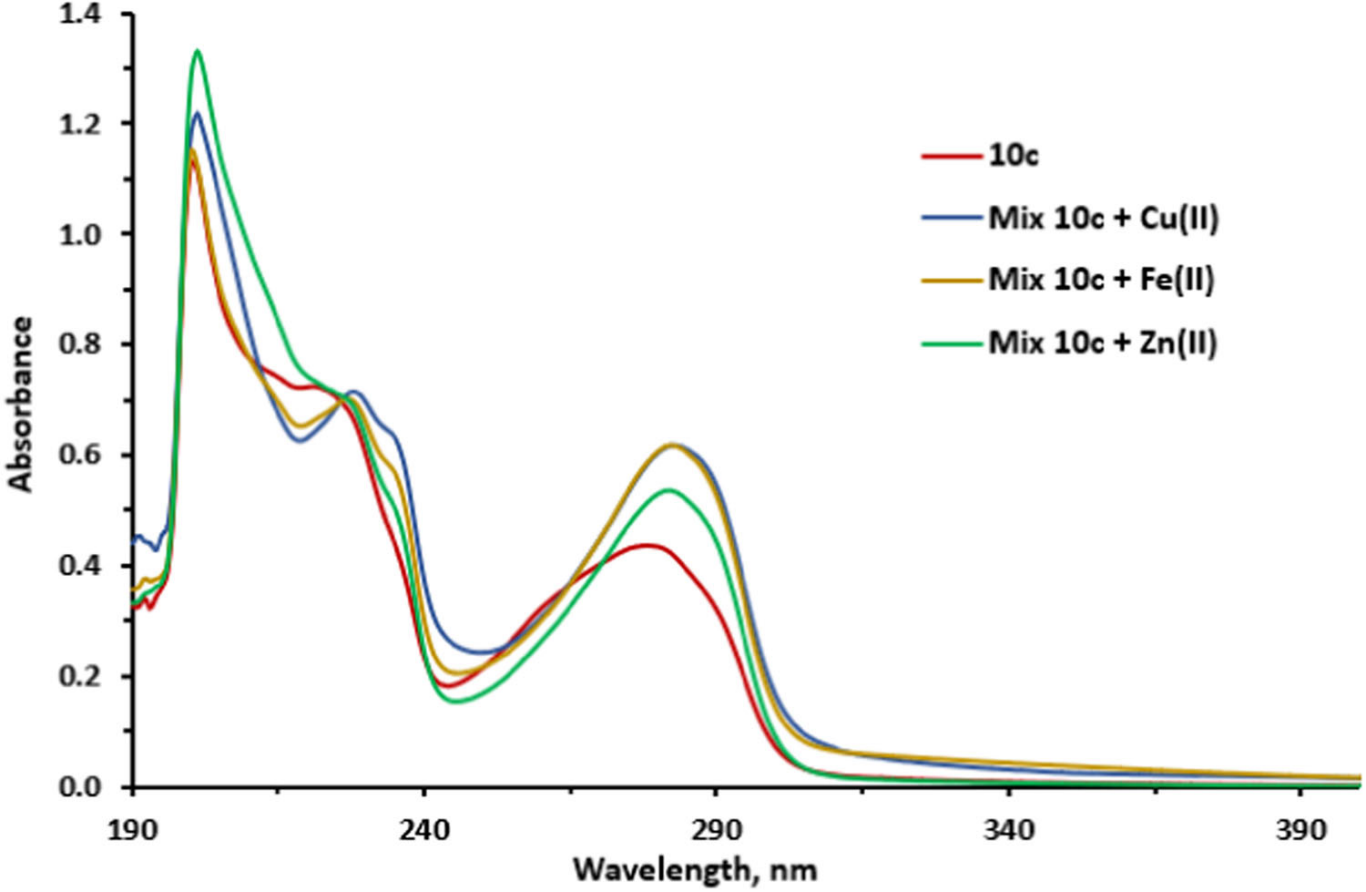
UV spectra of compound **10c** and its mixtures with Cu^2+^, Fe^2+^, and Zn^2+^ ions.

**Figure 10:**
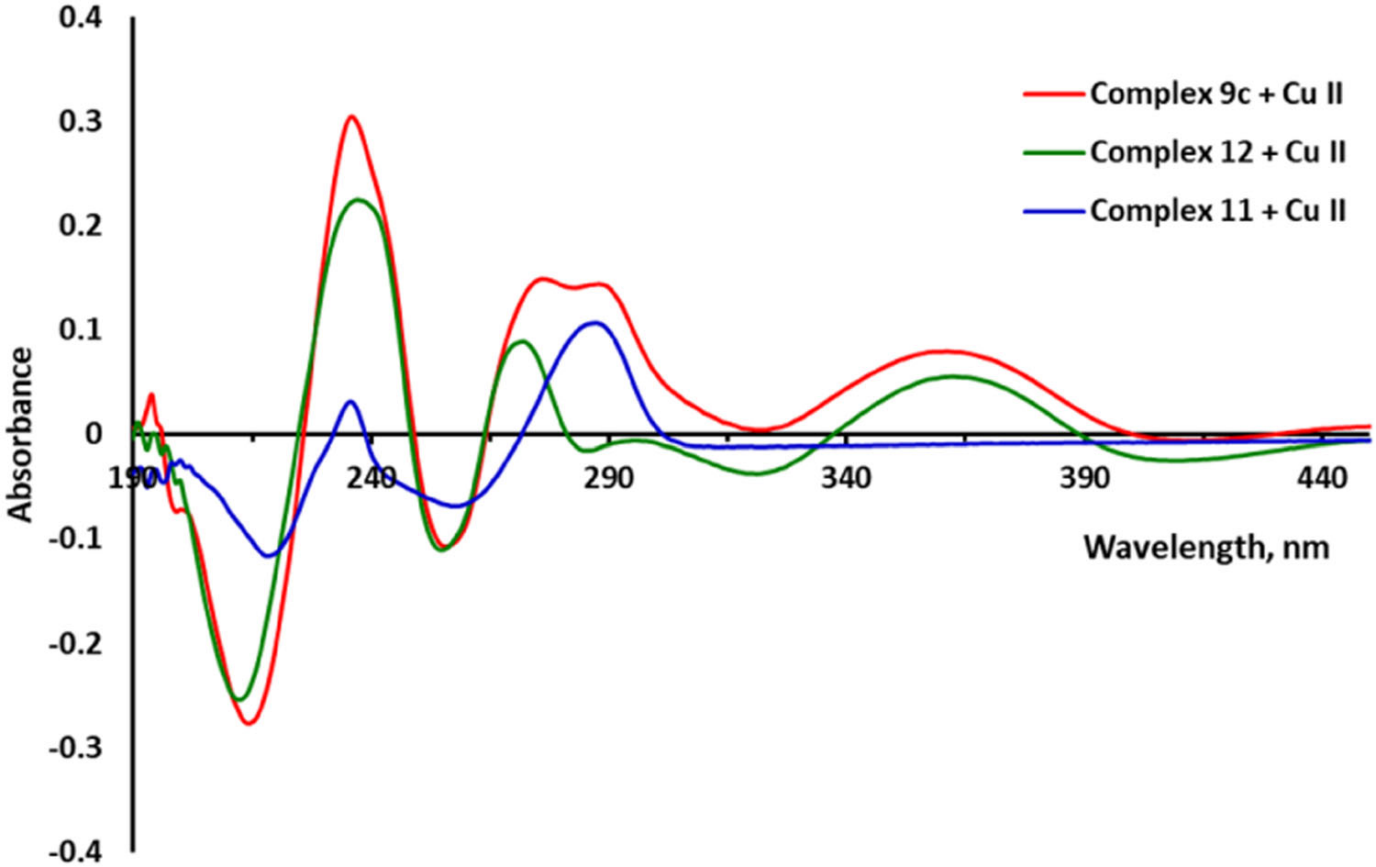
UV spectra of the **9c**–Cu^2+^, **11**–Cu^2+^, and **12**–Cu^2+^ complexes were obtained by subtracting the spectra of ions and compounds from the spectra of mixtures.

**Figure 11:**
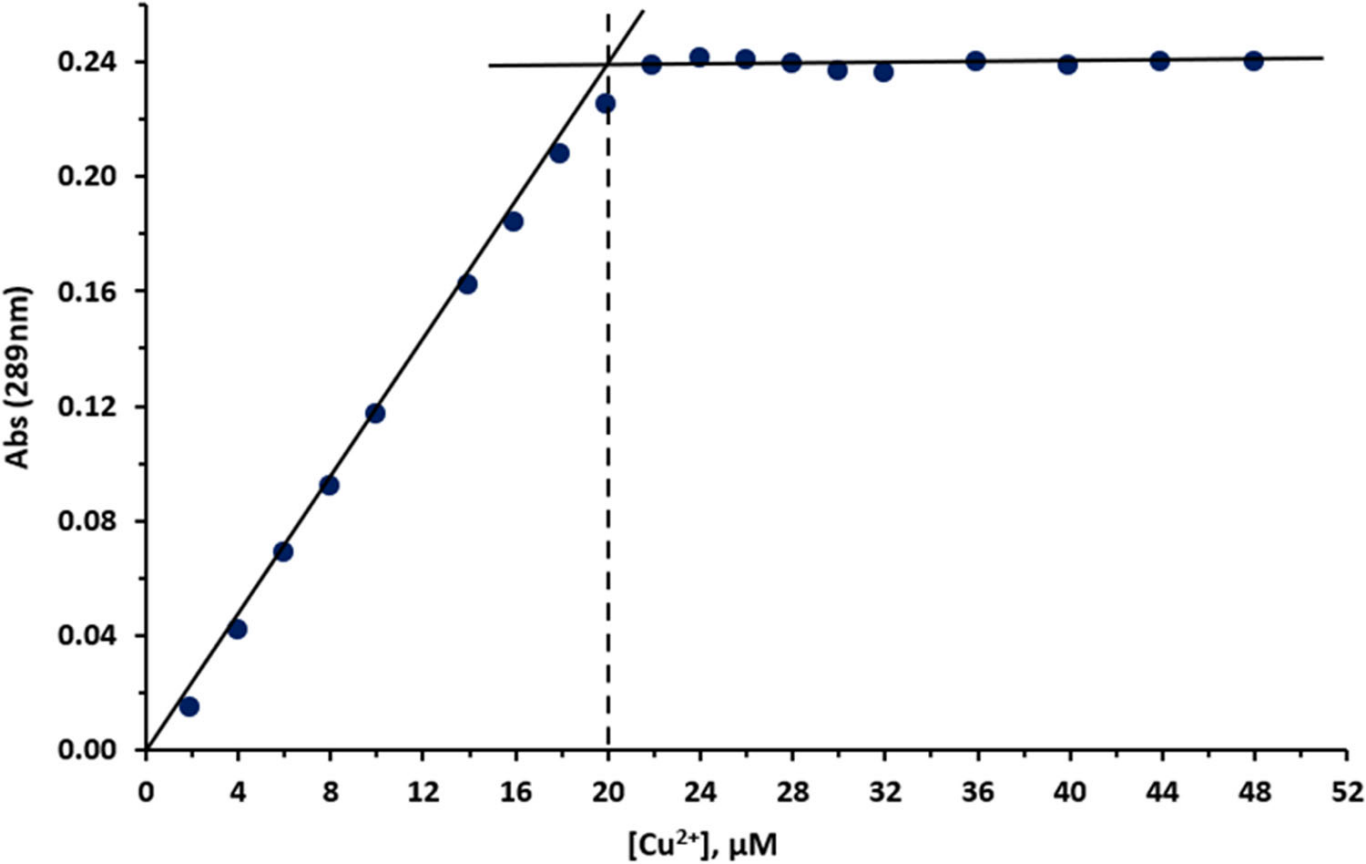
Absorbance of the **10c**–Cu^2+^ complex at 352 nm as a function of the concentration of Cu^2+^ (compound's concentration is 20 µM). Vertical dashed lines mark the metal concentration at the breakpoints and indicate a ligand–metal molar ratio of 1:1.

**Table 6:** Data on UV spectra of **7c**, **9c**, **10c** and their metal complexes.

Compound	λ_max_, nm	Shift of Absorbance,^a^ nm		
		Cu(II)	Zn(II)	Fe(II)
**7c**	286; 228; 205	288; 236	288; 236	288; 236
**9c**	317; 279; 259; 214	363; 289; 276; 236	287; 236	287; 236
**10c**	278; 220; 201	288; 236	287; 236	287; 236
**11**	277; 224	287; 236	287; 236	287; 236
**12**	400; 316; 254; 215	362; 295; 272; 237	‐	‐

^a^ The shift of absorbance values was calculated by subtraction of the sum of spectra of the compound and the corresponding ion from the spectra of the mixture.

The UV absorption spectra of compounds **7c**, **9c**, **10c** contain two groups of bands. The long‐wavelength absorption band at 270–400 nm probably corresponds to π–π* transitions. In the absorption spectra of solutions of mixtures of conjugates **7c**, **9c**, **10c** with Cu^2+^, Fe^2+^, Zn^2+^, the greatest red shift of absorption bands (up to 11 nm) was observed in the case of conjugate **10c**, which has an amine residue (Figure 9, Table 6).

The absorption spectrum of salicylimine **9c** is noteworthy, as it lacks the usual bathochromic shift of the absorption bands (Supporting Information S1: Figure S48b). Comparison of the absorption spectra of the **9c**–Cu^2+^, **11**–Cu^2+^, and **12**–Cu^2+^ complexes showed that **9c**–Cu^2+^ is a superposition of the absorption spectra of **11**–Cu^2+^ and **12**–Cu^2+^, indicating that copper ions were bound by both pharmacophores of the conjugate (Figure 10). In the case of **7c**–Cu^2+^ and **10c**–Cu^2+^, binding occurred predominantly with the amiridine part of the conjugate. Specific details of the coordination of metal ions require further study.

The ligand/metal ion ratio in the Cu^2+^ complexes was investigated for conjugates **7c**, **9c**, and **10c** by mixing a fixed concentration of the test compound with increasing concentrations of the metal ion (2–50 µM)^[^ ^52^ ^]^ (Figure 11 and Supporting Information S1: S51–S54). According to the titration curves (Figure 11), the molar ratio of **10c** to Cu^2+^ in the complex was 1:1. For the **7c**–Cu^2+^ complex, the ligand/metal ratio was also ~1:1 (Supporting Information S1: Figure S54a). In the case of the **9c**–Cu^2+^ complex, the ligand/metal ratio was almost 2:1, which may also indicate a difference in the coordination of compound **9c** with Cu^2+^ compared to conjugates **7c** and **10c** (Supporting Information S1: Figure S54b).

According to the obtained results, the conjugates **7c**, **9c**, **10c** can chelate Cu^2+^, Fe^2+^, and Zn^2+^ ions, which indicates the prospects for further study of their chelating properties.

#### 2.2.10 Cytotoxicity studies

Сompounds **7a**, **9a**, and **10a** with spacer (CH_2_)_4_ and **7c**, **9c**, and **10c** with spacer (CH_2_)_8_, respectively, were chosen for determining their safety profiles as assessed by cytotoxicity toward three different cell lines. Amiridine was included as a reference compound. Cell viability assays on human embryonic kidney (HEK293T), human hepatocellular carcinoma (HepG2), and human neuroblastoma cells (SH‐SY5Y) were performed using the MTT method.^[^ ^53^, ^54^, ^55^ ^]^ The IC_50_ values (concentration of the compound resulting in a 50% decrease in cell viability) are listed in Table 7 and Supporting Information S1: Figures S56–S61.

**Table 7:** Cytotoxic effect toward HEK293T, HepG2, and SH‐SY5Y cell lines for conjugates of amiridine and salicylic derivatives **7a**,**c**, **9a**,**c** and **10a**,**c**.^a^

Compound	HEK293T IC_50_, μM	Hep G2 IC_50_, μM	SH‐SY5Y IC_50_, μM
**7a**	38.4 ± 3.6	74.7 ± 3.4	49.6 ± 3.7
**7c**	3.62 ± 0.02	6.92 ± 1.22	7.99 ± 0.30
**9a**	38.1 ± 2.1	108.8 ± 7.1	73.2 ± 0.9
**9c**	22.2 ± 2.2	95.9 ± 5.4	63.5 ± 4.4
**10a**	4.49 ± 0.27	27.6 ± 1.3	90.8 ± 1.3
**10c**	2.33 ± 0.15	4.19 ± 0.20	10.9 ± 0.6
Amiridine	106.1 ± 3.0	224.5 ± 11.2	135.0 ± 18.2

^a^ Data are expressed as mean ± SEM, *n* = 3.

By this criterion, the least toxic compounds across all three cell types were amiridine, **7a**, **9a**, and **9c**, whereas the most toxic compounds were **7c** and **10c**. Within each cell type, compound **7a** with a (CH_2_)_4_ spacer was less toxic than compound **7c** with a (CH_2_)_8_ spacer. The same trend was observed for **9a** versus **9c** and **10a** versus **10c**. The apparent trend of increasing cytotoxicity of the conjugates **7c**, **9c**, and **10c** with spacer (CH_2_)_8_ and **7a**, **9a**, and **10a** with spacer (CH_2_)_4_ compared with each other and to the reference compound amiridine may be due to the long length of the octamethylene spacer, which increases the lipophilicity of the studied compounds and, consequently, their cytotoxicity.^[^ ^56^ ^]^

#### 2.2.11 Prediction of ADMET, physicochemical, and Pan Assay INterference compoundS (PAINS) profiles

The results of our computational estimates of selected ADMET and physicochemical properties for compounds **7**, **9,** and **10** are shown in Table 8. Most of the compounds had high predicted values for intestinal absorption, enabling their oral administration. Moreover, we could expect reasonable CNS activity in view of rather high predicted blood–brain barrier permeability (brain concentration is about 25%–220% of the plasma concentration). The cardiac toxicity risk parameters (hERG p*K*_*i*_ and pIC_50_) fell within 3.9–6.0 log units for all the analyzed compounds, which was within the lower and medium parts of their possible range (3–9 log units). According to the commonly accepted drug‐likeness guidelines, the predicted lipophilicities and aqueous solubilities, as well as the molecular weights of the compounds, were within or close to the desirable range for potential drug compounds, although the LogP values in some cases violated the original Rule‐of‐5 limits (however, given that some of the compounds were outside of the model applicability domain, the predicted values were not fully reliable). The integral quantitative estimates of drug‐likeness (QED) were in the 0.3–0.6 range. The PAINS filter check revealed the “mannich_A(296)” alert for compounds **10a–c**.

**Table 8:** Predicted ADMET and physicochemical profiles of compounds **7**, **9**, and **10**.

Compound	MW	LogP_ow_	pS_aq_	LogBB	HIA, %	hERG *pK*_*i*_	hERG *pIC*_*50*_	QED
**7a**	379.50	4.06	4.63	−0.61	85	4.02	5.44	0.64
**7b**	407.56	4.75	5.50	−0.62	85	3.92	5.55	0.53
**7c**	435.61	5.42	6.20	−0.24	85	4.29	5.98	0.42
**9a**	363.50	4.47	4.89	−0.47	79	4.23	5.15	0.56
**9b**	391.56	5.42	5.63	−0.55	79	4.19	5.54	0.45
**9c**	419.61	6.18	6.24	−0.17	79	4.55	5.96	0.35
**10a**	365.52	3.76	3.75	−0.02	81	4.65	5.29	0.62
**10b**	393.57	4.58	4.51	−0.02	81	4.47	5.42	0.50
**10c**	421.63	5.30	5.26	0.35	81	4.83	5.80	0.38
Amiridine	188.27	2.62	1.75	−0.58	92	4.34	4.44	0.68

Abbreviations: ADMET, absorption, distribution, metabolism, elimination, and toxicity; HIA, human intestinal absorption [%]; hERG *pKi,* hERG potassium channel affinity [−log(M)]; hERG *pIC*_*50*_, hERG potassium channel inhibitory activity [−log(M)]; LogP_ow_, octanol‐water partition coefficient; LogBB, blood–brain barrier distribution; MW, molecular weight; pS_aq_, aqueous solubility [−log(M)]; QED, quantitative estimate of drug‐likeness.

Consequently, the predicted ADMET, physicochemical, and PAINS properties of the compounds were acceptable for potential lead compounds in the discovery phase. Nevertheless, additional studies and structure optimization would be desirable to help maximize safety and improve the pharmacokinetic profile.

## 3 CONCLUSION

A method has been developed for replacing the chlorine atom in chloramiridine with the amino group of diaminoalkane, which for the first time made it possible to introduce an aminopolymethylene spacer into the amiridine molecule and to obtain 3 groups of its conjugates with salicylic derivatives (salicylamide **7**, salicylimine **9,** and salicylamine **10**) containing different lengths of alkylene spacers.

A study of the esterase profile of conjugates **7**, **9**, and **10** showed that all of them effectively inhibited AChE and BChE at the same or much higher level as the parent pharmacophore amiridine, with selectivity toward BChE. Salicylamide conjugates **7b** and **7c** had maximum BChE/AChE selectivity indices of 193 and 138, respectively. All the conjugates rather weakly inhibited the off‐target CES, suggesting a low probability to exert unwanted drug–drug interactions in clinical use.

Increasing the spacer length from (CH_2_)_4_ to (CH_2_)_8_ led to an increase in inhibition activity against AChE and especially BChE. Salicylamide derivative **7c** with spacer (CH_2_)_8_, inhibited BChE with IC_50_ = 11.1 ± 0.1 nM, which was 25 times more effective than amiridine.

The conjugates were mixed‐type reversible inhibitors of both cholinesterases and demonstrated dual binding to the catalytic and PASs of AChE in molecular docking, which along with experimental results on propidium displacement suggested their potential to block AChE‐induced β‐amyloid aggregation.

All conjugates were active as inhibitors of Aβ_42_‐self‐aggregation in the thioflavin test. Inhibitory activity substantially increased with spacer length in each group. Compounds **7c**, **9c**, and **10c** with spacer (CH_2_)_8_ exhibited maximum inhibitory activity (71.7%–96.7%) at the same or higher level than the reference compounds myricetin (79.4 ± 6.3%) and donepezil (90.7 ± 7.1). These results agreed with molecular docking to Aβ_42_, which showed binding of conjugates **7c**, **9c**, and **10c** with the HHQK domain of Aβ_42_.

The conjugates demonstrated high antiradical activity in the ABTS test. Quantum chemical calculations showed that the AOA of conjugates was determined by the BDE values of the OH group of the salicylate pharmacophores. The most active were salicylamine derivatives **10a‐c** (TEAC about 1.5).

The conjugates showed the ability to bind Cu^2+^, Fe^2+^, and Zn^2+^ ions.

Cytotoxicity assessment toward three different cell lines: human embryonic kidney (HEK293T), human hepatocellular carcinoma (HepG2), and human neuroblastoma cells (SH‐SY5Y) showed that the less toxic compounds were conjugates with salicylimine. Conjugates **7c**, **9c**, and **10c** with spacer (CH_2_)_8_ exhibited a trend of higher toxicity than their analogs with spacer (CH_2_)_4_, which could be due to their higher lipophilicity.

Most of the conjugates had high predicted values for intestinal absorption, enabling their oral administration. Moreover, reasonable CNS activity could be expected in view of rather high predicted blood–brain barrier permeability.

Taken together, the results indicate that the new conjugates show promise as potential multifunctional anti‐AD drug candidates for further development.

## 4 EXPERIMENTAL

### 4.1 Chemistry

#### 4.1.1 General

Melting points were determined in open capillaries on a Stuart SMP30 melting point apparatus (Bibby Scientific Limited) and were uncorrected. The IR spectra were recorded on a Perkin Elmer Spectrum Two instrument (PerkinElmer) using a frustrated total internal reflection accessory with a diamond crystal. The ^1^H NMR spectra were registered on a Bruker DRX‐400 spectrometer (400 MHz) or a Bruker Avance^III^ 500 (500 MHz) (Bruker). The ^13^C NMR spectra (see the Supporting Information S2) were recorded on a Bruker Avance^III^ 500 spectrometer (125 MHz). The internal standard was SiMe_4_. The IR and NMR spectra of compounds **2**, **3**, **5**, **7**, **9–13** are shown in Supporting Information S1: Figures S1–S47. The microanalyses (C, H, N) were carried out on a PerkinElmer PE 2400 series II elemental analyzer (PerkinElmer). The high‐resolution mass spectrometry (HRMS) was performed using a Bruker Daltonics MaXis Impact HD (Bruker) quadrupole time‐of‐flight mass spectrometer with positive electrospray ionization from MeOH or MeCN solutions, flow rate 0.25 or 0.35 mL·min^−1^ with parameters optimized for small molecules detection based on a preinstalled method for infusion analysis. The column chromatography was performed on silica gel 60 (0.062–0.2 mm) (Macherey‐Nagel GmbH & Co KG).

The X‐ray diffraction study was performed on an Xcalibur 3 CCD diffractometer with a graphite monochromator, λ(MoKα) 0.71073 Å radiation, T 295(2) K. An empirical absorption correction was applied. The structure was solved with the SHELXT^[^ ^57^ ^]^ (for **9а**) or Superflip^[^ ^58^, ^59^, ^60^ ^]^ (for **9с**) and SHELXL^[^ ^57^ ^]^ structure solution program and refined with Olex2.^[^ ^61^ ^]^ All nonhydrogen atoms were refined in the anisotropic approximation; H‐atoms at the C H bonds were refined in the “rider” model with dependent displacement parameters. An empirical absorption correction was conducted through spherical harmonics, implemented in the SCALE3 ABSPACK scaling algorithm by a program “CrysAlisPro” (Rigaku Oxford Diffraction). Complete crystallographic data for compounds **9а** (CCDC 2370212) and **9с** (CCDC 2370211) are available at https://www.ccdc.cam.ac.uk/structures.

Ethanol, chloroform, methylene chloride, salicylic acid, ammonium hydroxide, potassium iodide, sodium hydroxide, diethyl ether, dimethylformamide, toluene, sodium borohydride, triethylamine, and sodium sulfate were obtained from VEKTON AO. Hexane was obtained from EKOS‐1. Pentanol‐1, 1,4‐diaminobutane, 1,6‐diaminohexane, 1,8‐diaminooctane, hexylamine, and salicylaldehyde were purchased from Alfa Aesar via Thermo Fisher Scientific. The deuterated solvents DMSO‐*d*_6_ and CDCl_3_ were acquired from SOLVEX LLC (Skolkovo Innovation Center). All solvents, chemicals, and reagents were used without purification.

Amiridine **1**,^[^ ^62^ ^]^ 1,2,3,4,5,6,7,8‐octahydro‐9*H*‐cyclopenta[*b*]quinolin‐9‐one (**2**),^[^ ^36^ ^]^ 9‐chloro‐2,3,5,6,7,8‐hexahydro‐1*H*‐cyclopenta[*b*]quinolone (**3**)^[^ ^37^ ^]^ were synthesized by referring to the previously published method.

The InChI codes of the investigated compounds, together with biological activity data, are provided as Supporting Information S2.

#### 4.1.2 Characteristics of the starting compounds **2**, **3**

1,2,3,4,5,6,7,8‐Octahydro‐9*H*‐cyclopenta[*b*]quinolin‐9‐one (2): M.p.: >300°C (with decomposition) (lit.^[^ ^36^ ^]^ M.p.: 382–384°C, with decomposition and partial sublimation), white powder. ^1^H NMR (400 MHz, DMSO‐*d*_6_): *δ* = 1.61–1.68 (m, 4H, 2CH_2_); 1.93 (p, *J* = 7.5 Hz, 2H, CH_2_); 2.25–2.28 (m, 2H, CH_2_); 2.50–2.53 (m, 4H, 2CH_2_, overlapping with a solvent residual peak); 2.76 (t, *J* = 7.6 Hz, 2H, CH_2_); 11.16 (br. s, 1H, NH). ^13^C NMR (125 MHz, DMSO‐*d*_6_): δ = 21.41, 21.63, 21.69, 21.96, 26.42, 27.57, 30.96, 121.04, 123.55, 142.78, 148.88, 175.50.

9‐Chloro‐2,3,5,6,7,8‐hexahydro‐1*H*‐cyclopenta[*b*]quinolone (3): M.p.: 37–38°C (lit.^[^ ^37^ ^]^ M.p.: 38–39°C), yellow crystallizing oil. ^1^H NMR (400 MHz, CDCl_3_): δ = 1.82–1.87 (m, 4H, 2CH_2_); 2.12 (p, *J* = 7.6 Hz, 2H, CH_2_); 2.72–2.75, 2.88–2.90 (both m, 4H, 2CH_2_), 2.94 (t, *J* = 7.5 Hz, 2H, CH_2_); 3.02 (t, *J* = 7.7 Hz, 2H, CH_2_). ^13^C NMR (125 MHz, CDCl_3_) *δ* = 22.31, 22.57, 22.71, 26.15, 30.09, 32.86, 34.84, 127.26, 132.94, 141.16, 156.79, 162.85. Anal. calcd. for C_12_H_14_ClN: C 69.39, H 6.79, N 6.74. Found: С 69.27, Н 6.87, N 7.01.

#### 4.1.3 General procedure for the synthesis of amines **5a–c**

A mixture of 9‐chloro‐2,3,5,6,7,8‐hexahydro‐1*H*‐cyclopenta[*b*]quinoline 3 (0.025 mol), diamine **4а–с** (0.125 mol) and KI (0.0001 mol) in pentanol‐1 (30 mL) was stirred in a stainless‐steel closed vessel at 180–190°C for 16 h in an argon atmosphere. Then the solvent and diamine were distilled (for **5c** under reduced pressure), chloroform (100 mL) was added to the reaction mixture, the organic layer was washed with a 10% (w/v) NaOH solution (3 × 100 mL), water (3 × 100 mL), dried over anhydrous Na_2_SO_4_ and evaporated. The residue was purified by column chromatography (eluent CHCl_3_/EtOH/NH_4_OH 10:1:0.1 → 5:1:0.1). The resulting oil was treated with Et_2_O, the resulting precipitate was filtered and dried.

*N* ^1^‐(2,3,5,6,7,8‐Hexahydro‐1*H*‐cyclopenta[*b*]quinolin‐9‐yl)butane‐1,4‐diamine (**5a**): Yield 5.698 g, 88%. М.p: 81–83°C, white powder. IR (*ν*, cm^−1^): 3392, 3221, 2926, 2851 (NH, CH), 1574, 1526, 1439, 1363, 1340 (C═C, C═N). ^1^H NMR (500 MHz, CDCl_3_): *δ* = 1.31–1.38, 1.42–1.49 (both m, 4H, 2CH_2_), 1.65–1.75 (m, 4H, 2CH_2_), 1.91 (p, *J* = 7.5 Hz, 2H, CH_2_), 2.32 (t, *J* = 5.9 Hz, 2H, CH_2_), 2.53 (t, *J* = 6.8 Hz, 2H, CH_2_), 2.57–2.64 (m, 4H, 2CH_2_), 2.95 (t, *J* = 7.2 Hz, 2H, CH_2_), 3.27–3.32 (m, 2H, CH_2_), 5.05 (t, *J* = 6.2 Hz, 1H, NH), the signal of NH_2_ group was not observed due to deuterium exchange with solvent. ^13^C NMR (125 MHz, CDCl_3_) *δ* = 22.53, 22.59, 22.71, 23.34, 28.36, 30.18, 30.62, 32.58, 33.41, 41.34, 43.76, 113.42, 114.46, 149.02, 153.22, 162.33. Anal. calcd. for C_16_H_25_N_3_: С 74.09, Н 9.71, N 16.20. Found: С 73.91, Н 9.64, N 16.01. HRMS *m/z* 260.2122 [M+H]^+^ (calcd. for C_16_H_26_N_3_ 260.2121).

*N* ^1^‐(2,3,5,6,7,8‐Hexahydro‐1*H*‐cyclopenta[*b*]quinolin‐9‐yl)hexane‐1,6‐diamine (**5b**): Yield 5.453 g, 76%. М.p: 102–104°C, white powder. IR (*ν*, cm^−1^): 3346, 3263, 2925, 2852 (NH, CH), 1570, 1516, 1438, 1366 (C═C, C═N). ^1^H NMR (500 MHz, CDCl_3_) *δ* = 1.26–1.33 (m, 6H, 3CH_2_), 1.40–1.45 (m, 2H, CH_2_), 1.64–1.75 (m, 4H, 2CH_2_), 1.91 (p, *J* = 7.5 Hz, 2H, CH_2_), 2.31 (t, *J* = 5.9 Hz, 2H, CH_2_), 2.58 (t, *J* = 5.9 Hz, 2H, CH_2_), 2.62 (t, *J* = 7.7 Hz, 2H, CH_2_), 2.94 (t, *J* =7.2 Hz, 2H, CH_2_), 3.29 (dd, *J* = 14.0, 6.7 Hz, 2H, CH_2_), 4.99 (t, *J* = 6.3 Hz, 1H, NH), the signal of NH_2_ group was not observed due to deuterium exchange with solvent. ^13^C NMR (125 MHz, CDCl_3_) *δ* = 22.52, 22.59, 22.69, 23.34, 25.87, 26.06, 30.63, 30.76, 30.82, 32.56, 33.40, 43.73, 43.75, 113.46, 114.51, 149.04, 153.21, 162.31. Anal. calcd. for C_18_H_29_N_3_: С 75.21, Н 10.17, N 14.62. Found: С 75.03, Н 9.92, N 14.45. HRMS m/z 288.2434 [M+H]^+^ (calcd. for C_18_H_30_N_3_ 288.2432).

*N* ^1^‐(2,3,5,6,7,8‐Hexahydro‐1*H*‐cyclopenta[*b*]quinolin‐9‐yl)octane‐1,8‐diamine (**5c**): Yield 6.143 g, 78%. М.p: 83–85°C, white powder. IR (*ν*, cm^−1^): 3316, 2922, 2850 (NH, CH), 1570, 1520, 1438, 1358 (C═C, C═N). ^1^H NMR (500 MHz, CDCl_3_) *δ* = 1.22–1.26 (m, 8H, 4CH_2_), 1.31–1.34, 1.38–1.44 (both m, 4H, 2CH_2_), 1.67–1.72 (m, 4H, 2CH_2_), 1.91 (p, *J* = 7.5 Hz, 2H, CH_2_), 2.31 (t, *J* = 5.9 Hz, 2H, CH_2_), 2.53 (t, *J* = 7.0 Hz, 2H, CH_2_), 2.58 (t, *J* = 5.9 Hz, 2H, CH_2_), 2.62 (t, *J* = 7.7 Hz, 2H, CH_2_), 2.93–2.96 (m, 2Н, СН_2_), 3.25–3.31 (m, 2Н, СН_2_), 5.00 (t, *J* = 6.3 Hz, 1Н, NH), the signal of NH_2_ group was not observed due to deuterium exchange with solvent. ^13^C NMR (125 MHz, CDCl_3_) *δ* = 22.51, 22.58, 22.68, 23.33, 26.00, 26.15, 28.75, 28.78, 30.62, 30.79, 31.42, 32.54, 33.39, 40.78, 43.79, 113.45, 114.50, 149.05, 153.19, 162.29. Anal. calcd. for C_20_H_33_N_3_: С 76.14, Н 10.54, N 13.32. Found: С 76.18, Н 10.22, N 13.57. HRMS m/z 316.2750 [M+H]^+^ (calcd. for C_20_H_34_N_3_ 316.2747).

#### 4.1.4 General procedure for the synthesis of salicylamides **7a–c**

The amine **5а–с** (0.0010 mol) was added in portions with cooling (−10–0°C) to a solution of a mixture of salicylic acid chloride 6 (0.156 g, 0.0010 mol) and Et_3_N (0.17 mL, 0.0012 mol) in DMF (1 mL). The reaction mixture was stirred at 65°C for 4 h. After cooling, CH_2_Cl_2_ (30 mL) was added; the organic layer was washed with water (3 × 25 ml), dried over anhydrous Na_2_SO_4_, and evaporated. The resulting precipitate was washed with EtOH (5 mL) and dried.

*N*‐{4‐[(2,3,5,6,7,8‐Hexahydro‐1*H*‐cyclopenta[*b*]quinolin‐9‐yl)amino]butyl}−2‐hydroxybenzamide (**7a**): Yield 0.233 g, 62%. М.p: 192–194°C, white powder. IR (*ν*, cm^−1^): 3226, 2997, 2853 (NH, CH, OH), 1647 (C═O), 1586, 1567, 1536, 1452, 1431, 1365 (C═C, C═N). ^1^H NMR (500 MHz, CDCl_3_) *δ* = 164–1.67, 1.70–1.75 (both m, 4H, 2CH_2_), 1.76–1.84 (m, 4H, 2CH_2_), 2.01 (p, *J* = 7.5 Hz, 2H, CH_2_), 2.36 (t, *J* = 6.1 Hz, 2H, CH_2_), 2.80 (t, *J* = 6.1 Hz, 2H, CH_2_), 2.84 (t, *J* = 7.8 Hz, 2H, CH_2_), 3.02 (t, *J* = 7.3 Hz, 2H, CH_2_), 3.46–3.52 (m, 4H, 2CH_2_), 3.84 (t, *J* = 6.0 Hz, 1H, NH_amiridine_), 6.63 (br. s, 1H, NH_amide_), 6.82 (td, *J* = 7.6, 1.1 Hz, 1H, H_arom_), 6.98 (dd, *J* = 8.3, 1.0 Hz, 1H, H_arom_), 7.34 (dd, *J* = 8.0, 1.5 Hz, 1H, H_arom_), 7.38 (td, *J* = 7.8, 1.6 Hz, 1H, H_arom_), the signal of OH group was not observed due to deuterium exchange with solvent. ^13^C NMR (125 MHz, CDCl_3_) *δ* = 22.73, 22.98, 23.10, 23.57, 26.82, 28.58, 31.21, 32.78, 34.05, 39.28, 44.54, 113.94, 114.51, 116.03, 118.59, 118.63, 125.50, 134.15, 149.35, 154.38, 161.47, 163.52, 169.9. Anal. calcd. for C_23_H_29_N_3_O_2_: С 72.79, Н 7.70, N 11.07. Found: С 72.59, Н 7.72, N 10.89. HRMS *m/z* 380.2336 [M+H]^+^ (calcd. for C_23_H_30_N_3_O_2_ 380.2333).

*N*‐{6‐[(2,3,5,6,7,8‐Hexahydro‐1*H*‐cyclopenta[*b*]quinolin‐9‐yl)amino]hexyl}−2‐hydroxybenzamide (**7b**): Yield 0.260 g, 64%. М.p: 210–212°C, white powder. IR (*ν*, cm^−1^): 3340, 2938, 2855 (NH, CH, OH), 1640 (C═O), 1587, 1539, 1455, 1431, 1366 (C═C, C═N). ^1^H NMR (500 MHz, CDCl_3_) *δ* = 1.41–1.45, 1.55–1.66, 1.76–1.88 (all m, 12H, 6CH_2_), 2.04 (p, *J* = 7.5 Hz, 2H, CH_2_), 2.35 (t, *J* = 5.9 Hz, 2H, CH_2_), 2.83 (t, *J* = 6.0 Hz, 2H, CH_2_), 2.89 (t, *J* = 7.7 Hz, 2H, CH_2_), 3.05 (t, *J* = 7.2 Hz, 2H, CH_2_), 3.41–3.49 (m, 4H, 2CH_2_), 3.89 (br. s, 1H, NH_amiridine_), 6.38 (br. s, 1H, NH_amide_), 6.84 (td, *J* = 7.9, 0.7 Hz, 1H, H_arom_), 6.99 (d, *J* = 8.4 Hz, 1H, H_arom_), 7.35 (dd, *J* = 8.0, 1.6 Hz, 1H, H_arom_), 7.39 (td, *J* = 8.0, 1.4 Hz, 1H, H_arom_), the signal of the OH group was not observed due to deuterium exchange with solvent. ^13^C NMR (125 MHz, CDCl_3_) *δ* = 22.60, 22.92, 23.12, 23.47, 26.35, 26.61, 29.50, 29.69, 31.11, 31.22, 33.82, 39.45, 44.91, 113.85, 114.38, 116.04, 118.57, 118.72, 125.18, 134.17, 149.95, 153.67, 161.60, 166.39, 169.97. Anal. calcd. for C_25_H_33_N_3_O_2_: С 73.68, Н 8.16, N 10.31. Found: С 73.37, Н 8.22, N 10.15. HRMS *m/z* 408.2649 [M+H]^+^ (calcd. for C_25_H_34_N_3_O_2_ 408.2646).

*N*‐{8‐[(2,3,5,6,7,8‐hexahydro‐1*H*‐cyclopenta[*b*]quinolin‐9‐yl)amino]octyl}−2‐hydroxybenzamide (**7c**): Yield 0.261 g, 60%. М.p: 148–150°C, white powder. IR (*ν*, cm^−1^): 3342, 2923, 2852 (NH, CH, OH), 1647 (C═O), 1583, 1553, 1526, 1451, 1430, 1365 (C═C, C═N). ^1^H NMR (500 MHz, CDCl_3_) *δ* = 1.31–1.38 (m, 8H, 4CH_2_), 1.52–1.62, 1.78–1.84 (both m, 8H, 4CH_2_), 2.03 (p, *J* = 7.5 Hz, 2H, CH_2_), 2.35 (t, *J* = 5.3 Hz, 2H, CH_2_), 2.81 (t, *J* = 5.5 Hz, 2H, CH_2_), 2.85 (t, *J* = 7.7 Hz, 2H, CH_2_), 3.05 (t, *J* = 7.1 Hz, 2H, CH_2_), 3.39–3.45 (m, 4H, 2CH_2_), 3.84 (unsolv. t, 1H, NH_amiridine_), 6.79 (br. s, 1H, NH_amide_), 6.83 (t, *J* = 7.5 Hz, 1H, H_arom_), 6.97 (d, *J* = 8.2 Hz, 1H, H_arom_), 7.36 (t, *J* = 7.4 Hz, 1H, H_arom_), 7.46 (d, *J* = 7.9 Hz, 1H, H_arom_), the signal of OH group was not observed due to deuterium exchange with solvent. ^13^C NMR (125 MHz, CDCl_3_) *δ* = 22.65, 22.93, 23.12, 23.44, 26.58, 26.78, 29.10, 29.14, 29.45, 31.14, 31.17, 32.46, 33.85, 39.57, 44.96, 113.76, 114.93, 115.97, 118.46, 118.48, 125.87, 133.86, 149.82, 153.86, 161.30, 163.09, 169.66. Anal. calcd. for C_27_H_37_N_3_O_2_: С 74.45, Н 8.56, N 9.65. Found: С 74.26, Н 8.82, N 9.40. HRMS *m/z* 436.2957 [M+H]^+^ (calcd. for C_27_H_38_N_3_O_2_ 436.2959).

#### 4.1.5 General procedure for the synthesis of salicylimines **9а–с**

A mixture of a solution of amine **5a–c** (0.001 mol) in EtOH (2 mL) and a solution of salicylic aldehyde **8** (0.10 mL, 0.001 mol) in toluene (50 mL) was refluxed with azeotropic distillation of water for 6 h. Then the solvent was distilled off on a rotary evaporator, hexane was added to the residue, the resulting precipitate was filtered and dried.

2‐[({4‐[(2,3,5,6,7,8‐Hexahydro‐1*H*‐cyclopenta[*b*]quinolin‐9‐yl)amino]butyl}imino)methyl]phenol (**9a**): Yield 0.309 g, 85%. М.p: 152–153°C, yellow powder. IR (*ν*, cm^−1^): 3410, 2924, 2856 (NH, CH, OH), 1631 (N═C_imine_), 1569, 1506, 1438, 1369, 1354 (C═C, C═N). ^1^H NMR (500 MHz, CDCl_3_) *δ* 1.62–1.69 (m, 2H, CH_2_), 1.76–1.83 (m, 6H, 3CH_2_), 2.02 (p, *J* = 7.5 Hz, 2H, CH_2_), 2.35 (t, *J* = 5.8 Hz, 2H, CH_2_), 2.80 (t, *J* = 5.7 Hz, 2H, CH_2_), 2.85 (t, *J* = 7.7 Hz, 2H, CH_2_), 3.04 (t, *J* = 7.3 Hz, 2H, CH_2_), 3.44–3.49 (m, 2H, CH_2_), 3.63 (td, *J*=6.5, 0.9 Hz, 2H, N–CH_2_), 3.79 (t, *J* = 5.8 Hz, 1H, NH_amiridine_), 6.88 (td, *J* = 7.5, 1.1 Hz, 1H, H_arom_), 6.96 (d, *J* = 8.3 Hz, 1H, H_arom_), 7.25 (dd, *J* = 7.7, 1.7 Hz, 1H, H_arom_), 7.31 (td, *J* = 7.6, 1.7 Hz, 1H, H_arom_), 8.35 (s, 1H, ═CH), 13.44 (s, 1Н, OH). ^13^C NMR (125 MHz, CDCl_3_) *δ* 22.73, 22.96, 23.09, 23.50, 28.04, 28.88, 31.17, 32.91, 34.16, 44.74, 59.16, 113.73, 115.81, 116.95, 118.56, 118.65, 131.14, 132.21, 149.26, 154.35, 161.08, 163.56, 164.99. Anal. calcd. for C_23_H_29_N_3_O: С 76.00, Н 8.04, N 11.53. Found: С 75.88, Н 8.19, N 11.47. HRMS *m/z* 364.2381 [M+H]^+^ (calcd. for C_23_H_30_N_3_O 364.2382). The suitable yellow single crystals of compound **9a** were obtained by slow crystallization from the mixture of DMSO/CHCl_3_ (1:100). Main crystallographic data for **9a**: C_23_H_29_N_3_O, M 363.49, space group P2_1_/n, monoclinic, *a* 16.8017(16), *b* 4.8464(4), *c* 24.498(2) Å; *β* 105.389(10)°; *V* 1923.3 Å^3^; *Z* 4; *D*_*calc*_ 1.255 g∙cm^−3^; μ 0.078 mm^−1^; 253 refinement parameters; 13054 reflections measured, 5068 [*R*_int_ = 0.0639, *R*_sigma_ = 0.0915] unique reflections which were used in all calculations. The final *R*_1_ = 0.0781, *wR*_2_ = 0.2705 [I ≥ 2σ (I)] [all data]. CCDC 2370212 contains the supplementary crystallographic data for this compound.

2‐[({6‐[(2,3,5,6,7,8‐Hexahydro‐1*H*‐cyclopenta[*b*]quinolin‐9‐yl)amino]hexyl}imino)methyl]phenol (**9b**): Yield 0.239 g, 61%. М.p: 123–125°C, yellow powder, IR (*ν*, cm^−1^): 3415, 2934, 2855 (NH, CH, OH), 1631 (N═C_imine_), 1572, 1506, 1439, 1368, 1320 (C═C, C═N). ^1^H NMR (500 MHz, CDCl_3_) *δ* 1.42–1.43 (m, 4H, 2CH_2_), 1.54–1.60, 1.69–1.74 (both m, 4H, 2CH_2_), 1.77–1.86 (m, 4H, CH_2_), 2.03 (p, *J* = 7.5 Hz, 2H, CH_2_), 2.35 (t, *J* = 6.0 Hz, 2H, CH_2_), 2.82 (t, *J* = 5.9 Hz, 2H, CH_2_), 2.87 (t, *J* = 5.9 Hz, 2H, CH_2_), 3.04 (t, *J* = 7.2 Hz, 2H, CH_2_), 3.40–3.44 (m, 2H, CH_2_), 3.59 (td, *J* = 6.7, 0.6 Hz, 2H, N–CH_2_), 3.83 (unsolv. t, 1H, NH_amiridine_), 6.87 (td, *J* = 7.5, 0.9 Hz, 1H, H_arom_), 6.95 (d, *J* = 8.2 Hz, 1H, H_arom_), 7.24 (dd, *J* = 7.6, 1.6 Hz, 1H, H_arom_), 7.30 (td, *J* = 7.7, 1.6 Hz, 1H, H_arom_), 8.33 (s, 1H, ═CH), 13.61 (s, 1H, OH). ^13^C NMR (125 MHz, CDCl_3_) *δ* 22.67, 22.95, 23.11, 23.46, 26.48, 26.91, 30.68, 31.10, 31.18, 32.64, 34.01, 44.95, 59.35, 113.72, 115.91, 116.98, 118.46, 118.74, 131.05, 132.10, 149.65, 153.95, 161.24, 163.18, 164.60. Anal. calcd. for C_25_H_33_N_3_O: С 76.69, Н 8.50, N 10.73. Found: С 76.48, Н 8.38, N 10.46. HRMS *m/z* 392.2695 [M+H]^+^ (calcd. for C_25_H_34_N_3_O 392.2696).

2‐[({8‐[(2,3,5,6,7,8‐Hexahydro‐1*H*‐cyclopenta[*b*]quinolin‐9‐yl)amino]octyl}imino)methyl]phenol (**9c**): Yield 0.249 g, 59%. М.p: 93–95°C, yellow powder. IR (*ν*, cm^−1^): 3411, 2922, 2851 (NH, CH, OH), 1632 (N═C_imine_), 1571, 1506, 1439, 1368, 1315 (C═C, C═N). ^1^H NMR (500 MHz, CDCl_3_) *δ* = 1.34–1.39 (m, 8H, 4CH_2_), 1.51–1.55, 1.66–1.72 (both m, 4H, 2CH_2_), 1.77–1.86 (m, 4H, 2CH_2_), 2.03 (p, *J* = 7.5 Hz, 2H, CH_2_), 2.35 (t, *J* = 6.1 Hz, 2H, CH_2_), 2.81 (t, *J* = 6.0 Hz, 2H, CH_2_), 2.86 (t, *J* = 7.7 Hz, 2H, CH_2_), 3.05 (t, *J* = 7.3 Hz, 2H, CH_2_), 3.38–3.42 (m, 2H, CH_2_), 3.59 (td, *J* = 6.7, 0.9 Hz, 2H, N–CH_2_), 3.79 (unsolv. t, 1H, NH_amiridine_), 6.87 (td, *J* = 7.5, 1.0 Hz, 1H, H_arom_), 6.95 (dd, *J* = 8.3, 0.3 Hz, 1H, H_arom_), 7.24 (dd, *J* = 7.6, 1.6 Hz, 1H, H_arom_), 7.30 (td, *J* = 6.4, 1.7 Hz, 1H, H_arom_), 8.33 (s, 1H, ═CH), 13.67 (s, 1Н, ОН). ^13^C NMR (125 MHz, CDCl_3_) *δ* = 22.75, 23.01, 23.12, 23.48, 26.67, 27.04, 29.22, 30.78, 31.16, 31.19, 32.82, 34.14, 45.05, 59.46, 113.67, 115.87, 117.00, 118.39, 118.78, 131.02, 132.03, 149.57, 154.15, 161.32, 163.38, 164.46. Anal. calcd. for C_27_H_37_N_3_O: С 77.28, Н 8.89, N 10.01. Found: С 76.99, Н 8.89, N 9.82. HRMS *m/z* 420.3009 [M+H]^+^ (calcd. for C_27_H_38_N_3_O 420.3009). The suitable yellow single crystals of compound **9с** were obtained by slow crystallization from the mixture of DMSO/CHCl_3_ (1:100). Main crystallographic data for **9с**: C_27_H_37_N_3_O, M 419.59, space group P2_1_/c, monoclinic, *a* 21.958(2), *b* 4.7500(7), *c* 25.943(3) Å; *β* 111.276(13)°; *V* 2521.5(6) Å^3^; *Z* 4; *D*_calc_ 1.105 g∙cm^−3^; μ 0.067 mm^−1^; 288 refinement parameters; 9252 reflections measured, 5107 [*R*_int_ = 0.0544, *R*_sigma_ = 0.1304] unique reflections which were used in all calculations. The final *R*_1_ = 0.0684, *wR*_2_ = 0.1943 [I ≥ 2σ (I)] [all data]. CCDC 2370211 contains the supplementary crystallographic data for this compound.

#### 4.1.6 General procedure for the synthesis of salicylamines **10а–с**

NaBH_4_ (0.076 g, 0.002 mol) was added to a solution of compounds **9a–c** (0.0005 mol) in ethanol (20 mL). The reaction mixture was stirred at room temperature for 2 h and evaporated. CH_2_Cl_2_ (25 mL) was added to the residue, the solution was filtered from NaBH_4_, the filtrate was evaporated and dried.

2‐[({4‐[(2,3,5,6,7,8‐Hexahydro‐1*H*‐cyclopenta[*b*]quinolin‐9‐yl)amino]butyl}amino)methyl]phenol (**10a**): Yield 0.168 g, 92%. М.p: 123–124°C, white powder. IR (*ν*, cm^−1^): 3234, 2922, 2897, 2855 (NH, CH, OH), 1576, 1540, 1456, 1436, 1367 (C═C, C═N). ^1^H NMR (500 MHz, CDCl_3_) *δ* = 1.61–1.63, 1.77–1.86 (both m, 8H, 4CH_2_), 2.04 (p, *J* = 7.5 Hz, 2H, CH_2_), 2.33–2.35 (m, 2H, CH_2_), 2.70–2.74 (m, 2H, CH_2_), 2.83 (t, *J* = 5.9 Hz, 2H, CH_2_), 2.89 (t, *J* = 7.7 Hz, 2H, CH_2_), 3.03 (t, *J* = 7.2 Hz, CH_2_), 3.43–3.47 (m, 2H, CH_2_), 3.90 (br. s, 1H, NH_amiridine_), 4.00 (s, 2H, NHCH_2_Ar), 6.78 (td, *J* = 7.4, 1.1 Hz, 1H, H_arom_), 6.82 (dd, *J* = 8.1, 0.8 Hz, 1H, H_arom_), 6.98 (dd, *J* = .4, 0.8 Hz, 1H, H_arom_), 7.17 (td, *J* = 8.0, 1.6 Hz, 1H, H_arom_), 11.09 (br. s, 1Н, NH), the signal of OH group was not observed due to deuterium exchange with solvent. ^13^C NMR (125 MHz, CDCl_3_) *δ* = 22.49, 22.83, 23.08, 23.42, 26.81, 28.79, 31.18, 32.19, 33.68, 44.65, 48.26, 52.71, 113.90, 115.98, 116.31, 119.04, 122.36, 128.27, 128.74, 149.84, 153.47, 158.12, 162.68. Anal. calcd. for C_23_H_31_N_3_O: С 75.40, Н 8.78, N 11.35. Found: С 75.58, Н 8.55, N 11.50. HRMS *m/z* 366.2539 [M+H]^+^ (calcd. for C_23_H_32_N_3_O 366.2540).

2‐[({6‐[(2,3,5,6,7,8‐Hexahydro‐1*H*‐cyclopenta[*b*]quinolin‐9‐yl)amino]hexyl}amino)methyl]phenol (**10b**): Yield 0.175, 89%. Сolorless oil. IR (*ν*, cm^−1^): 3234, 2926, 2854 (NH, CH, OH), 1573, 1510, 1454, 1364 (C═C, C═N). ^1^H NMR (500 MHz, CDCl_3_) *δ* = 1.36–1.40, 1.51–1.59, 1.76–1.87 (all m, 12H, 6CH_2_), 2.03 (p, *J* = 7.5 Hz, 2H, CH_2_), 2.35 (t, *J* = 5.6 Hz, 2H, CH_2_), 2.68 (t, *J* = 7.0 Hz, 2H, CH_2_), 2.81 (t, *J* = 5.8 Hz, 2H, CH_2_), 2.86 (t, *J* = 7.8 Hz, 2H, CH_2_), 3.04 (t, *J* = 7.2 Hz, 2H, CH_2_), 3.38–3.43 (m, 2H, CH_2_), 3.80 (t, *J* = 4.8 Hz, 1H, NH_amiridine_), 3.99 (s, 2H, NHCH_2_Ar), 6.77 (td, *J* = 7.4, 1.0 Hz, H_arom_), 6.82 (dd, *J* = 8.1, 0.8 Hz, H_arom_), 6.99 (dd, *J* = 7.3, 0.5 Hz, 1H, H_arom_), 7.16 (td, *J* = 8.0, 1.5 Hz, 1H, H_arom_), the signals of NH and OH groups were not observed due to deuterium exchange with solvent. ^13^C NMR (125 MHz, CDCl_3_) *δ* = 22.53, 22.86, 23.09, 23.40, 26.51, 26.83, 29.48, 31.10, 31.18, 32.24, 33.74, 44.88, 48.52, 52.75, 113.76, 115.93, 116.29, 118.92, 122.49, 128.19, 128.64, 149.92, 153.48, 158.26, 162.72. Anal. calcd. for C_25_H_35_N_3_O: С 76.29, Н 8.96, N 10.68. Found: С 76.01, Н 9.15, N 10.48. HRMS *m/z* 394.2856 [M+H]^+^ (calcd. for C_25_H_36_N_3_O 394.2853).

2‐[({8‐[(2,3,5,6,7,8‐Hexahydro‐1*H*‐cyclopenta[*b*]quinolin‐9‐yl)amino]octyl}amino)methyl]phenol (**10c**): Yield 0.196 g, 93%. Colorless oil. IR (*ν*, cm^−1^): 3227, 2924, 2853 (NH, CH, OH), 1573, 1491, 1455, 1366 (C═C, C═N). ^1^H NMR (500 MHz, CDCl_3_) *δ* = 1.29–1.35 (m, 8H, 4CH_2_), 1.50–1.57, 1.77–1.86 (both m, 8H, 4CH_2_), 2.03 (p, *J* = 7.5 Hz, 2H, CH_2_), 2.35 (t, *J* = 6.0 Hz, 2H, CH_2_), 2.67 (t, *J* = 7.1 Hz, 2H, CH_2_), 2.81 (t, *J* = 6.0 Hz, 2H, CH_2_), 2.87 (t, *J*=7.7 Hz, 2H, CH_2_), 3.05 (t, *J* = 7.2 Hz, 2H, CH_2_), 3.39–3.43 (m, 2H, CH_2_), 3.80 (br. s, 1H, NH_amiridine_), 3.99 (s, 2H, NHCH_2_Ar), 6.77 (td, *J* = 8.1, 0.8 Hz, 1H, H_arom_), 6.98 (dd, *J =* 7.3 Hz, 1H, H_arom_), 7.16 (td, *J* = 7.7, 1.5 Hz, 1H, H_arom_), 11.57 (br. s, 1Н, NH), the signal of OH group was not observed due to deuterium exchange with solvent. ^13^C NMR (125 MHz, CDCl_3_) *δ* = 22.68, 22.96, 23.12, 23.45, 26.65, 27.00, 29.21, 29.29, 29.55, 31.14, 31.19, 32.62, 34.00, 45.03, 48.69, 52.77, 113.69, 115.90, 116.31, 118.89, 122.57, 128.17, 128.62, 149.71, 153.93, 158.32, 163.18. Anal. calcd. for C_27_H_39_N_3_O: С 76.92, Н 9.32, N 9.97. Found: С 76.64, Н 9.58, N 9.88. HRMS *m/z* 422.3170 [M+H]^+^ (calcd. for C_27_H_40_N_3_O 422.3166).

#### 4.1.7 Synthesis of *N*‐hexyl‐2,3,5,6,7,8‐hexahydro‐1*H*‐cyclopenta[*b*]quinolin‐9‐amine (**11**)

A mixture of 9‐chloro‐2,3,5,6,7,8‐hexahydro‐1*H*‐cyclopenta[*b*]quinoline 3 (0.21 g, 0.0010 mol), hexylamine (0.14 g, 0.0014 mol), and KI (0.00002 mol) in pentanol‐1 (20 mL) was stirred in a closed vessel at 180–190°C for 16 h in an argon atmosphere. Then the solvent and diamine were distilled off, chloroform (70 mL) was added to the reaction mixture, the organic layer was washed with a 10% NaOH solution (3 × 50 mL), water (3 × 50 mL), dried over anhydrous Na_2_SO_4_ and evaporated. The residue was purified by column chromatography (eluent CHCl_3_/EtOH/NH_4_OH 10:1:0.1 → 5:1:0.1). Yield 0.258 g, 82%. М.p: 81–82°C, white powder. IR (*ν*, cm^−1^): 3265, 2924, 2852 (NH, CH), 1568, 1518, 1439, 1361 (C═C, C═N). ^1^H NMR (500 MHz, CDCl_3_) *δ*=0.88–0.90 (m, 3H, CH_3_), 1.27–1.39 (m, 6H, 3CH_2_), 1.51–1.57 (m, 2H, CH_2_), 1.77–1.86 (m, 4H, 2CH_2_), 2.03 (t, *J* = 6.0 Hz, 2H, CH_2_), 2.34–2.36 (m, 2H, CH_2_), 2.79–2.81 (m, 2H, CH_2_), 2.85 (t, *J* = 7.7 Hz, 2H, CH_2_), 3.05 (t, *J* = 7.3 Hz, 2H, CH_2_), 3.40 (dd, *J* = 13.3, 6.8 Hz, 2H, CH_2_), 3.76 (unsolv. t, 1H, NH). ^13^C NMR (125 MHz, CDCl_3_) *δ* = 13.95, 22.56, 22.82, 23.06, 23.14, 23.52, 26.43, 13.17, 31.19, 31.53, 32.97, 34.24, 45.14, 113.66, 115.90, 149.52, 154.34, 163.55. Anal. calcd. for C_18_H_28_N_2_: С 79.36, Н 10.36, N 10.28. Found: С 79.13, Н 10.55, N 9.99. HRMS *m/z* 273.2329 [M+H]^+^ (calcd. for C_18_H_29_N_2_ 273.2325).

#### 4.1.8 Synthesis of 2‐[(hexylimino)methyl]phenol (**12**)

A mixture of salicylic aldehyde (0.50 g, 0.0041 mmol) and hexylamine (0.41 g, 0.0041 mmol) in EtOH (25 mL) was stirred at room temperature for 30 min. The reaction mixture was evaporated and dried. The physicochemical properties of the compound **12** coincided with the literature data.^[^ ^63^ ^]^ Yield 0.823 g, 98%. IR (*ν*, cm^−1^): 2955, 2929, 2856 (NH, CH), 1632 (C═N), 1582, 1496, 1461, 1415 (C═C, C–H).

#### 4.1.9 Synthesis of 2‐[(hexylamino)methyl]phenol (**13**)

NaBH_4_ (0.076 g, 0.002 mol) was added to a solution of compound **12** (0.0005 mol) in ethanol (20 mL). The reaction mixture was stirred at room temperature for 2 h and evaporated. CH_2_Cl_2_ (25 mL) was added to the residue, the solution was filtered from NaBH_4_, the filtrate was evaporated, and dried. Yield 0.099 g, 95%. Light yellow liquid. IR (*ν*, cm^−1^): 2955, 2927, 2856 (NH, CH), 1589, 1488, 1466, 1410 (C═C, C–H). ^1^H NMR (500 MHz, CDCl_3_) *δ* = 0.87–0.90 (m, 3H, CH_3_), 1.29–1.32 (m, 6H, 3CH_2_), 1.50–1.56 (m, 2H, CH_2_), 2.67 (t, 2H, CH_2_), 3.99 (s, 2H, Ar–CH_2_–NH); 6.76 (td, *J* = 7.4, 1.1 Hz, 1H, H_arom_), 6.82 (dd, *J* = 8.1, 0.9 Hz, 1H, H_arom_), 6.98 (unsolv. dd, *J* = 7.4 Hz, 1H, H_arom_), 7.16 (td, *J* = 8.0, 1.6 Hz, 1H, H_arom_) the signals of OH and NH groups were not observed due to deuterium exchange with solvent. ^13^C NMR (125 MHz, CDCl_3_) *δ* = 13.99, 22.54, 26.79, 29.53, 31.61, 48.77, 52.75, 116.34, 118.86, 122.61, 128.16, 128.60, 158.38. Anal. calcd. for C_13_H_21_NO: С 75.32, Н 10.21, N 6.76. Found: С 75.06, Н 10.12, N 6.51.

### 4.2 Biological assays

#### 4.2.1 Enzymatic assays

##### In Vitro AChE, BChE, and CES inhibition

All experiments were conducted in accordance with the standard protocols approved by IPAС Russian Academy of Sciences (RAS).

The following items were purchased from Sigma‐Aldrich: human erythrocyte AChE, equine serum BChE, porcine liver CES, acetylthiocholine iodide (ATCh), butyrylthiocholine iodide (BTCh), 5,5′‐dithio‐bis‐(2‐nitrobenzoic acid) (DTNB), 4‐nitrophenyl acetate (4‐NPA), tacrine, and BNPP. We measured the activity of AChE and BChE according to the colorimetric Ellman procedure (λ = 412 nm), as described in detail in Makhaeva et al.^[^ ^64^ ^]^ CES activity was assessed as described in Makhaeva et al.^[^ ^64^ ^]^ by following the release of 4‐nitrophenol spectrophotometrically (λ = 405 nm) using 4‐NPA as a substrate. Freshly prepared solutions of the enzymes were used, which retained a constant activity during the experiment (2–2.5 h). Chromophores absorbance was measured with a SPECTROStar Nano microplate reader (BMG Labtech). DMSO (2% v/v) was employed as the solvent; the concentration used did not alter the activities of the enzymes (data not shown). Initially, we used a single concentration of 20 µM for all compounds. Subsequently, IC_50_ values (µM) were determined for the most active compounds against AChE, BChE, and CES.

##### Kinetic study of AChE and BChE inhibition. Determination of steady‐state inhibition constants

We assessed the mechanisms of AChE and BChE inhibition by performing a thorough analysis of enzyme kinetics. After a 5 min incubation at 25°C (for temperature equilibration) with three increasing concentrations of inhibitor and six decreasing substrate concentrations, the residual enzyme activity was measured as described above for enzymatic assays. Linear regression of 1/V versus 1/[S] double‐reciprocal (Lineweaver–Burk) plots were used to determine the inhibition constants for the competitive component (*K*_i_) and noncompetitive component (α*K*_i_).

#### 4.2.2 Propidium displacement studies

The ability of the test compounds to competitively displace propidium was evaluated by the fluorescence method,^[^ ^65^ ^]^ as described in detail in Makhaeva et al.^[^ ^66^ ^]^ Propidium iodide, donepezil, and electric eel AChE (*Ee*AChE, type VI‐S, lyophilized powder) were purchased from Sigma‐Aldrich. After 15 min of incubation of the test compounds at a concentration of 20 μM with a 7 μM solution of *Ee*AChE in 1 mM Tris‐HCl buffer, pH 8.0, 25°C, propidium iodide (final concentration 8 μM) was added. Then, the solutions were incubated for 15 min and the fluorescence spectrum was recorded (530 nm (excitation) and 600 nm (emission)). Donepezil and amiridine were the reference compounds. Measurements were performed in triplicate on a FLUOStar Optima microplate reader (BMG LabTech).

#### 4.2.3 Inhibition of β‐amyloid (1–42) (Aβ_42_) self‐aggregation

Inhibition of Aβ_42_ self‐aggregation by test compounds was studied using the ThT fluorescence method^[^ ^67^, ^68^ ^]^ with minor modifications as described in detail in Makhaeva et al.^[^ ^16^ ^]^ Lyophilized hexamfluoroisopropanol‐pretreated Aβ_42_ (GenicBio Limited, 1 mg) was dissolved in DMSO to obtain a stable 500 μM solution. The samples of 50 μM Aβ_42_ in 215 mM Na‐phosphate buffer pH 8.0 were incubated for 24 h at 37°C in the absence or presence of 100 μM test compounds. Myricetin and propidium iodide in the same concentration were used as references. After incubation, 5 μM ThT in 50 mM glycine‐NaOH buffer pH 8.5 was added, and the fluorescence was measured at 440 nm (exc.) and 485 nm (emis.) with a FLUOStar Optima microplate reader (LabTech). Blanks consisted of 215 mM Na‐phosphate buffer, pH 8.0, 20% (v/v) DMSO, or test compounds, respectively. Each assay was run in triplicate.

#### 4.2.4 AOA

##### ABTS radical cation scavenging activity assay

Radical scavenging activity of the compounds was evaluated using the ABTS radical cation (2,2′‐azinobis‐(3‐ethylbenzothiazoline‐6‐sulfonic acid, ABTS^•+^) decolorization assay.^[^ ^69^, ^70^ ^]^ The solution of cation radical ABTS^•+^ was produced by incubation of an aqueous solutions of 7 mM ABTS and 2.45 mM potassium persulfate in equal quantities for 12–16 h at room temperature in the dark. Radical scavenging capacity of the compounds was analyzed by mixing 10 μL of compound solution in DMSO with 240 μL of ABTS^•+^ working solution in ethanol (100 μM final concentration). The reduction of ABTS^•+^ absorbance was measured spectrophotometrically at 734 nm using a xMark UV/VIS microplate spectrophotometer (Bio‐Rad) for 1 h with an interval of 1–10 min compared to a standard synthetic antioxidant, Trolox. Data are given for 1 h of incubation of compounds with ABTS^•+^. The AOA of the compounds was evaluated as Trolox equivalent antioxidant capacity (TEAC values) as the ratio of the slopes of the concentration–response curves, test compound/Trolox. All tests were performed at least in triplicate in three independent experiments. The IC_50_ values for the test compounds were also determined.

##### FRAP assay

The FRAP assay proposed by Benzie and Strain^[^ ^71^ ^]^ and modified to be performed in 96‐well microplates^[^ ^66^ ^]^ was used. A total of 10 μL (0.5 mM) of the tested compound or reference compound was mixed with 240 μL of the FRAP reagent, and the absorbance of the mixture was measured spectrophotometrically (λ = 593 nm) with a SPECTROStar Nano microplate reader (BMG LabTech) at 593 nm, after a 1 h incubation at 37°C against a blank. Trolox was used as a reference compound. The AOA was calculated and expressed as Trolox equivalents (TE)—the values calculated as the ratio of the concentrations of Trolox and the test compound resulting in the same effect on ferric‐reducing activity.

### 4.3 Statistical analyses

Results for Aβ_42_ self‐aggregation, AOA, enzyme kinetics, esterase assays, and propidium displacement are presented as mean ± SEM calculated using GraphPad Prism version 6.05 for Windows from at least three technical replicates per experiment and three independent experiments. Plots, linear regressions, and IC_50_ values for these results were determined using Origin 6.1 for Windows, OriginLab. Cytotoxicity IC_50_ values are expressed as mean ± SEM calculated using GraphPad Prism version 7.00 for Windows from three technical replicates drawn from each of six experiments (two experiments for each of the three cell types with the six test compounds and the amiridine reference compound split between each experiment).

### 4.4 Molecular modeling studies

#### 4.4.1 Molecular docking

All compounds were optimized by density functional theory (DFT) quantum‐chemical methods using the B3LYP functional, 6‐31 G* basis set and D3 version of Grimme's dispersion correction. The Mulliken charges from the quantum chemical calculations were assigned to compounds for MD simulations. Molecular docking was performed with AMDock 1.5.2.^[^ ^72^ ^]^ The PDB structures of human cholinesterases: AChE (PDB: 4EY7^[^ ^73^ ^]^ and BChE (PDB: 1P0I^[^ ^74^, ^75^ ^]^) were used for molecular docking. All 10 conformers of Aβ_42_ available in the PDB: 1IYT^[^ ^44^, ^76^ ^]^ structure were used for molecular docking.

For docking with AChE, different grid boxes were chosen: the first included the entire active site gorge of AChE (26 Å × 26 Å × 26 Å; center *x* = –14.4 Å, *y* = –42.9 Å, *z* = 2.2 Å), the second box corresponded to the CAS (21 Å × 21 Å × 21 Å; center *x* = –3.0Å *y* = –40.0 Å *z* = 31.0 Å; was used for model compounds M_1_–M_5_), the third option corresponded to the residues of the PAS (24 Å × 24 Å × 24 Å; *x* =–17 Å *y*=–42 Å *z*=29 Å; was used in M_1_–M_5_ docking). For BChE, the grid box dimensions were 15 Å × 21 Å × 18 Å with center *x* = 137.0 Å, *y* = 117.0 Å, *z* = 44.0 Å. For Aβ_42_ structures, a 44 Å × 36 Å × 55 Å grid was used; the grid center corresponded to *x* = −3.2 Å, *y* = –0.1 Å, *z* = 1.3 Å. A grid spacing of 0.375 Å was used in all molecular docking experiments.

#### 4.4.2 Quantum chemical analysis of AOA

The antiradical activity of the studied compounds was assessed on the basis of their energy characteristics calculated by the DFT quantum mechanical modeling method. The calculations were performed with Gaussian 16^[^ ^77^ ^]^ and Priroda 20^[^ ^78^ ^]^ packages. The preliminary conformation search was carried out in the Priroda package using the PBE0 functional^[^ ^79^ ^]^ and def2‐SVP basis set^[^ ^80^ ^]^ in the gas phase. The final optimization was carried out in the Gaussian package using the B3LYP functional^[^ ^81^ ^]^ with the empirical Grimme correction^[^ ^82^ ^]^ and 6‐31++g(d,p) basis set.^[^ ^83^ ^]^ All stationary states were checked by the absence of negative frequencies in the vibrational spectrum. The solvent was taken into account in the continuum solvent model (SMD).^[^ ^84^ ^]^

#### 4.4.3 Prediction of ADMET, physicochemical, and PAINS profiles

Lipophilicity (LogP_ow_) and aqueous solubility (pS) were estimated by the ALogPS 3.0 neural network model implemented in the OCHEM platform.^[^ ^85^ ^]^ Human intestinal absorption (HIA),^[^ ^86^ ^]^ blood–brain barrier distribution/permeability (LogBB),^[^ ^87^, ^88^ ^]^ and hERG‐mediated cardiac toxicity risk (channel affinity p*K*_*i*_ and inhibitory activity pIC_50_)^[^ ^89^ ^]^ were estimated using the integrated online service for the prediction of ADMET properties.^[^ ^90^ ^]^ This service implements predictive quantitative structure‐activity relationship models based on accurate and representative training sets, fragmental descriptors, and artificial neural networks. The quantitative estimate of drug‐likeness (QED) values^[^ ^91^ ^]^ were calculated and the PAINS alerts were checked using RDKit version 2021.09.2 software.^[^ ^92^ ^]^

### 4.5 Cytotoxicity studies

#### 4.5.1 Cell lines

HepG2 cells (human hepatocellular carcinoma) were obtained from the Russian collection of cell cultures of vertebrates (Saint‐Petersburg, Russia). The tumor cells were grown in Eagle's minimum essential medium (PanEco). HEK293T (human embryonic kidney) and SH‐SY5Y (human neuroblastoma) cells were purchased from the American Type Culture Collection (ATCC). HEK293T cells were cultured in a Dulbecco's modified Eagle medium (DMEM) medium (PanEco) and SH‐SY5Y cells were cultivated in a modified DMEM/F‐12 medium (Gibco). Аll types of cell lines were cultured with the addition of 10% (v/v) fetal bovine serum (FBS, HyClone), 2 μM glutamine, penicillin (50 U/mL) and streptomycin (50 mg/mL) at 37°C in atmospheric humidity and 5% (v/v) CO_2_.

#### 4.5.2 Cytotoxicity

The determination of the cytotoxic effect of tested compounds using MTT was carried out in accordance with the methods described earlier.^[^ ^53^, ^54^, ^55^ ^]^

Cells were seeded in 96‐well plates at 15 × 10^3^/100 μL cells per well. At 24 h after plating, the test compounds that were previously dissolved in DMSO (Molekula Ltd.) were added to the culture medium. The final concentration of compounds in the incubation medium ranged from 0.3 to 100 μM. Then the cells were incubated under the same conditions for 24 h. For each concentration, the experiments were performed in triplicate. The final concentration of DMSO was 1% (v/v), a concentration determined not to produce cytotoxicity. The solvent was added to the control wells at a final concentration of 1% (v/v). After 24 h cells were stained with MTT (neoFroxx Ltd., 1334GR005) at a concentration of 0.5 mg/mL for 2 h, then the medium was aspirated, and MTT formazan was dissolved in 100 μL of DMSO. The staining intensity was measured at 536 nm on a Cytation 3 plate reader (BioTek). Cell viability was determined as a percentage of MTT staining of control cells. IC_50_ concentrations were determined from concentration‐response curves using GraphPad Prism version 7.00 (GraphPad Software).

## CONFLICTS OF INTEREST STATEMENT

R.J.R. is a member of the advisory board of NeuroXI, a computational neuroscience company for the discovery and development of therapeutics for neurodegenerative disorders. The remaining authors declare no conflict of interest.

## Supporting information

Supporting information.

Supporting information.

## Data Availability

The data that support the findings of this study are available in the supplementary material of this article.
