# Supplementary material for: Conjugates of amiridine and salicylic derivatives as promising multifunctional CNS agents for potential treatment of Alzheimer's disease
Source: Arch Pharm (Weinheim). 2024 Dec 17;358(1):e2400819. doi: 10.1002/ardp.202400819 (PMC11650361; doi:10.1002/ardp.202400819)
Supplement: Supplementary file 1 — Supporting information. [file ARDP-358-e2400819-s001.docx]

**Supporting Information**

**Conjugates of Amiridine and Salicylic Acid Derivatives as promising multifunctional CNS agents for potential treatment of Alzheimer's disease**

Galina F. Makhaeva, Maria V. Grishchenko, Nadezhda V. Kovaleva, Natalia P. Boltneva, Elena V. Rudakova, Tatiana Y. Astakhova, Elena N. Timokhina, Pavel G. Pronkin, Sofya V. Lushchekina, Olga G. Khudina, Ekaterina F. Zhilina, Evgeny V. Shchegolkov, Maria A. Lapshina, Elena S. Dubrovskaya, Eugene V. Radchenko, Vladimir A. Palyulin, Yanina V. Burgart, Victor I. Saloutin, Valery N. Charushin, Rudy J. Richardson

**Table of Contents**

[Quantum Chemical Analyses of Antioxidant Activity (AOA) 5](#_Toc179967500)

[Figure S1. ^1^H NMR spectrum of 1,2,3,4,5,6,7,8-octahydro-9H-cyclopenta[b]quinolin-9-one (2) 7](#_Toc179967501)

[Figure S2. ^13^C NMR spectrum of 1,2,3,4,5,6,7,8-octahydro-9H-cyclopenta[b]quinolin-9-one (2) 8](#_Toc179967502)

[Figure S3. ^1^H NMR spectrum of 9-chloro-2,3,5,6,7,8-hexahydro-1*H*-cyclopenta[*b*]quinolone (3) 9](#_Toc179967503)

[Figure S4. ^13^C NMR spectrum of 9-chloro-2,3,5,6,7,8-hexahydro-1*H*-cyclopenta[*b*]quinolone (3) 10](#_Toc179967504)

[Figure S5. ^1^H NMR spectrum of *N*^1^-(2,3,5,6,7,8-hexahydro-1*H*-cyclopenta[*b*]quinolin-9-yl)butane-1,4-diamine (5a) 11](#_Toc179967505)

[Figure S6. ^13^C NMR spectrum of *N*^1^-(2,3,5,6,7,8-hexahydro-1*H*-cyclopenta[*b*]quinolin-9-yl)butane-1,4-diamine (5a) 12](#_Toc179967506)

[Figure S7. IR spectrum of *N*^1^-(2,3,5,6,7,8-hexahydro-1*H*-cyclopenta[*b*]quinolin-9-yl)butane-1,4-diamine (5a) 13](#_Toc179967507)

[Figure S8. ^1^H NMR spectrum of *N*^1^-(2,3,5,6,7,8-hexahydro-1*H*-cyclopenta[*b*]quinolin-9-yl)hexane-1,6-diamine (5b) 14](#_Toc179967508)

[Figure S9. ^13^C NMR spectrum of *N*^1^-(2,3,5,6,7,8-hexahydro-1*H*-cyclopenta[*b*]quinolin-9-yl)hexane-1,6-diamine (5b) 15](#_Toc179967509)

[Figure S10. IR spectrum of *N*^1^-(2,3,5,6,7,8-hexahydro-1*H*-cyclopenta[*b*]quinolin-9-yl)hexane-1,6-diamine (5b) 16](#_Toc179967510)

[Figure S11. ^1^H NMR spectrum of *N*^1^-(2,3,5,6,7,8-hexahydro-1*H*-cyclopenta[*b*]quinolin-9-yl)octane-1,8-diamine (5c) 17](#_Toc179967511)

[Figure S12. ^13^C NMR spectrum of *N*^1^-(2,3,5,6,7,8-hexahydro-1*H*-cyclopenta[*b*]quinolin-9-yl)octane-1,8-diamine (5c) 18](#_Toc179967512)

[Figure S13. IR spectrum of *N*^1^-(2,3,5,6,7,8-hexahydro-1*H*-cyclopenta[*b*]quinolin-9-yl)octane-1,8-diamine (5c) 19](#_Toc179967513)

[Figure S14. ^1^H NMR spectrum of *N*-{4-[(2,3,5,6,7,8-hexahydro-1*H*-cyclopenta[*b*]quinolin-9-yl)amino]butyl}-2-hydroxybenzamide (7a) 20](#_Toc179967514)

[Figure S15. ^13^C NMR spectrum of *N*-{4-[(2,3,5,6,7,8-hexahydro-1*H*-cyclopenta[*b*]quinolin-9-yl)amino]butyl}-2-hydroxybenzamide (7a) 21](#_Toc179967515)

[Figure S16. IR spectrum of *N*-{4-[(2,3,5,6,7,8-hexahydro-1*H*-cyclopenta[*b*]quinolin-9-yl)amino]butyl}-2-hydroxybenzamide (7a) 22](#_Toc179967516)

[Figure S17. ^1^H NMR spectrum of *N*-{6-[(2,3,5,6,7,8-hexahydro-1*H*-cyclopenta[*b*]quinolin-9-yl)amino]hexyl}-2-hydroxybenzamide (7b) 23](#_Toc179967517)

[Figure S18. ^13^C NMR spectrum of *N*-{6-[(2,3,5,6,7,8-hexahydro-1*H*-cyclopenta[*b*]quinolin-9-yl)amino]hexyl}-2-hydroxybenzamide (7b) 24](#_Toc179967518)

[Figure S19. IR spectrum of *N*-{6-[(2,3,5,6,7,8-hexahydro-1*H*-cyclopenta[*b*]quinolin-9-yl)amino]hexyl}-2-hydroxybenzamide (7b) 25](#_Toc179967519)

[Figure S20. ^1^H NMR spectrum of *N*-{8-[(2,3,5,6,7,8-hexahydro-1*H*-cyclopenta[*b*]quinolin-9-yl)amino]octyl}-2-hydroxybenzamide (7c) 26](#_Toc179967520)

[Figure S21. ^13^C NMR spectrum of *N*-{8-[(2,3,5,6,7,8-hexahydro-1*H*-cyclopenta[*b*]quinolin-9-yl)amino]octyl}-2-hydroxybenzamide (7c) 27](#_Toc179967521)

[Figure S22. IR spectrum of *N*-{8-[(2,3,5,6,7,8-hexahydro-1*H*-cyclopenta[*b*]quinolin-9-yl)amino]octyl}-2-hydroxybenzamide (7c) 28](#_Toc179967522)

[Figure S23. ^1^H NMR spectrum of 2-[({4-[(2,3,5,6,7,8-hexahydro-1*H*-cyclopenta[*b*]quinolin-9-yl)amino]butyl}imino)methyl]phenol (9a) 29](#_Toc179967523)

[Figure S24. ^13^C NMR spectrum of 2-[({4-[(2,3,5,6,7,8-hexahydro-1*H*-cyclopenta[*b*]quinolin-9-yl)amino]butyl}imino)methyl]phenol (9a) 30](#_Toc179967524)

[Figure S25. IR spectrum of 2-[({4-[(2,3,5,6,7,8-hexahydro-1*H*-cyclopenta[*b*]quinolin-9-yl)amino]butyl}imino)methyl]phenol (9a) 31](#_Toc179967525)

[Figure S26. ^1^H NMR spectrum of 2-[({6-[(2,3,5,6,7,8-hexahydro-1*H*-cyclopenta[*b*]quinolin-9-yl)amino]hexyl}imino)methyl]phenol (9b) 32](#_Toc179967526)

[Figure S27. ^13^C NMR spectrum of 2-[({6-[(2,3,5,6,7,8-hexahydro-1*H*-cyclopenta[*b*]quinolin-9-yl)amino]hexyl}imino)methyl]phenol (9b) 33](#_Toc179967527)

[Figure S28. IR spectrum of 2-[({6-[(2,3,5,6,7,8-hexahydro-1*H*-cyclopenta[*b*]quinolin-9-yl)amino]hexyl}imino)methyl]phenol (9b) 34](#_Toc179967528)

[Figure S29. ^1^H NMR spectrum of 2-[({8-[(2,3,5,6,7,8-Hexahydro-1*H*-cyclopenta[*b*]quinolin-9-yl)amino]octyl}imino)methyl]phenol (9c) 35](#_Toc179967529)

[Figure S30. ^13^C NMR spectrum of 2-[({8-[(2,3,5,6,7,8-Hexahydro-1*H*-cyclopenta[*b*]quinolin-9-yl)amino]octyl}imino)methyl]phenol (9c) 36](#_Toc179967530)

[Figure S31. IR spectrum of 2-[({8-[(2,3,5,6,7,8-Hexahydro-1*H*-cyclopenta[*b*]quinolin-9-yl)amino]octyl}imino)methyl]phenol (9c) 37](#_Toc179967531)

[Figure S32. ^1^H NMR spectrum of 2-[({4-[(2,3,5,6,7,8-Hexahydro-1*H*-cyclopenta[*b*]quinolin-9-yl)amino]butyl}amino)methyl]phenol (10a) 38](#_Toc179967532)

[Figure S33. ^13^C NMR spectrum of 2-[({4-[(2,3,5,6,7,8-Hexahydro-1*H*-cyclopenta[*b*]quinolin-9-yl)amino]butyl}amino)methyl]phenol (10a) 39](#_Toc179967533)

[Figure S34. IR spectrum of 2-[({4-[(2,3,5,6,7,8-Hexahydro-1*H*-cyclopenta[*b*]quinolin-9-yl)amino]butyl}amino)methyl]phenol (10a) 40](#_Toc179967534)

[Figure S35. ^1^H NMR spectrum of 2-[({6-[(2,3,5,6,7,8-Hexahydro-1*H*-cyclopenta[*b*]quinolin-9-yl)amino]hexyl}amino)methyl]phenol (10b) 41](#_Toc179967535)

[Figure S36. ^13^C NMR spectrum of 2-[({6-[(2,3,5,6,7,8-Hexahydro-1*H*-cyclopenta[*b*]quinolin-9-yl)amino]hexyl}amino)methyl]phenol (10b) 42](#_Toc179967536)

[Figure S37. IR spectrum of 2-[({6-[(2,3,5,6,7,8-Hexahydro-1*H*-cyclopenta[*b*]quinolin-9-yl)amino]hexyl}amino)methyl]phenol (10b) 43](#_Toc179967537)

[Figure S38. ^1^H NMR spectrum of 2-[({8-[(2,3,5,6,7,8-Hexahydro-1*H*-cyclopenta[*b*]quinolin-9-yl)amino]octyl}amino)methyl]phenol (10c) 44](#_Toc179967538)

[Figure S39. ^13^C NMR spectrum of 2-[({8-[(2,3,5,6,7,8-Hexahydro-1*H*-cyclopenta[*b*]quinolin-9-yl)amino]octyl}amino)methyl]phenol (10c) 45](#_Toc179967539)

[Figure S40. IR spectrum of 2-[({8-[(2,3,5,6,7,8-Hexahydro-1*H*-cyclopenta[*b*]quinolin-9-yl)amino]octyl}amino)methyl]phenol (10c) 46](#_Toc179967540)

[Figure S41. ^1^H NMR spectrum of *N*-hexyl-2,3,5,6,7,8-hexahydro-1*H*-cyclopenta[*b*]quinolin-9-amine (11) 47](#_Toc179967541)

[Figure S42. ^1^C NMR spectrum of *N*-hexyl-2,3,5,6,7,8-hexahydro-1*H*-cyclopenta[*b*]quinolin-9-amine (11) 48](#_Toc179967542)

[Figure S43. IR spectrum of *N*-hexyl-2,3,5,6,7,8-hexahydro-1*H*-cyclopenta[*b*]quinolin-9-amine (11) 49](#_Toc179967543)

[Figure S44. IR spectrum of 2-[(hexylimino)methyl]phenol (12) 50](#_Toc179967544)

[Figure S45. ^1^H NMR spectrum of 2-[(hexylamino)methyl]phenol (13) 51](#_Toc179967545)

[Figure S46. ^1^C NMR spectrum of 2-[(hexylamino)methyl]phenol (13) 52](#_Toc179967546)

[Figure S47. IR spectrum of 2-[(hexylamino)methyl]phenol (13) 53](#_Toc179967547)

[Figure S48. (a) UV spectra of compound 7c and mixtures of 7c with Cu^2+^, Fe^2+^, and Zn^2+^ ions. (b) UV spectra of compound 9c and mixtures of 9c with Cu^2+^, Fe^2+^, and Zn^2+^ ions 54](#_Toc179967548)

[Figure S49. (a) UV spectra of compound 11 and mixtures of 11 with Cu^2+^, Fe^2+^, and Zn^2+^ ions. (b) UV spectra of compound 12 and mixtures of 12 with Cu^2+^, Fe^2+^, and Zn^2+^ ions 55](#_Toc179967549)

[Figure S50. (a) UV spectra of the 7c–Cu^2+^, 14–Cu^2+^, 11–Cu^2+^ complexes, obtained subtracting the spectra of ions and compounds from the spectra of mixtures. (b) UV spectra of the 10c–Cu^2+^, 13–Cu^2+^, 11–Cu^2+^ complexes, obtained subtracting the spectra of ions and compounds from the spectra of mixtures. 56](#_Toc179967550)

[Figure S51. (a) UV absorption spectra of 7c (20 µM) in EtOH after addition of increasing concentrations of CuCl_2_ (2–34 µM). (b) the differential spectra due to the 7c–Cu^2+^ complex formation obtained by numerical subtraction from the spectra of the mixture of the spectra of the Cu^2+^ alone and 7c alone at the corresponding concentrations. 57](#_Toc179967551)

[Figure S52. (a) UV absorption spectra of 9c (20 µM) in EtOH after addition of increasing concentrations of CuCl_2_ (2–34 µM). (b) the differential spectra due to the 9c–Cu^2+^ complex formation obtained by numerical subtraction from the spectra of the mixture of the spectra of the Cu^2+^ alone and 9c alone at the corresponding concentrations. 58](#_Toc179967552)

[Figure S53. (a) UV absorption spectra of 10c (20 µM) in EtOH after addition of increasing concentrations of CuCl_2_ (2–34 µM). (b) the differential spectra due to the 10c–Cu^2+^ complex formation obtained by numerical subtraction from the spectra of the mixture of the spectra of the Cu^2+^ alone and 10c alone at the corresponding concentrations. 59](#_Toc179967553)

[Figure S54. (a) Absorbance of the 7c–Cu^2+^ complex at 352 nm as a function of the concentration of Cu^2+^ (compounds concentration is 20 µM). Vertical dashed lines mark the metal concentration at the breakpoints and indicate ligand–metal molar ratios of 1.05:1. (b) Absorbance of the 9c–Cu^2+^ complex at 352 nm as a function of the concentration of Cu^2+^ (compounds concentration is 40 µM). Vertical dashed lines mark the metal concentration at the breakpoints and indicate ligand–metal molar ratios of 1.7:1. 60](#_Toc179967554)

[Figure S55. (a) UV absorption spectra of 11 (20 µM) in EtOH after addition of increasing concentrations of CuCl_2_ (2–42 µM). (b) the differential spectra due to the 11–Cu^2+^ complex formation obtained by numerical subtraction from the spectra of the mixture of the spectra of the Cu^2+^ alone and 11 alone at the corresponding concentrations. (с) absorbance of the 11–Cu^2+^ complex at 287 nm as a function of the concentration of Cu^2+^ (compounds concentration is 20 µM). Vertical dashed lines mark the metal concentration at the breakpoints and indicate ligand–metal molar ratios of 0.9:1. 61](#_Toc179967555)

[Figure S56. Cytotoxic effect (IC_50_, μM) toward HEK293T for conjugates of salicylic derivatives 7a, 9a and 10a. 62](#_Toc179967556)

[Figure S57. Cytotoxic effect (IC_50_, μM) toward SH-SY5Y for conjugates of salicylic derivatives 7a, 9a and 10a. 63](#_Toc179967557)

[Figure S58. Cytotoxic effect (IC_50_, μM) toward HepG2 for conjugates of salicylic derivatives 7a, 9a and 10a. 64](#_Toc179967558)

[Figure S59. Cytotoxic effect (IC_50_, μM) toward HEK293T for conjugates of amiridine and salicylic derivatives 7c, 9c and 10c. 65](#_Toc179967559)

[Figure S60. Cytotoxic effect (IC_50_, μM) toward SH-SY5Y for conjugates of amiridine and salicylic derivatives 7c, 9c and 10c. 66](#_Toc179967560)

[Figure S61. Cytotoxic effect (IC_50_, μM) toward HepG2 for conjugates of amiridine and salicylic derivatives 7c, 9c and 10c. 67](#_Toc179967561)

# Quantum Chemical Analyses of Antioxidant Activity (AOA)

Before analyzing the ABTS test results, the protonation state of the studied compounds was estimated. Under the experimental conditions of the ABTS test, the pH is about 5. However, this acidic media is the result of dissolving 2,2’-Azinobis(3-ethylbenzothiazoline-6-sulfonic acid) diammonium salt in the solvent (90% ethanol, 8% water, 2% DMSO), and not supported by any buffer. Therefore, the state of protonation is determined by the advantage of proton transfer from the NH_4_^+^ cation to the compounds under study. To determine the protonation state, the proton affinity (PA) of ammonia and the studied compounds was calculated (see Table S1).

Table S1. The calculated PA values

|  | PA_1_^1^, kcal/mol | PA_2_^2^, kcal/mol |
| --- | --- | --- |
| NH_3_ | 24.3 | n/a |
| **7a** | 33.5 | n/a |
| **9a** | 33.4 | 21.1 |
| **10a** | 33.6 | 25.1 |

^1^for compounds **7a, 9a, 10a** under protonation of endocyclic N atom of amiridine fragment

^2^for compounds **9a** and **10a** under protonation of imine and amine N atom of salicylate fragment, respectively

Table S1 shows that proton transfer from NH_4_^+^ to the endocyclic N atom of amiridine fragment is energetically favorable for all compounds. The proton transfer from NH_4_^+^ to the imine N atom in **9a** is energetically unfavorable, while the proton transfer from NH_4_^+^ to the amine N atom in **10a** is energetically favorable. Thus, in the ABTS test, compounds **7a** and **9a** are once protonated at the amiridine moiety, and compound **10a** is doubly protonated at the amiridine and salicylate moieties.

To characterize the AOA of the studied compounds, their bond dissociation enthalpy (BDE) and ionization potential (IP) were calculated (see Table S2).

Table S2. Calculated BDE and IP values

|  | BDE, kcal/mol | IP, kcal/mol |
| --- | --- | --- |
| **7a_a_** | 87.4 | 118.1 |
| **9a_a_** | 90.2 | 105.0 |
| **10a_as_** | 83.5 | 125.8 |

Table S2 shows that BDE, not IP, value correlates with the AOA of amiridine-salicylate conjugates. The antiradical action of the studied compounds occurs through the H-atom abstraction, when an electron is transferred to the ABTS^•+^ radical and a proton simultaneously passes into the solvent.

The proton acceptors in the solvent are SO_4_^2-^ anions, which are formed during the ABTS^•+^ radicals generation in the following reactions ^[1]^.

ABTS^2-^ + S_2_O_8_^2-^ → ABTS^•-^ + SO_4_^•-^ + SO_4_^2-^ (1)

ABTS^2-^ + SO_4_^•-^ → ABTS^•-^ + SO_4_^2-^. (2)

In reactions (1)-(2), one SO_4_^2-^ anion is formed per each ABTS^•+^ radical.

**REFERENCES**

[1] I.R. Ilyasov, V.L. Beloborodov, I.A. Selivanova, R.P. Terekhov, *Int. J. Mol. Sci.* **2020**, *21*, 1131. DOI: 10.3390/ijms21031131

# Figure S1. ^1^H NMR spectrum of 1,2,3,4,5,6,7,8-octahydro-9H-cyclopenta[b]quinolin-9-one (2)


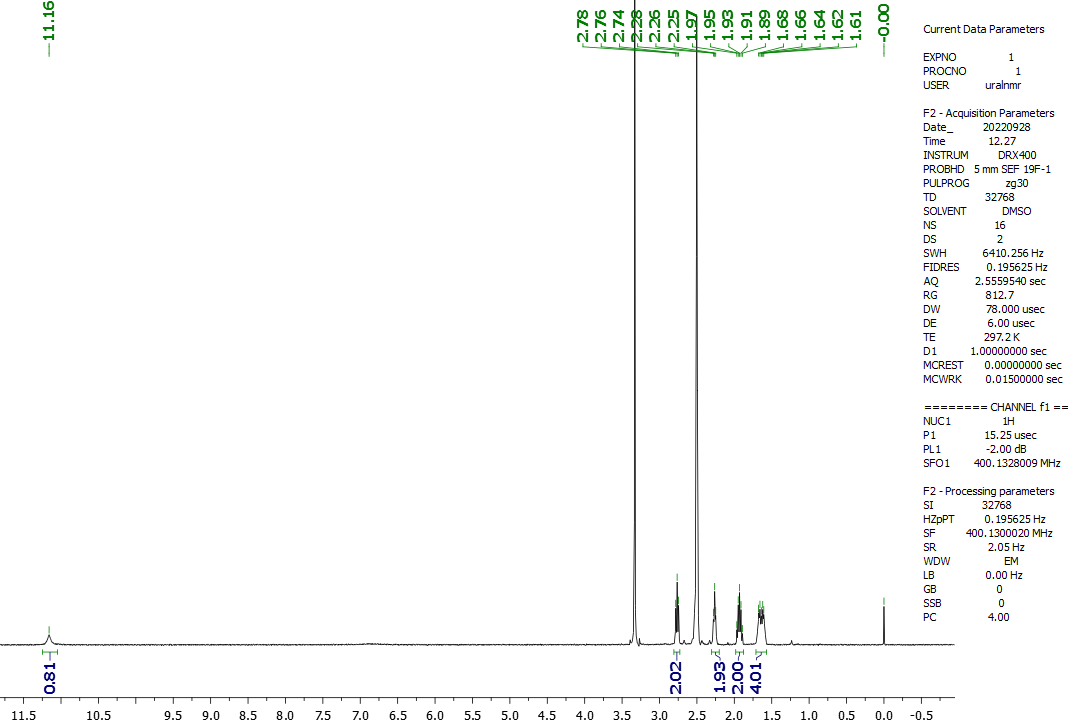


# Figure S2. ^13^C NMR spectrum of 1,2,3,4,5,6,7,8-octahydro-9H-cyclopenta[b]quinolin-9-one (2)


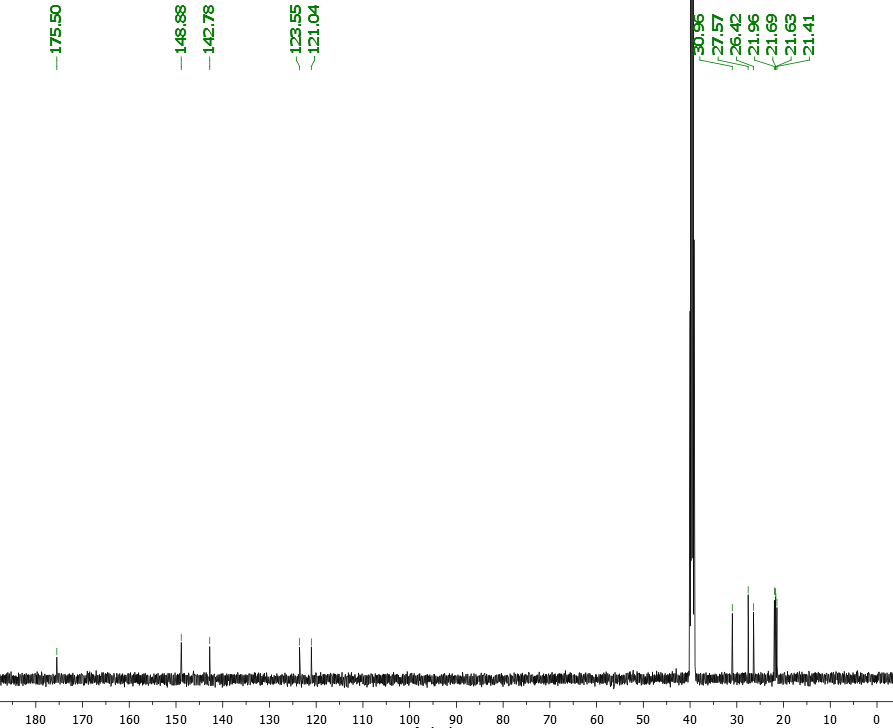


# Figure S3. ^1^H NMR spectrum of 9-chloro-2,3,5,6,7,8-hexahydro-1*H*-cyclopenta[*b*]quinolone (3)


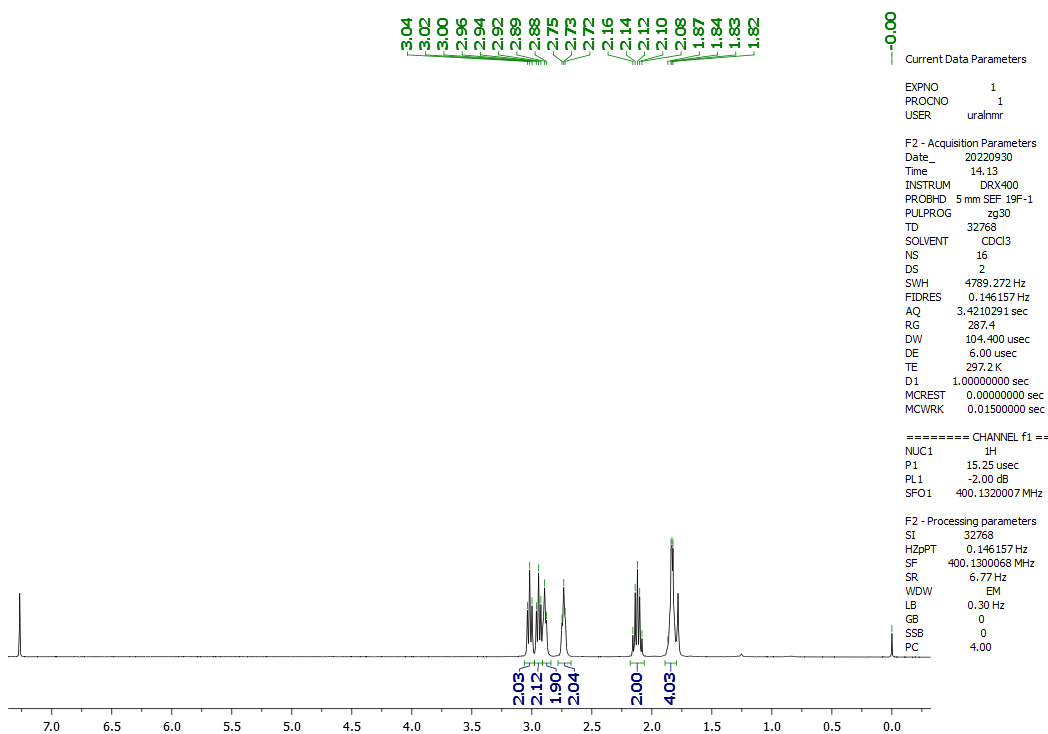


# Figure S4. ^13^C NMR spectrum of 9-chloro-2,3,5,6,7,8-hexahydro-1*H*-cyclopenta[*b*]quinolone (3)


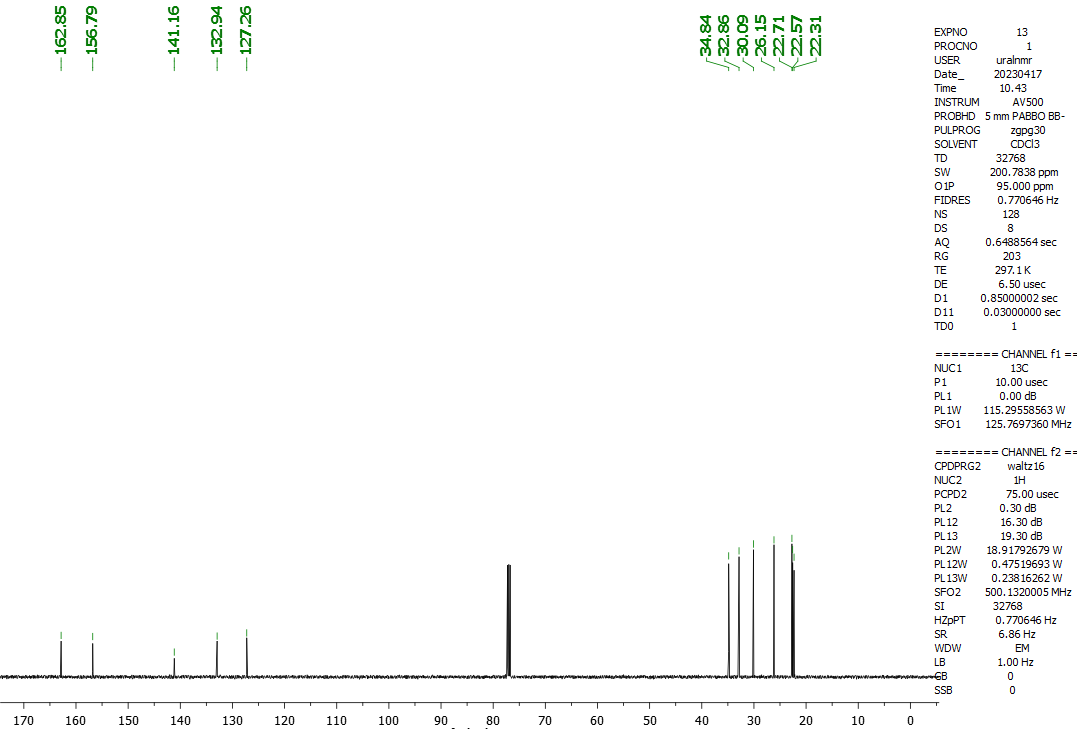

#
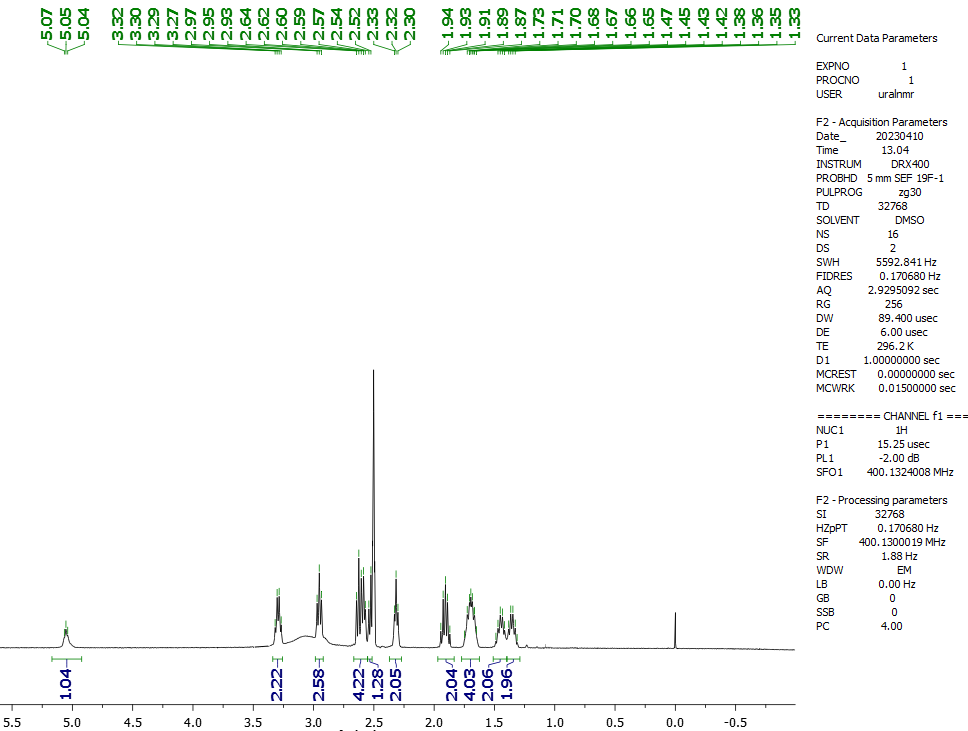
Figure S5. ^1^H NMR spectrum of *N*^1^-(2,3,5,6,7,8-hexahydro-1*H*-cyclopenta[*b*]quinolin-9-yl)butane-1,4-diamine (5a)

# Figure S6. ^13^C NMR spectrum of *N*^1^-(2,3,5,6,7,8-hexahydro-1*H*-cyclopenta[*b*]quinolin-9-yl)butane-1,4-diamine (5a)


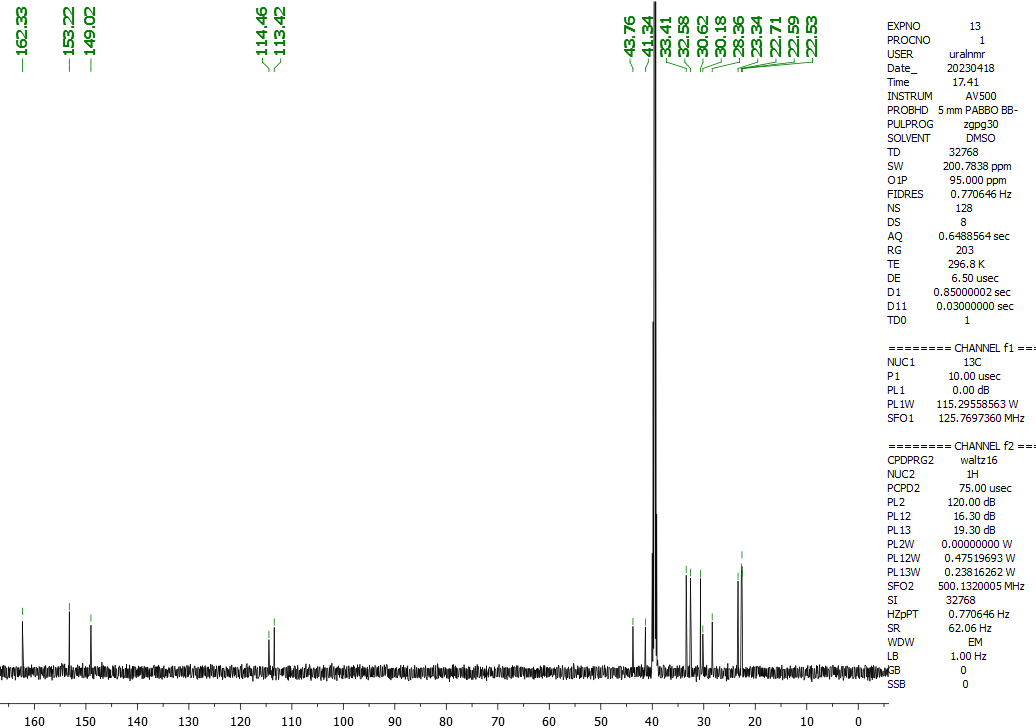


# Figure S7. IR spectrum of *N*^1^-(2,3,5,6,7,8-hexahydro-1*H*-cyclopenta[*b*]quinolin-9-yl)butane-1,4-diamine (5a)

**
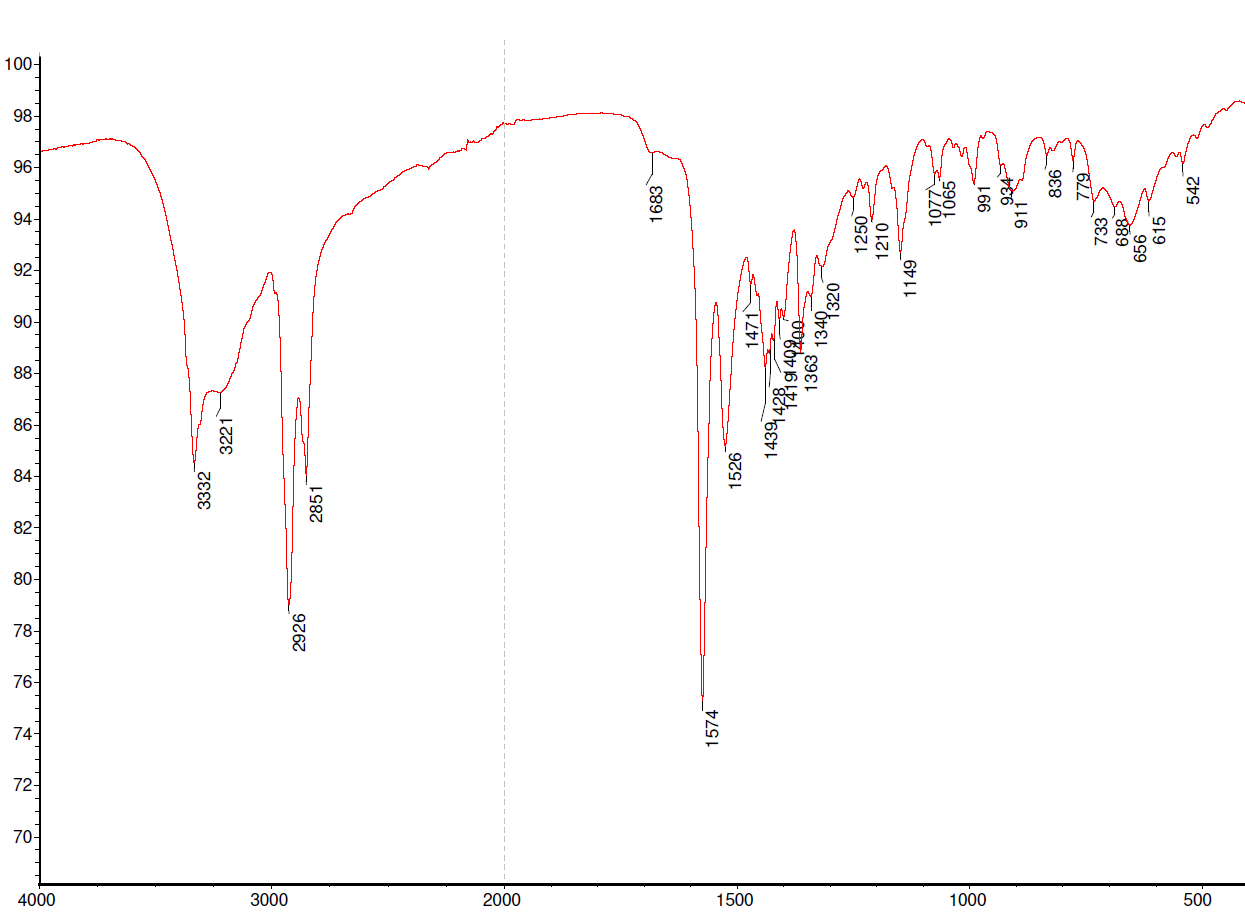
**

# Figure S8. ^1^H NMR spectrum of *N*^1^-(2,3,5,6,7,8-hexahydro-1*H*-cyclopenta[*b*]quinolin-9-yl)hexane-1,6-diamine (5b)


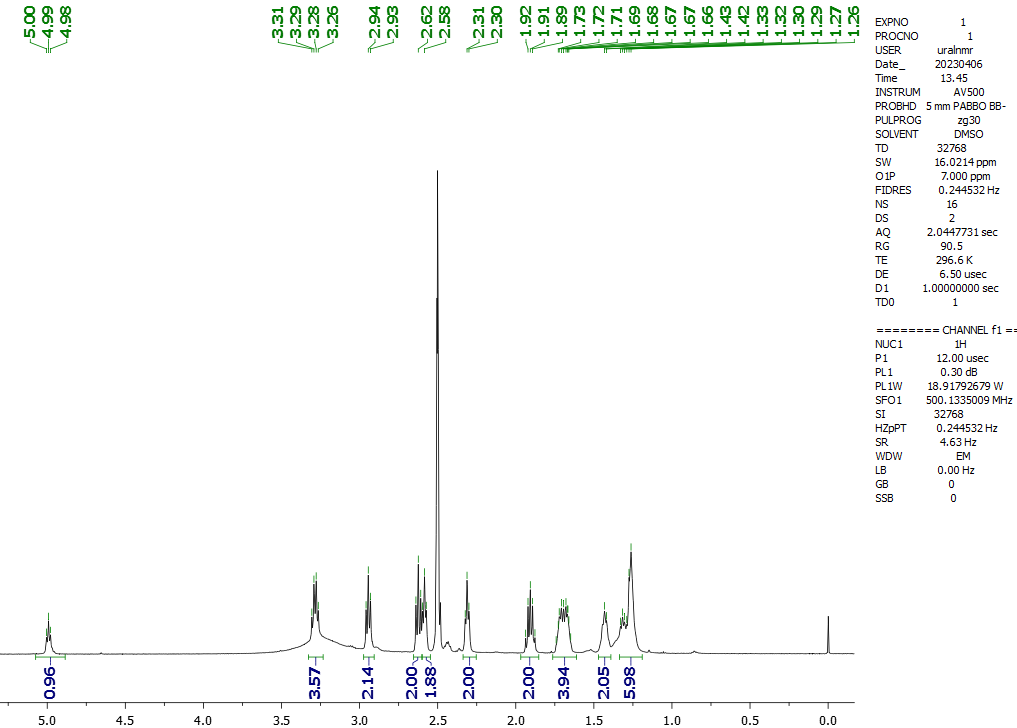


# Figure S9. ^13^C NMR spectrum of *N*^1^-(2,3,5,6,7,8-hexahydro-1*H*-cyclopenta[*b*]quinolin-9-yl)hexane-1,6-diamine (5b)


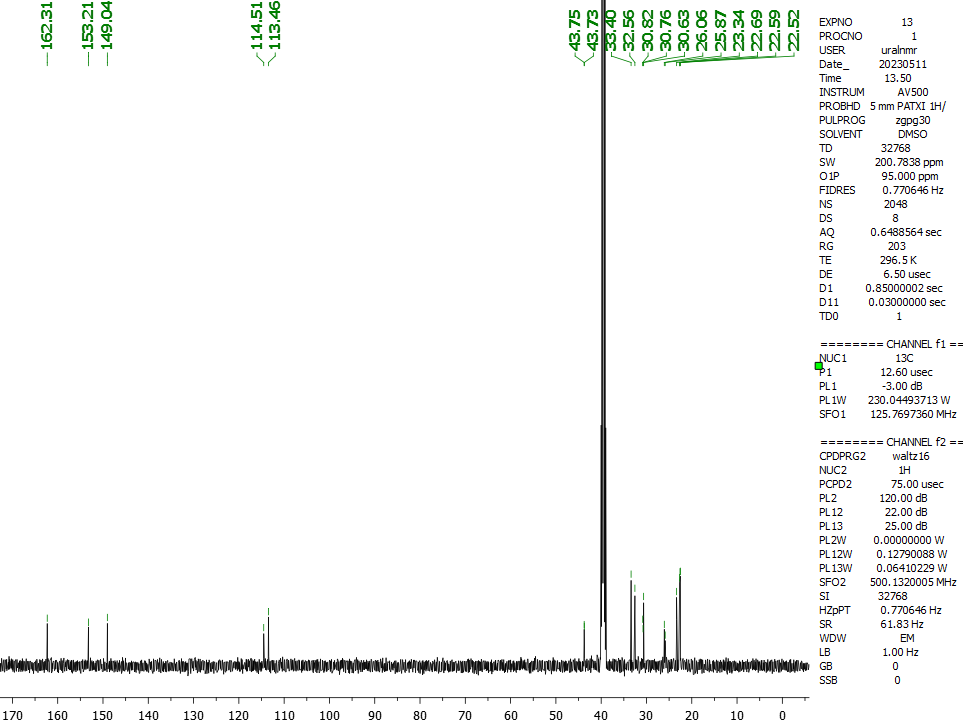


# Figure S10. IR spectrum of *N*^1^-(2,3,5,6,7,8-hexahydro-1*H*-cyclopenta[*b*]quinolin-9-yl)hexane-1,6-diamine (5b)

**
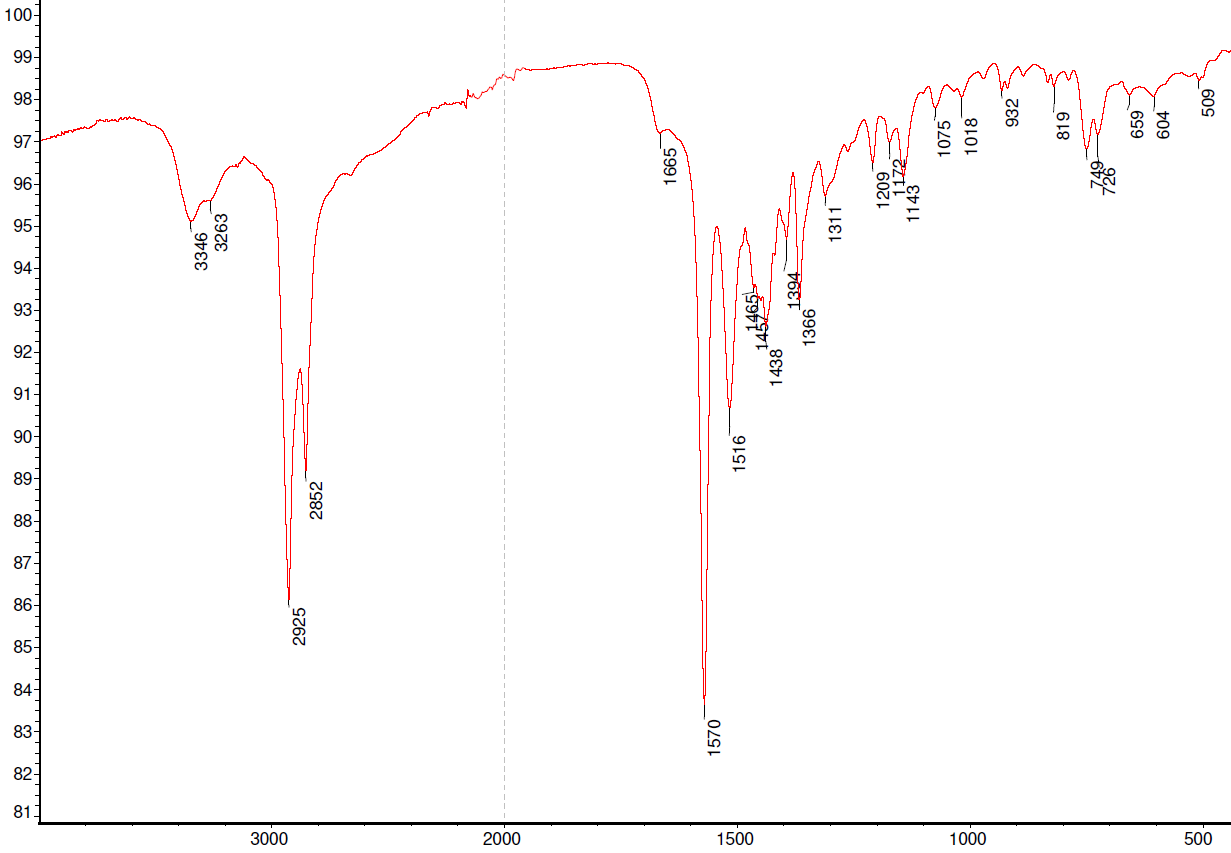
**

# Figure S11. ^1^H NMR spectrum of *N*^1^-(2,3,5,6,7,8-hexahydro-1*H*-cyclopenta[*b*]quinolin-9-yl)octane-1,8-diamine (5c)


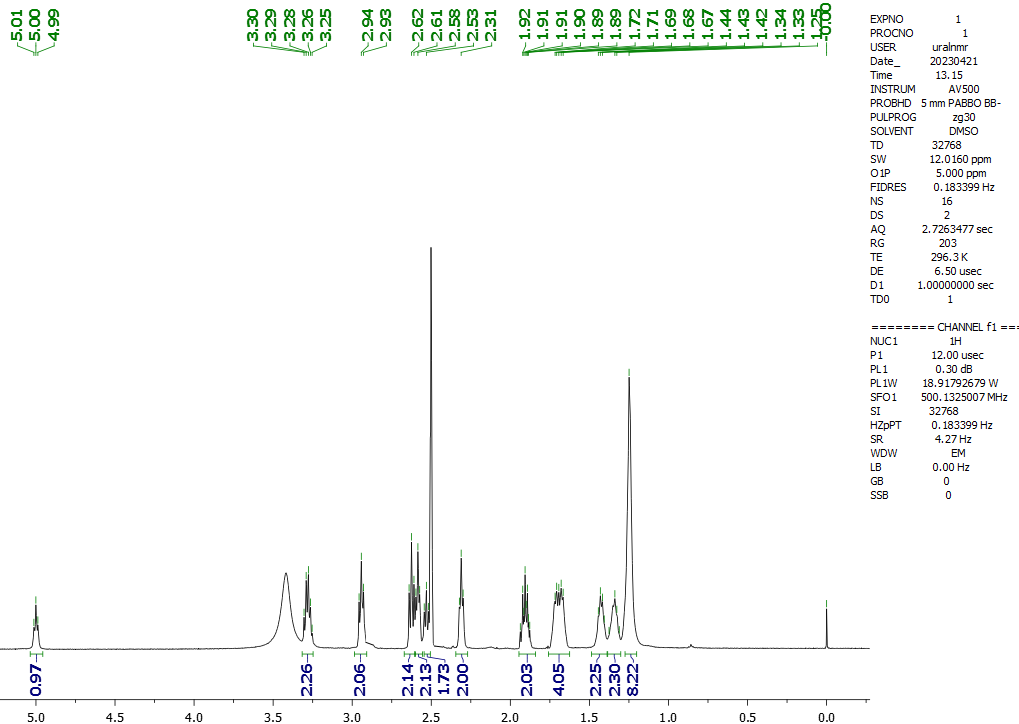


# Figure S12. ^13^C NMR spectrum of *N*^1^-(2,3,5,6,7,8-hexahydro-1*H*-cyclopenta[*b*]quinolin-9-yl)octane-1,8-diamine (5c)


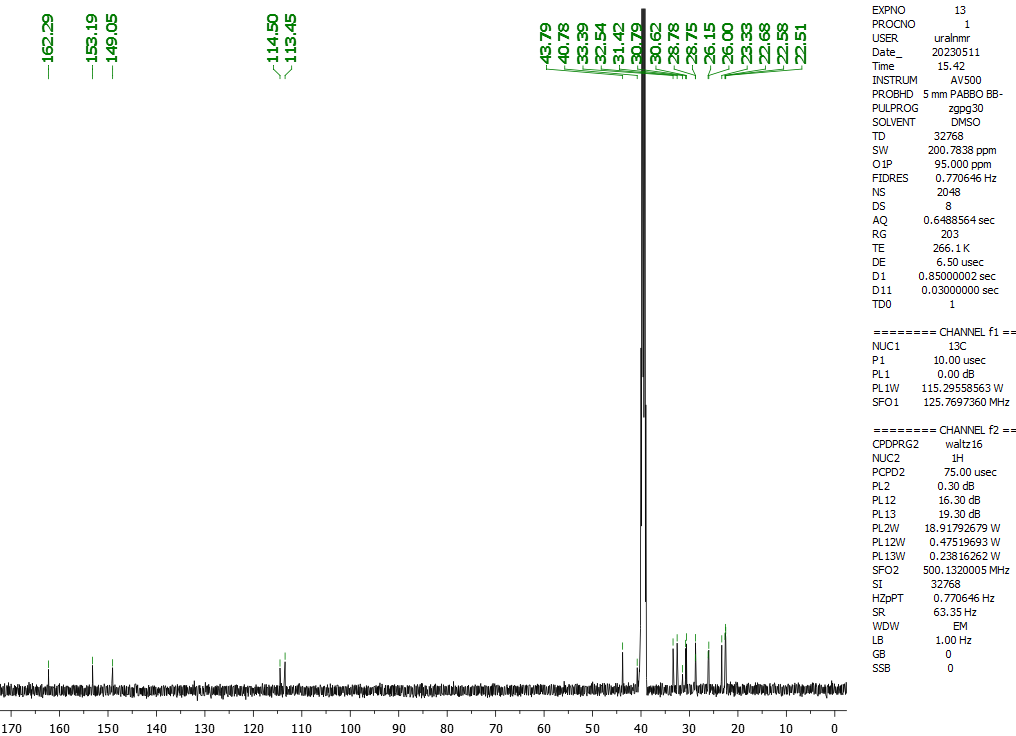


# Figure S13. IR spectrum of *N*^1^-(2,3,5,6,7,8-hexahydro-1*H*-cyclopenta[*b*]quinolin-9-yl)octane-1,8-diamine (5c)

**
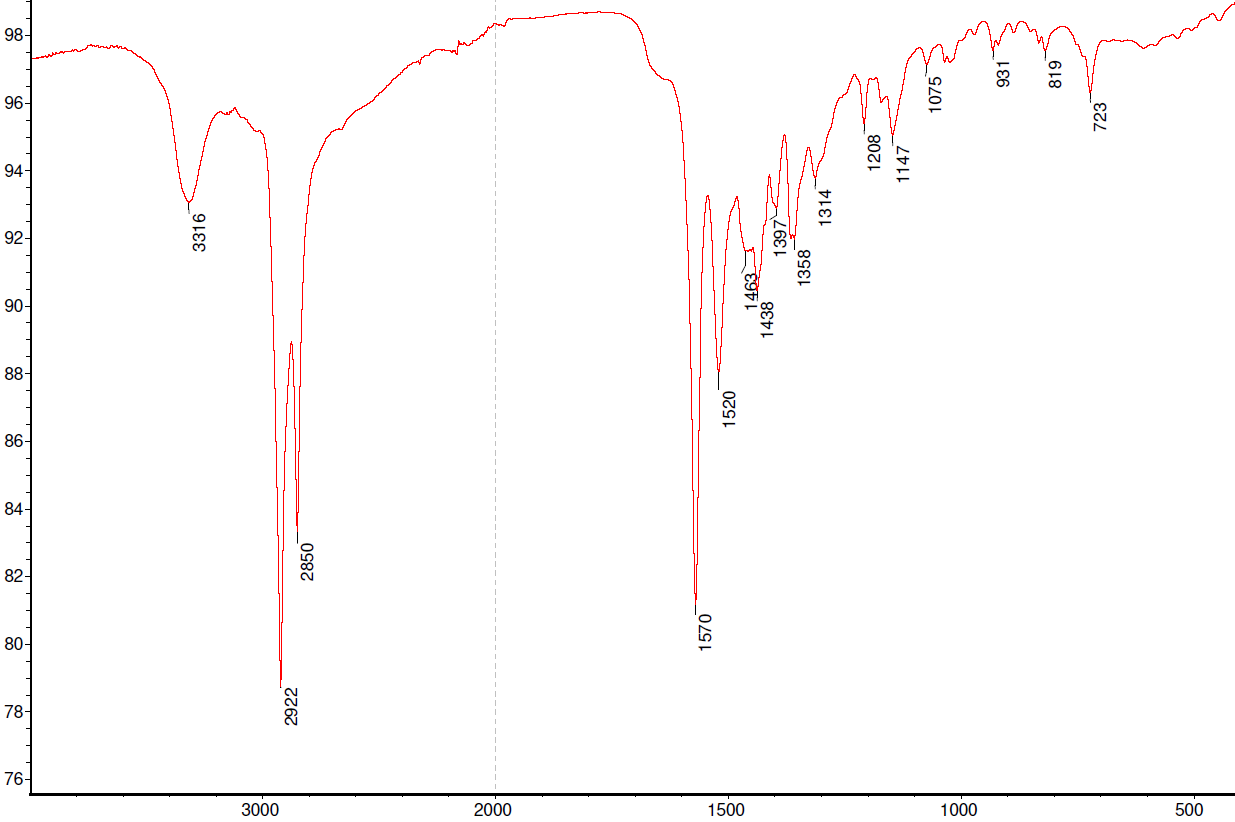
**

# Figure S14. ^1^H NMR spectrum of *N*-{4-[(2,3,5,6,7,8-hexahydro-1*H*-cyclopenta[*b*]quinolin-9-yl)amino]butyl}-2-hydroxybenzamide (7a)


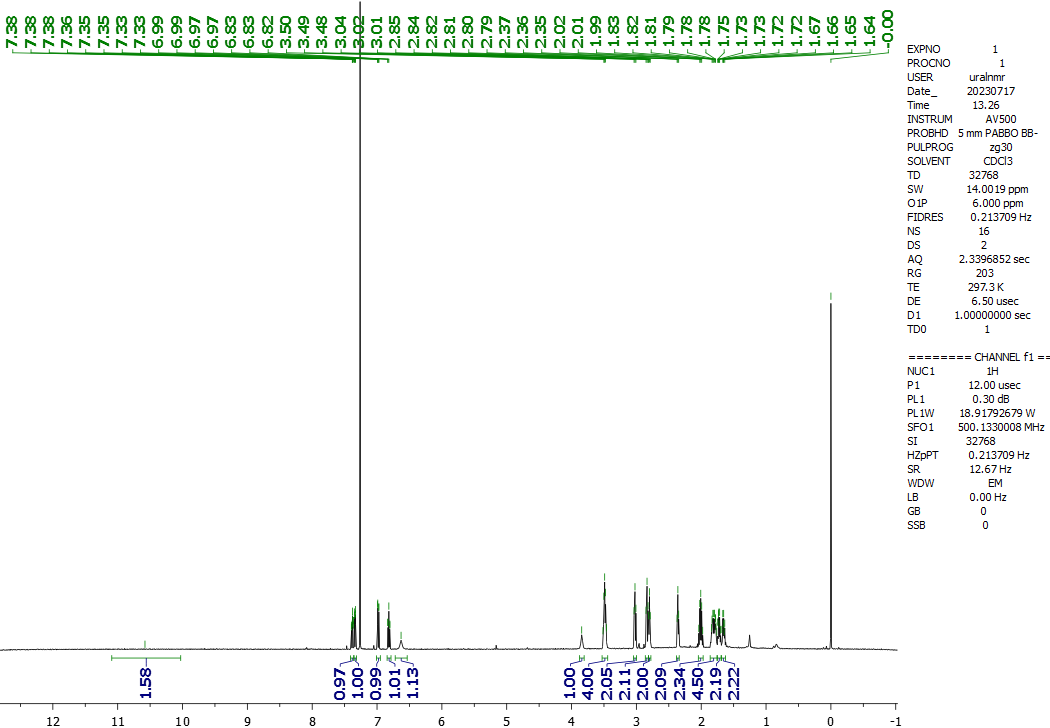


# Figure S15. ^13^C NMR spectrum of *N*-{4-[(2,3,5,6,7,8-hexahydro-1*H*-cyclopenta[*b*]quinolin-9-yl)amino]butyl}-2-hydroxybenzamide (7a)


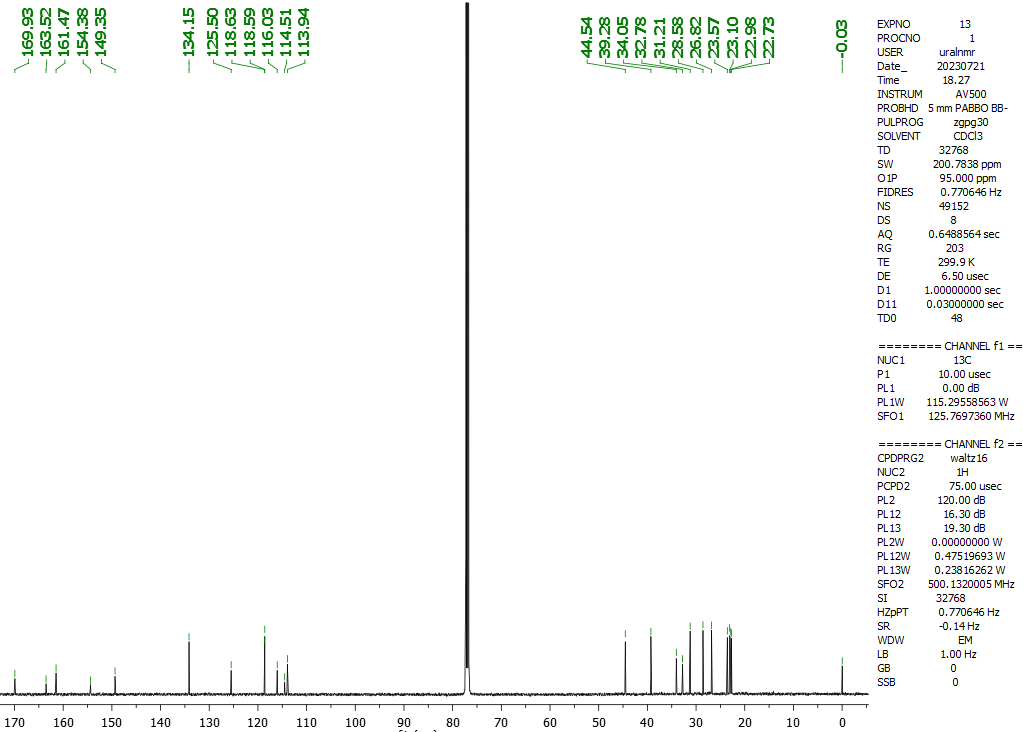


# Figure S16. IR spectrum of *N*-{4-[(2,3,5,6,7,8-hexahydro-1*H*-cyclopenta[*b*]quinolin-9-yl)amino]butyl}-2-hydroxybenzamide (7a)

**
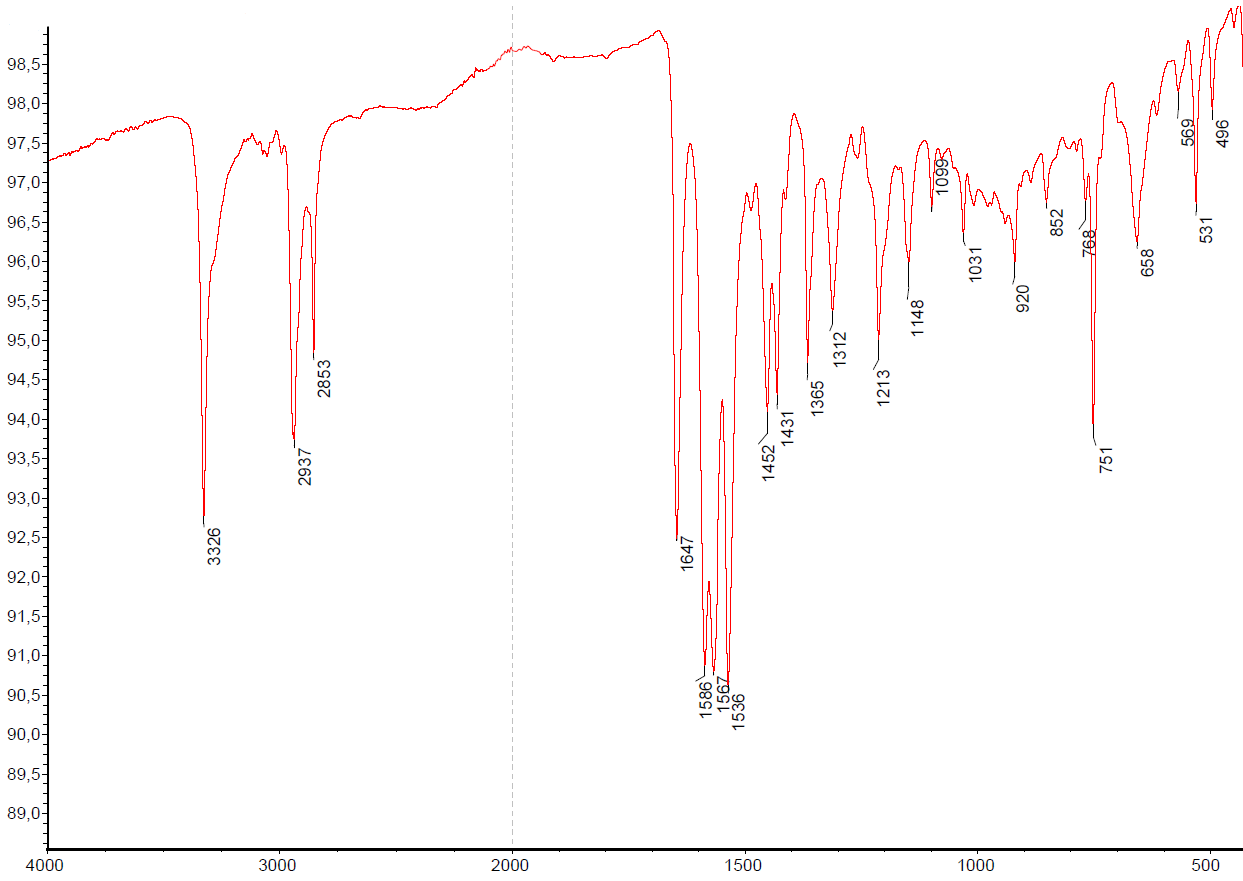
**

# Figure S17. ^1^H NMR spectrum of *N*-{6-[(2,3,5,6,7,8-hexahydro-1*H*-cyclopenta[*b*]quinolin-9-yl)amino]hexyl}-2-hydroxybenzamide (7b)


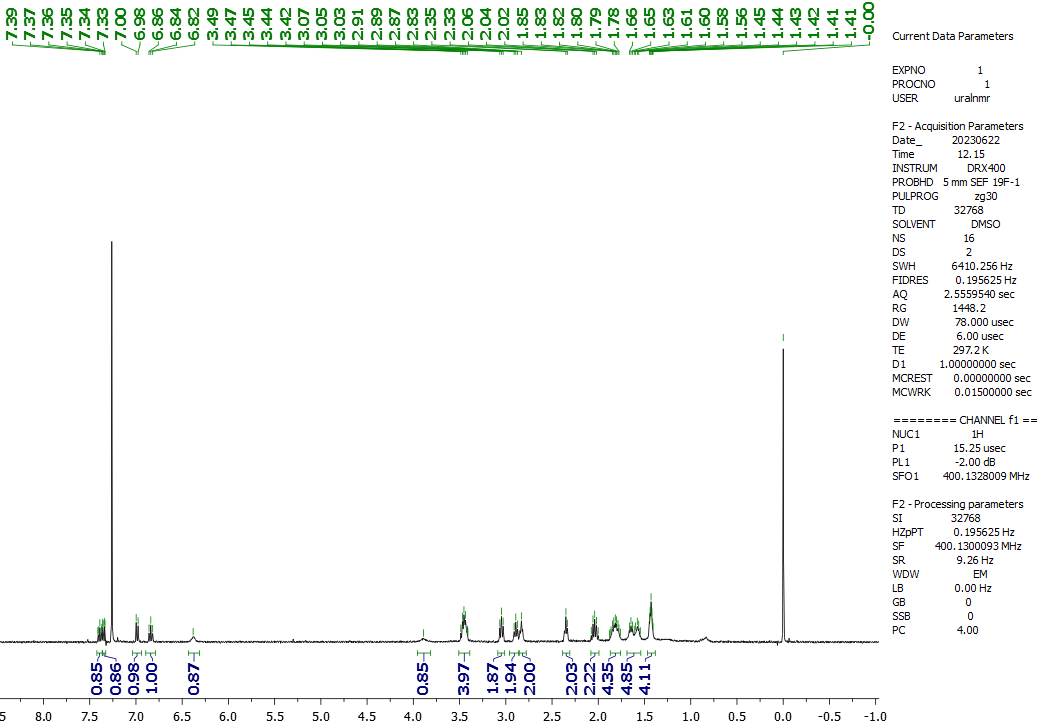


# Figure S18. ^13^C NMR spectrum of *N*-{6-[(2,3,5,6,7,8-hexahydro-1*H*-cyclopenta[*b*]quinolin-9-yl)amino]hexyl}-2-hydroxybenzamide (7b)


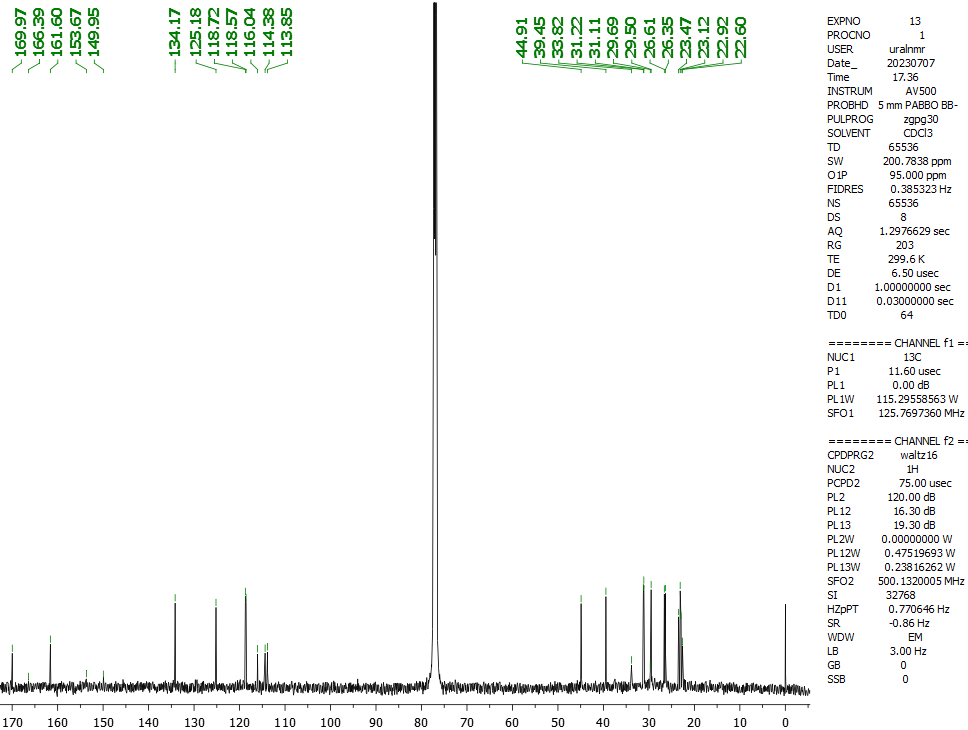


# Figure S19. IR spectrum of *N*-{6-[(2,3,5,6,7,8-hexahydro-1*H*-cyclopenta[*b*]quinolin-9-yl)amino]hexyl}-2-hydroxybenzamide (7b)

**
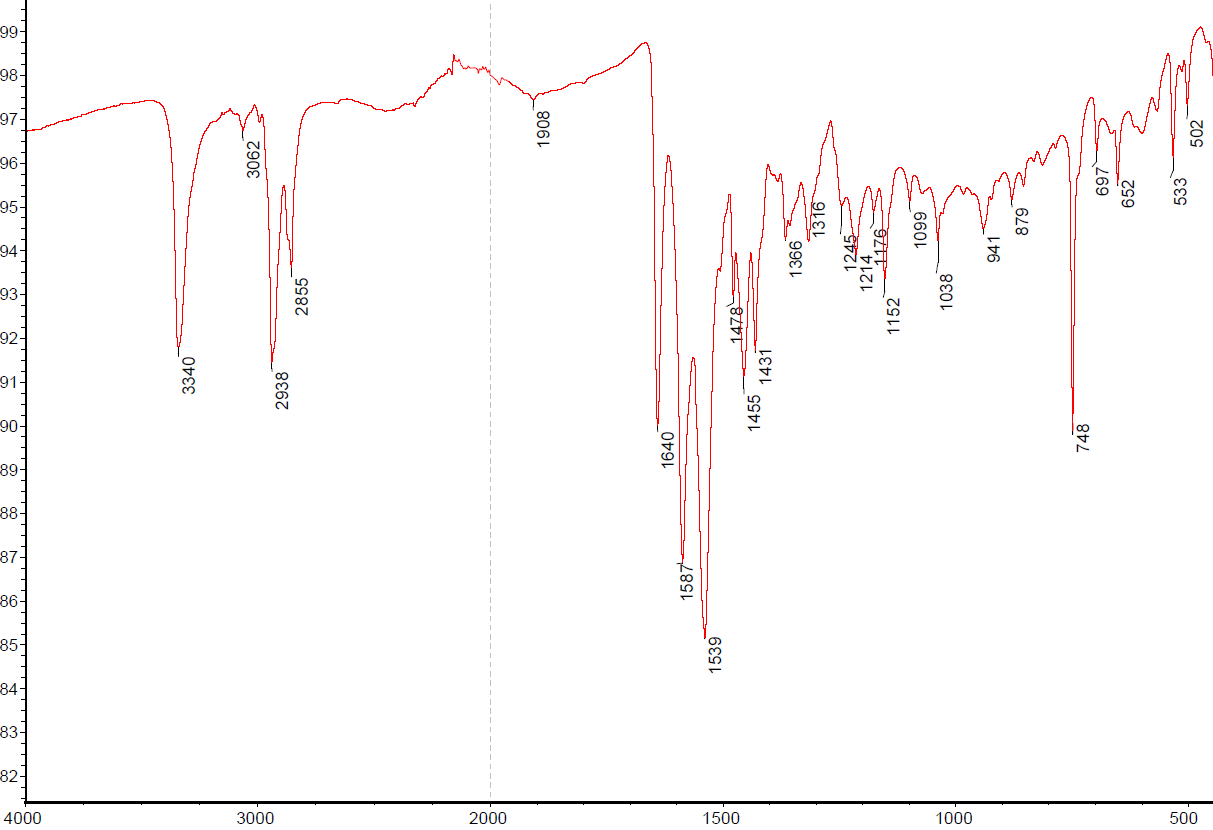
**

# Figure S20. ^1^H NMR spectrum of *N*-{8-[(2,3,5,6,7,8-hexahydro-1*H*-cyclopenta[*b*]quinolin-9-yl)amino]octyl}-2-hydroxybenzamide (7c)


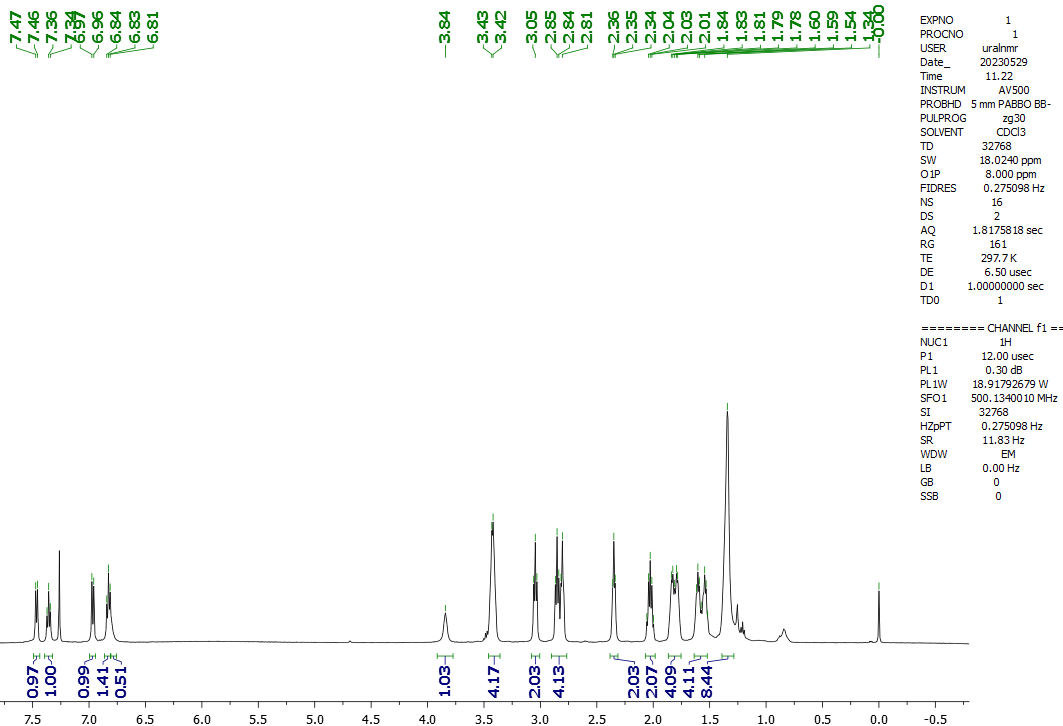


# Figure S21. ^13^C NMR spectrum of *N*-{8-[(2,3,5,6,7,8-hexahydro-1*H*-cyclopenta[*b*]quinolin-9-yl)amino]octyl}-2-hydroxybenzamide (7c)


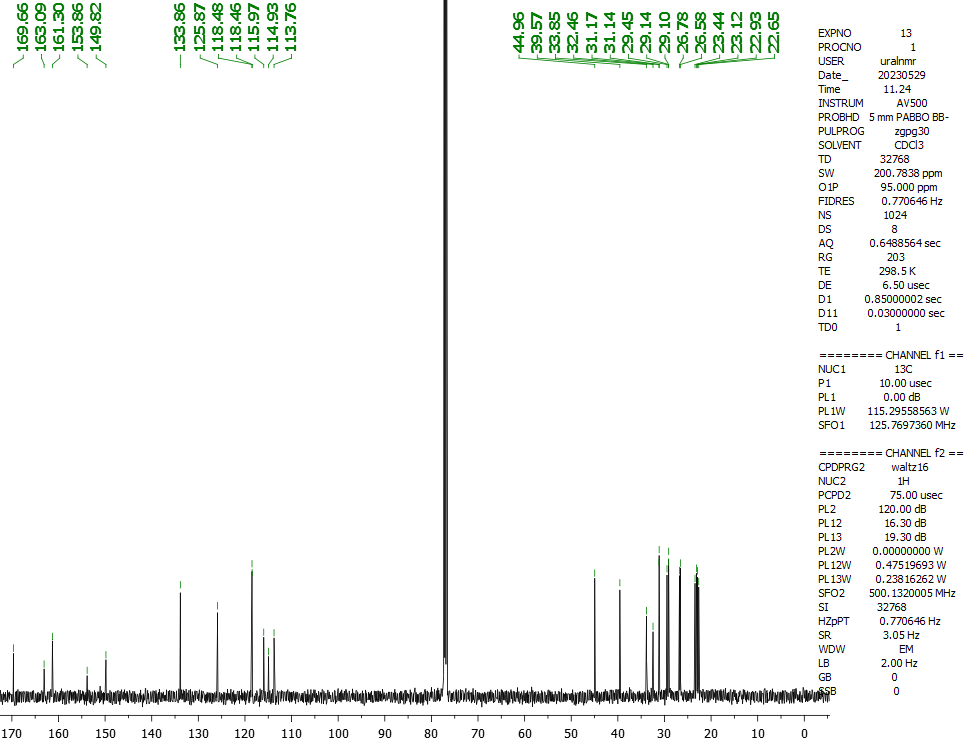


# Figure S22. IR spectrum of *N*-{8-[(2,3,5,6,7,8-hexahydro-1*H*-cyclopenta[*b*]quinolin-9-yl)amino]octyl}-2-hydroxybenzamide (7c)

**
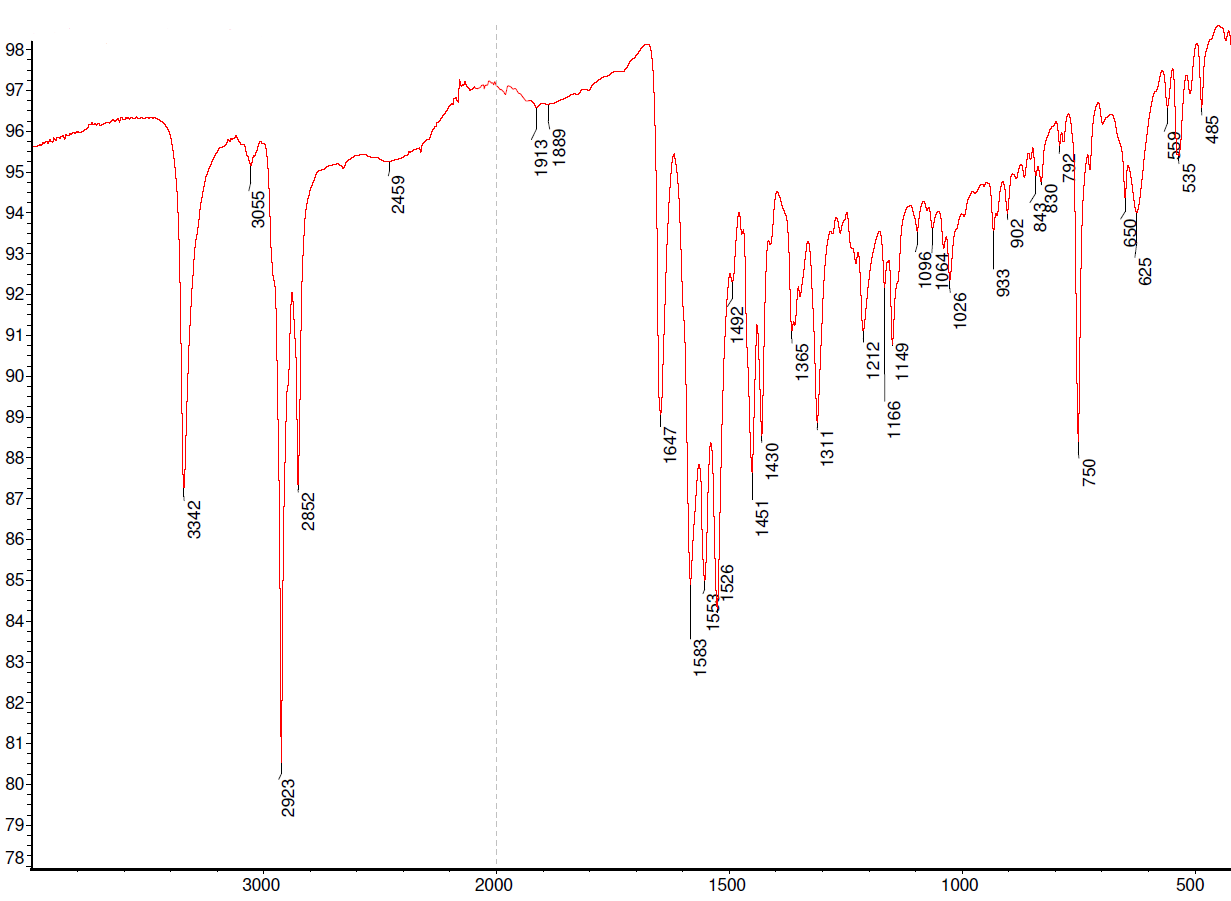
**

# Figure S23. ^1^H NMR spectrum of 2-[({4-[(2,3,5,6,7,8-hexahydro-1*H*-cyclopenta[*b*]quinolin-9-yl)amino]butyl}imino)methyl]phenol (9a)


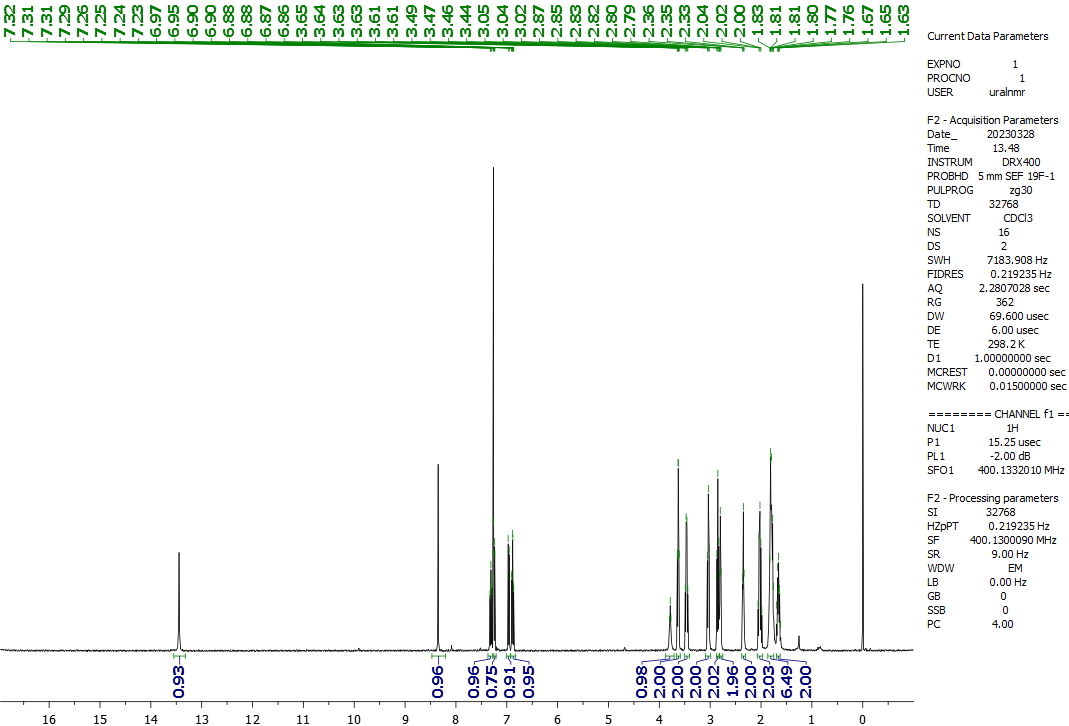


# Figure S24. ^13^C NMR spectrum of 2-[({4-[(2,3,5,6,7,8-hexahydro-1*H*-cyclopenta[*b*]quinolin-9-yl)amino]butyl}imino)methyl]phenol (9a)


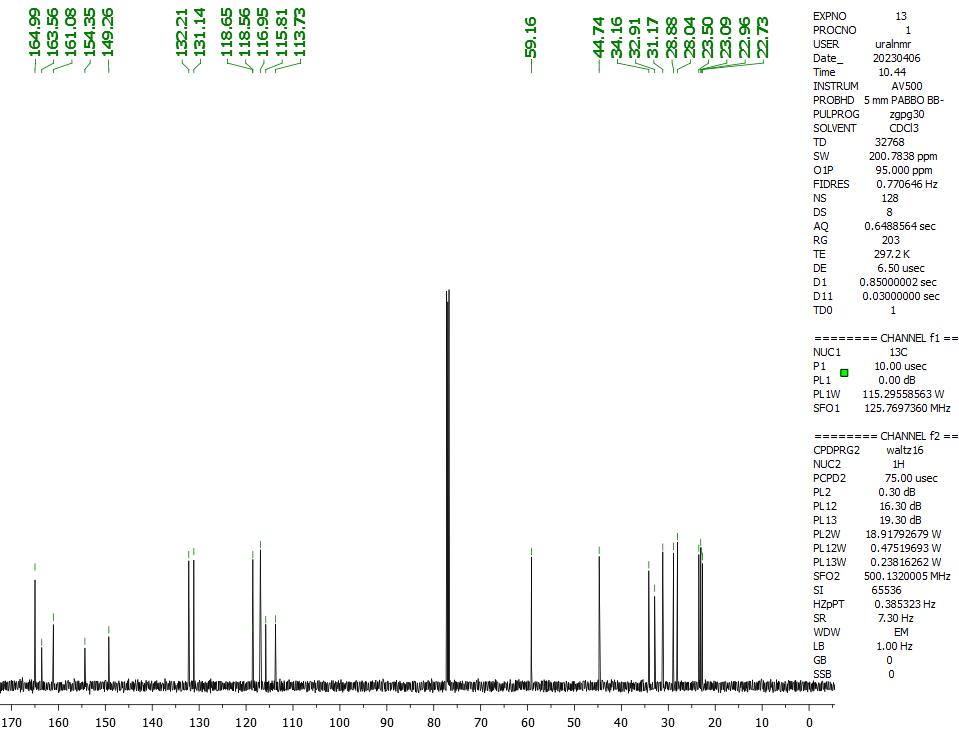


# Figure S25. IR spectrum of 2-[({4-[(2,3,5,6,7,8-hexahydro-1*H*-cyclopenta[*b*]quinolin-9-yl)amino]butyl}imino)methyl]phenol (9a)

**
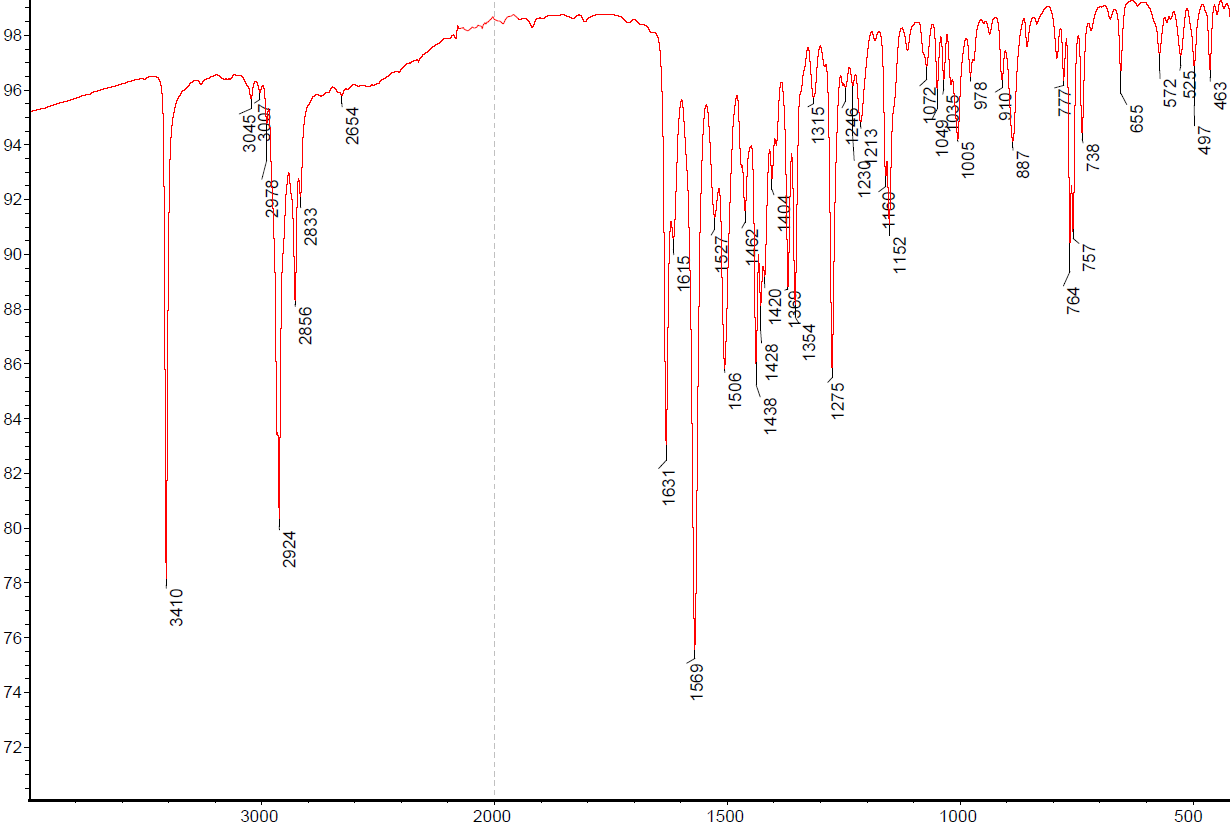
**

# Figure S26. ^1^H NMR spectrum of 2-[({6-[(2,3,5,6,7,8-hexahydro-1*H*-cyclopenta[*b*]quinolin-9-yl)amino]hexyl}imino)methyl]phenol (9b)


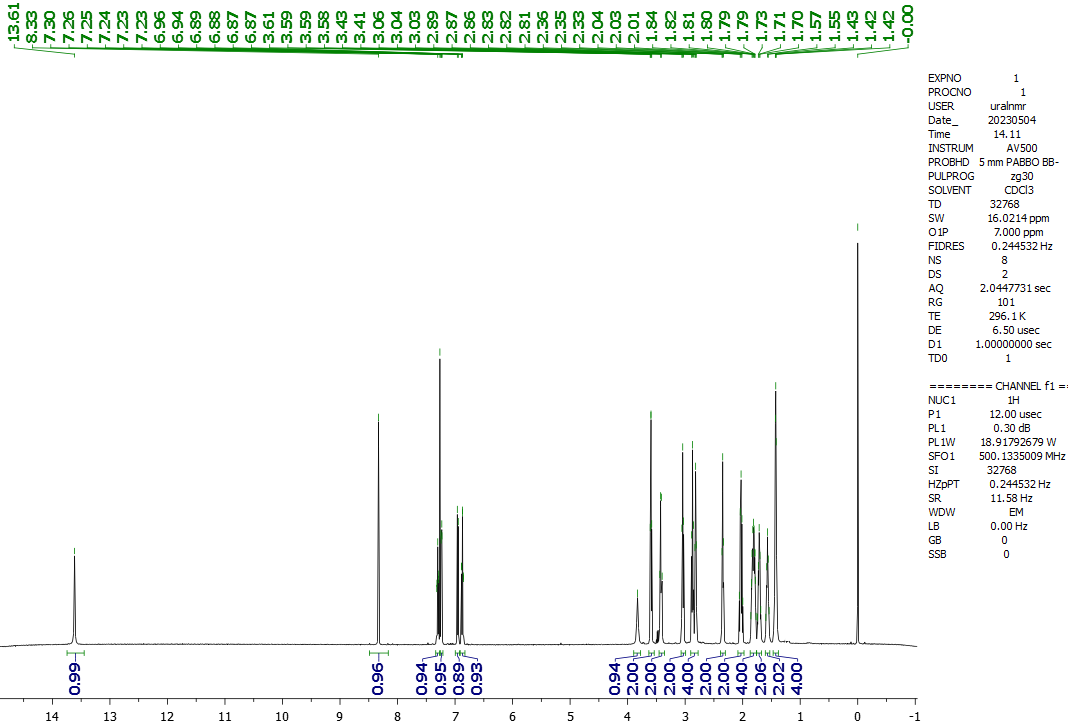


# Figure S27. ^13^C NMR spectrum of 2-[({6-[(2,3,5,6,7,8-hexahydro-1*H*-cyclopenta[*b*]quinolin-9-yl)amino]hexyl}imino)methyl]phenol (9b)


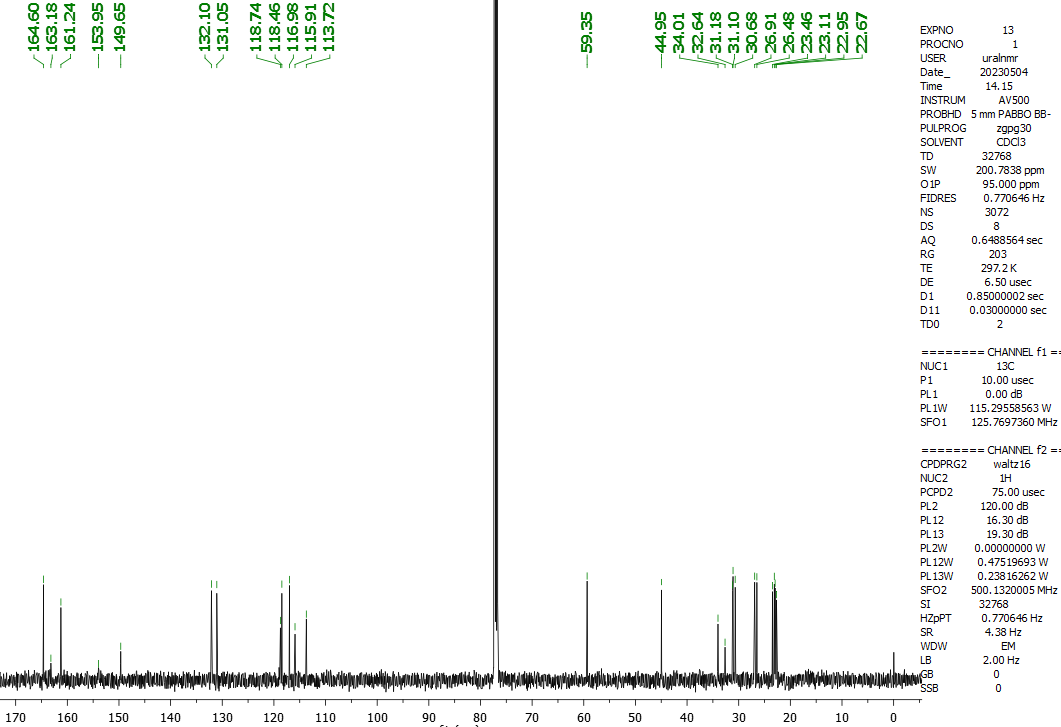


# Figure S28. IR spectrum of 2-[({6-[(2,3,5,6,7,8-hexahydro-1*H*-cyclopenta[*b*]quinolin-9-yl)amino]hexyl}imino)methyl]phenol (9b)

**
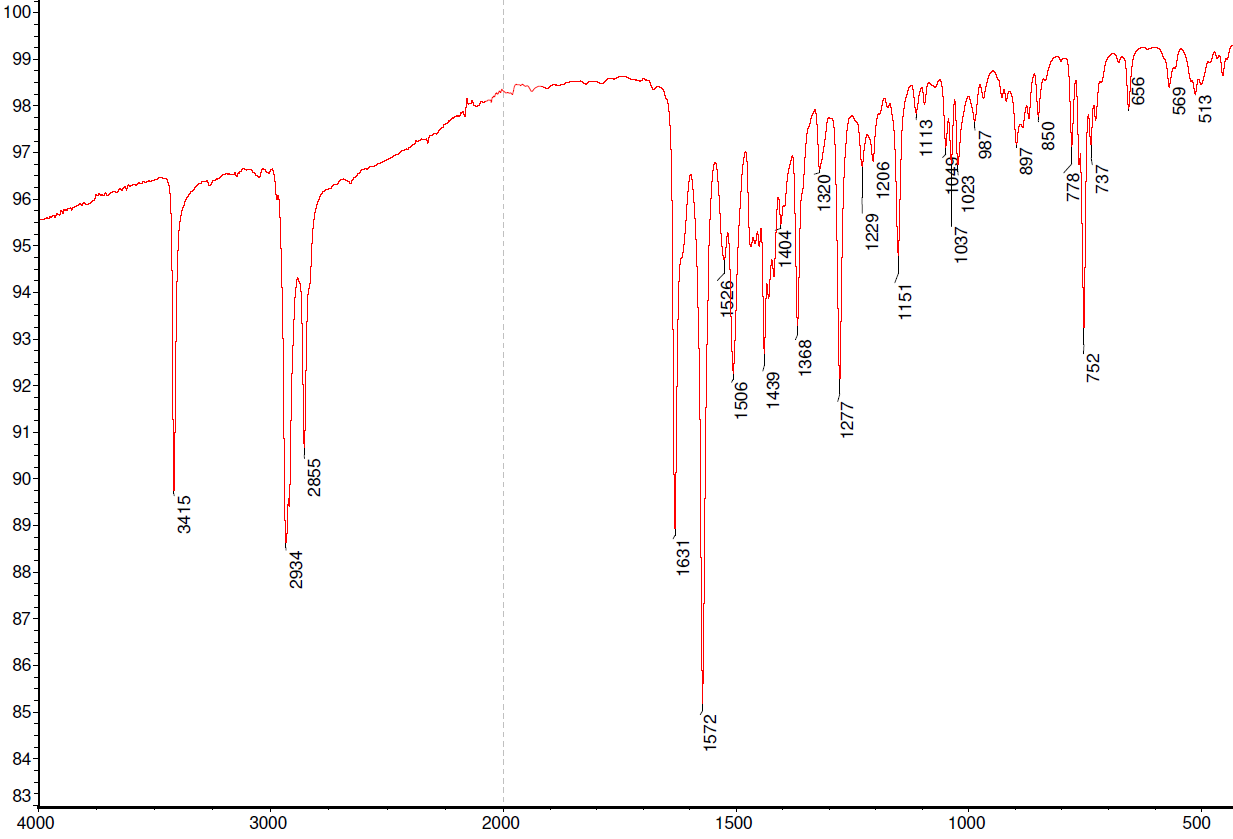
**

# Figure S29. ^1^H NMR spectrum of 2-[({8-[(2,3,5,6,7,8-Hexahydro-1*H*-cyclopenta[*b*]quinolin-9-yl)amino]octyl}imino)methyl]phenol (9c)


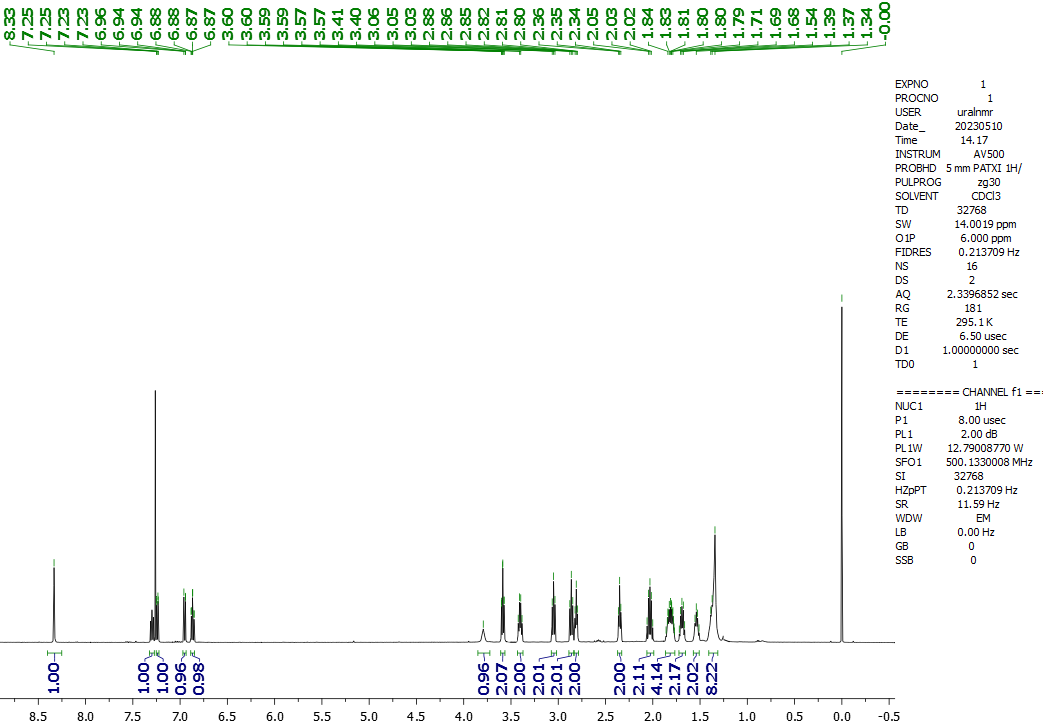


# Figure S30. ^13^C NMR spectrum of 2-[({8-[(2,3,5,6,7,8-Hexahydro-1*H*-cyclopenta[*b*]quinolin-9-yl)amino]octyl}imino)methyl]phenol (9c)


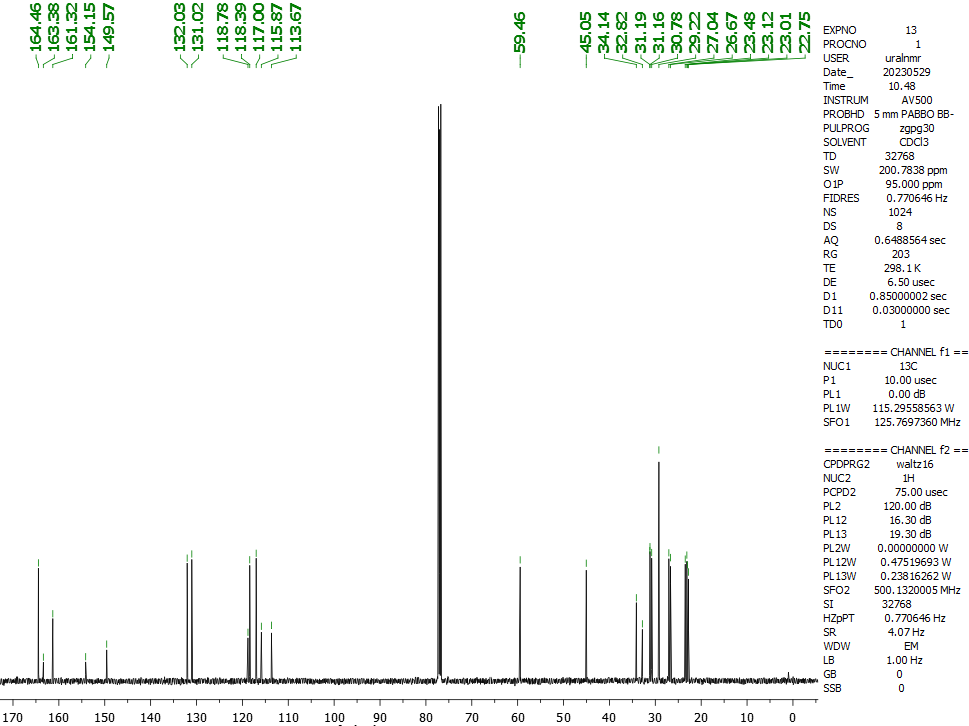

# Figure S31. IR spectrum of 2-[({8-[(2,3,5,6,7,8-Hexahydro-1*H*-cyclopenta[*b*]quinolin-9-yl)amino]octyl}imino)methyl]phenol (9c)

**
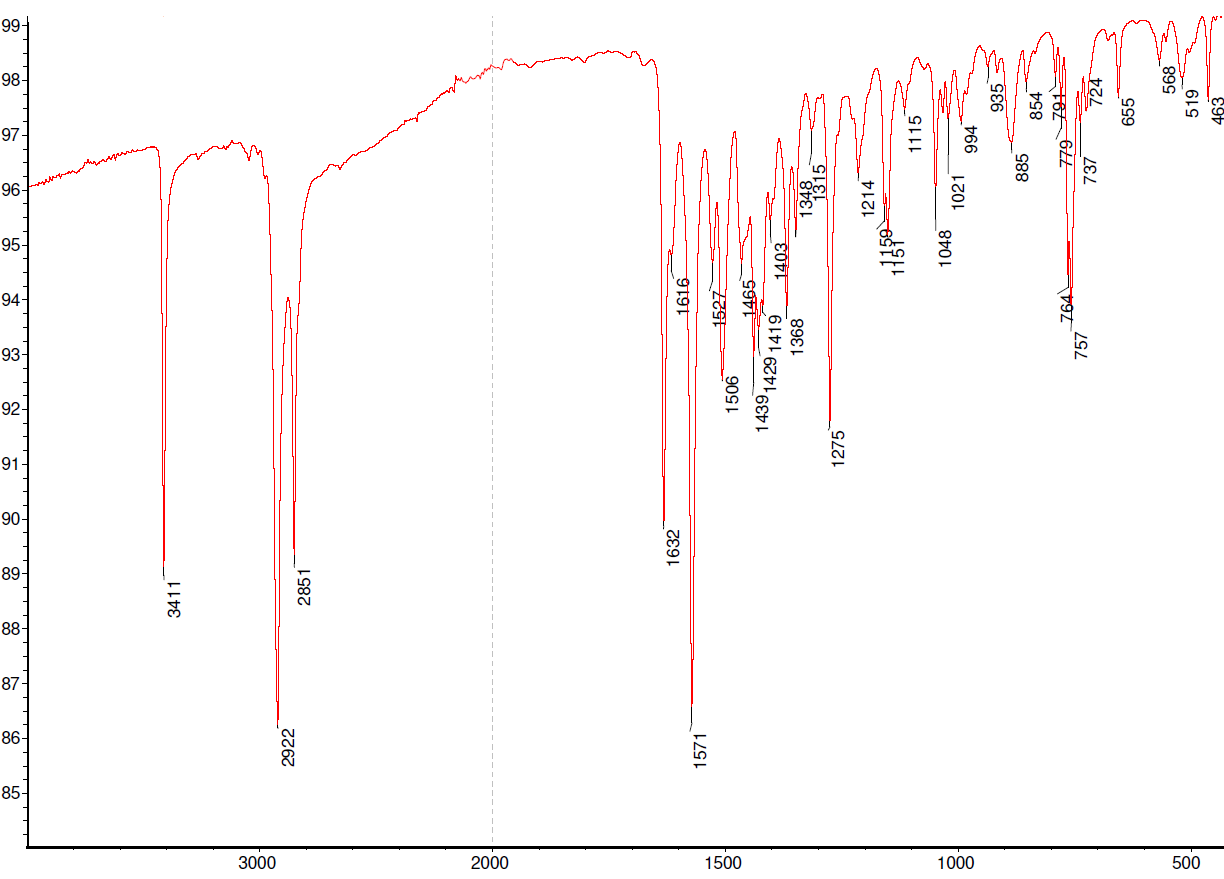
**

# Figure S32. ^1^H NMR spectrum of 2-[({4-[(2,3,5,6,7,8-Hexahydro-1*H*-cyclopenta[*b*]quinolin-9-yl)amino]butyl}amino)methyl]phenol (10a)


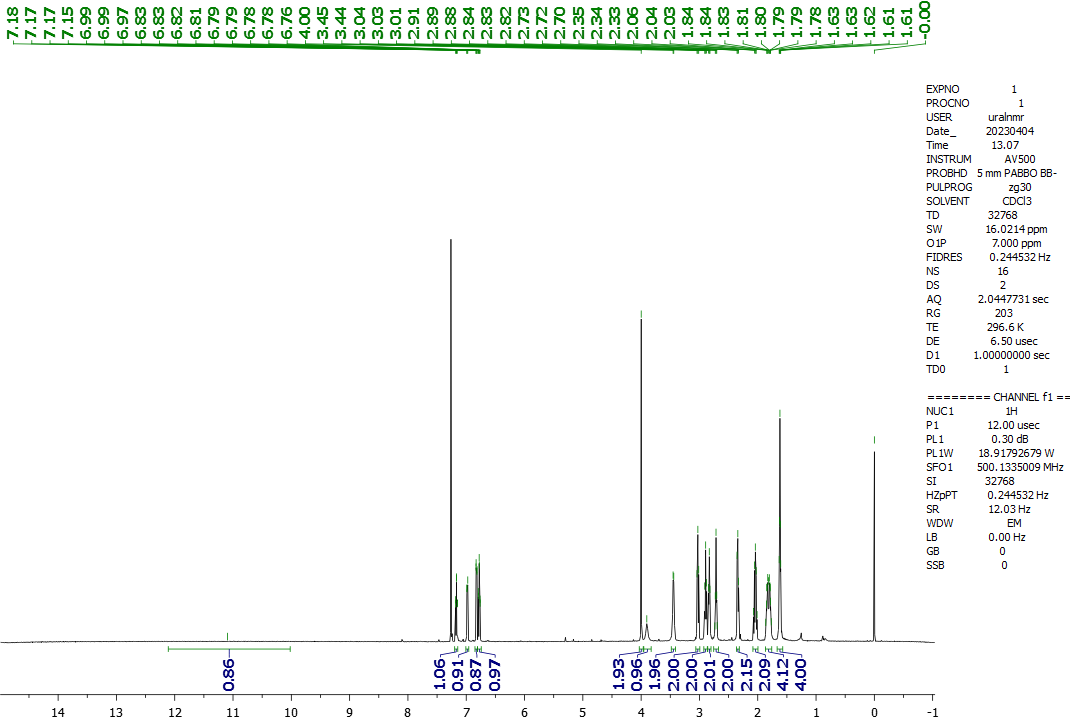


# Figure S33. ^13^C NMR spectrum of 2-[({4-[(2,3,5,6,7,8-Hexahydro-1*H*-cyclopenta[*b*]quinolin-9-yl)amino]butyl}amino)methyl]phenol (10a)


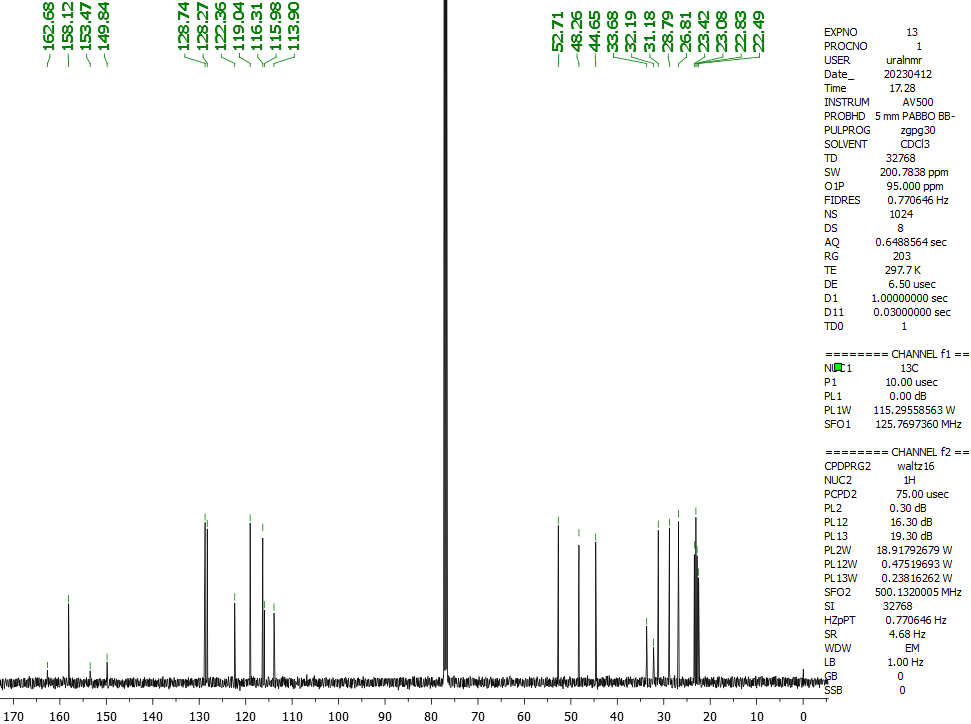


# Figure S34. IR spectrum of 2-[({4-[(2,3,5,6,7,8-Hexahydro-1*H*-cyclopenta[*b*]quinolin-9-yl)amino]butyl}amino)methyl]phenol (10a)

**
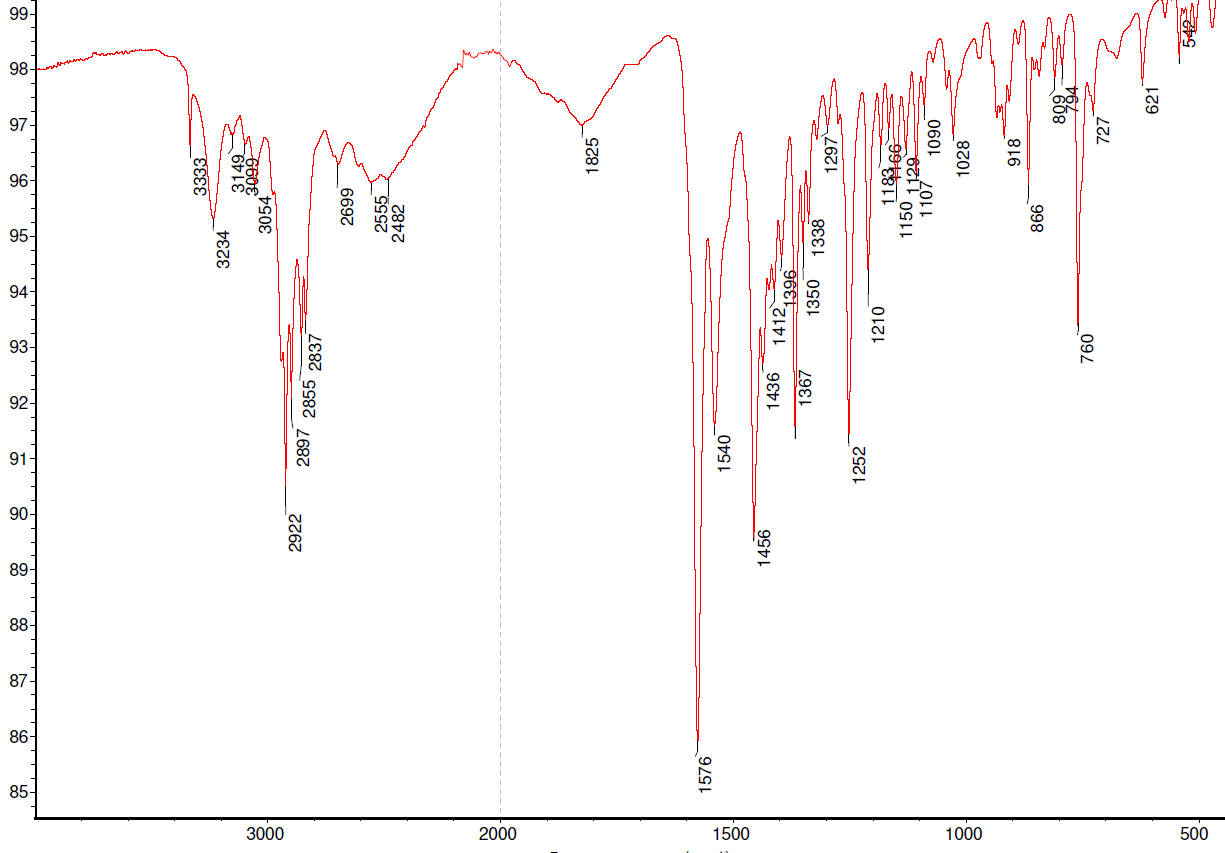
**

# Figure S35. ^1^H NMR spectrum of 2-[({6-[(2,3,5,6,7,8-Hexahydro-1*H*-cyclopenta[*b*]quinolin-9-yl)amino]hexyl}amino)methyl]phenol (10b)


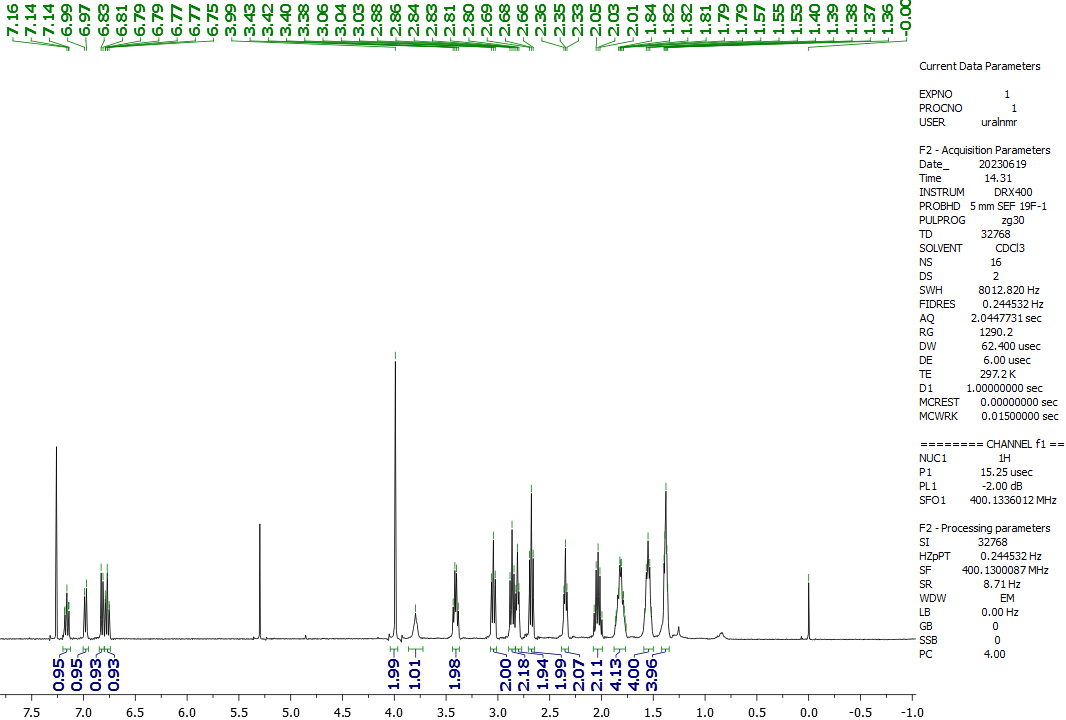


# Figure S36. ^13^C NMR spectrum of 2-[({6-[(2,3,5,6,7,8-Hexahydro-1*H*-cyclopenta[*b*]quinolin-9-yl)amino]hexyl}amino)methyl]phenol (10b)


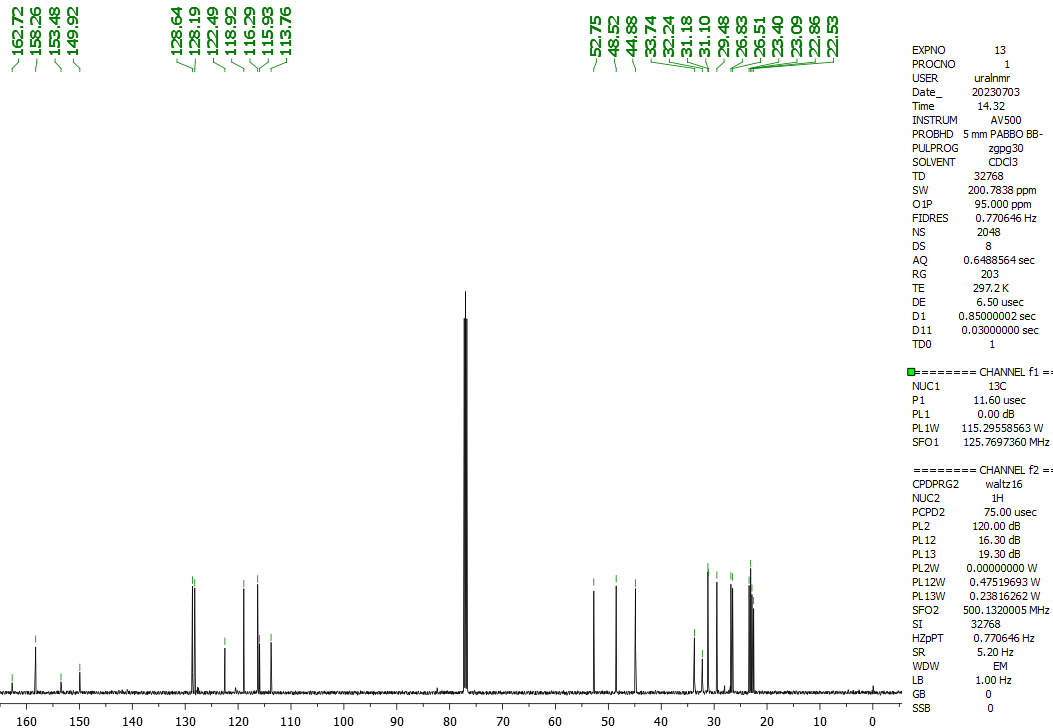


# Figure S37. IR spectrum of 2-[({6-[(2,3,5,6,7,8-Hexahydro-1*H*-cyclopenta[*b*]quinolin-9-yl)amino]hexyl}amino)methyl]phenol (10b)

**
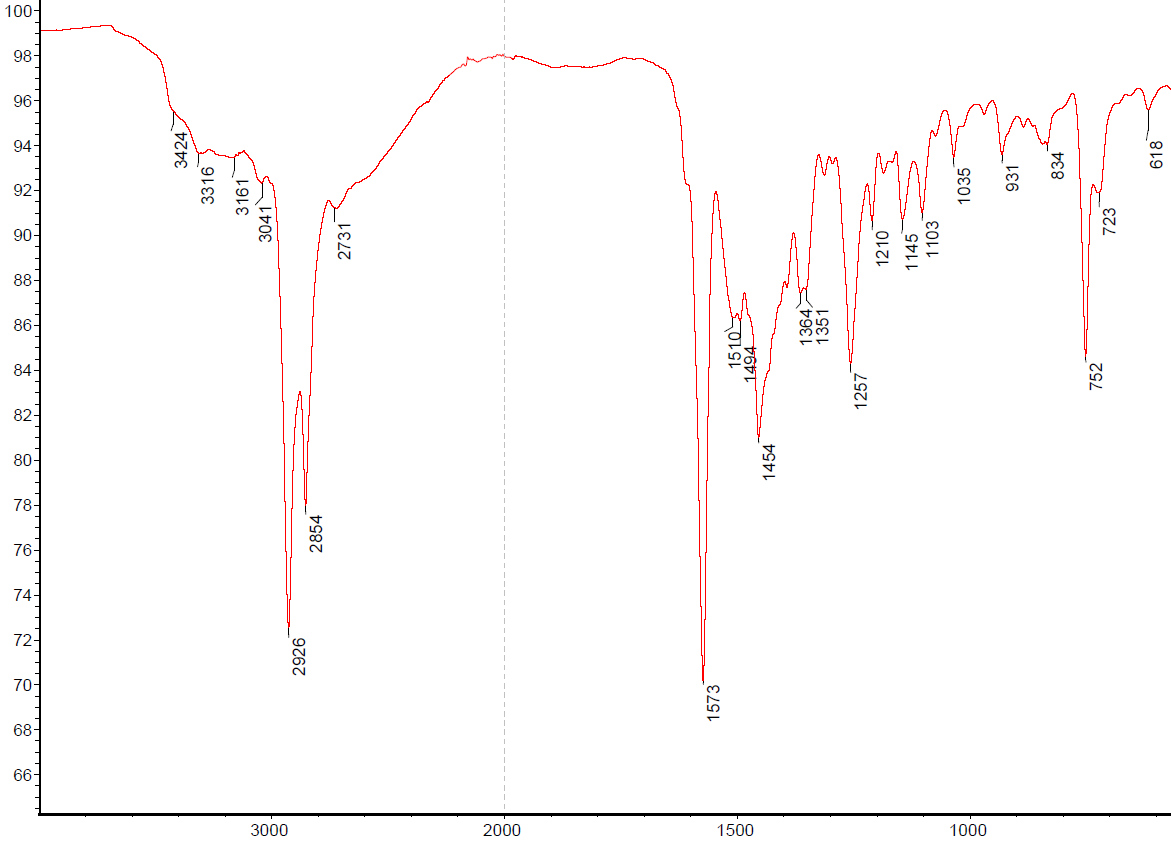
**

# Figure S38. ^1^H NMR spectrum of 2-[({8-[(2,3,5,6,7,8-Hexahydro-1*H*-cyclopenta[*b*]quinolin-9-yl)amino]octyl}amino)methyl]phenol (10c)


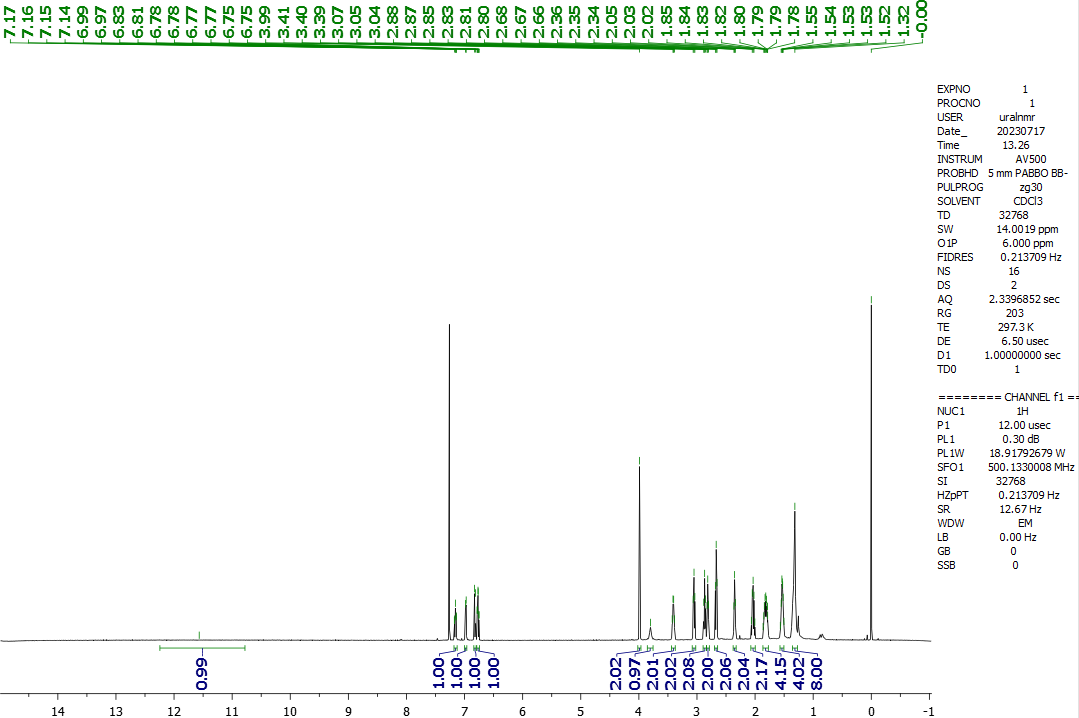


# Figure S39. ^13^C NMR spectrum of 2-[({8-[(2,3,5,6,7,8-Hexahydro-1*H*-cyclopenta[*b*]quinolin-9-yl)amino]octyl}amino)methyl]phenol (10c)


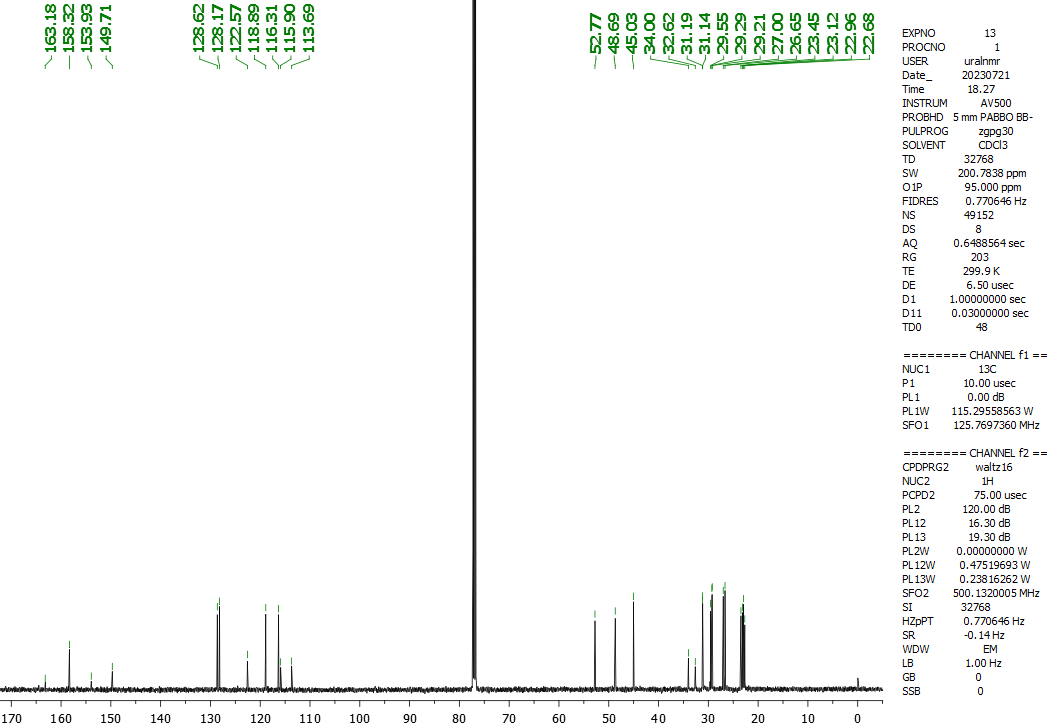


# Figure S40. IR spectrum of 2-[({8-[(2,3,5,6,7,8-Hexahydro-1*H*-cyclopenta[*b*]quinolin-9-yl)amino]octyl}amino)methyl]phenol (10c)

**
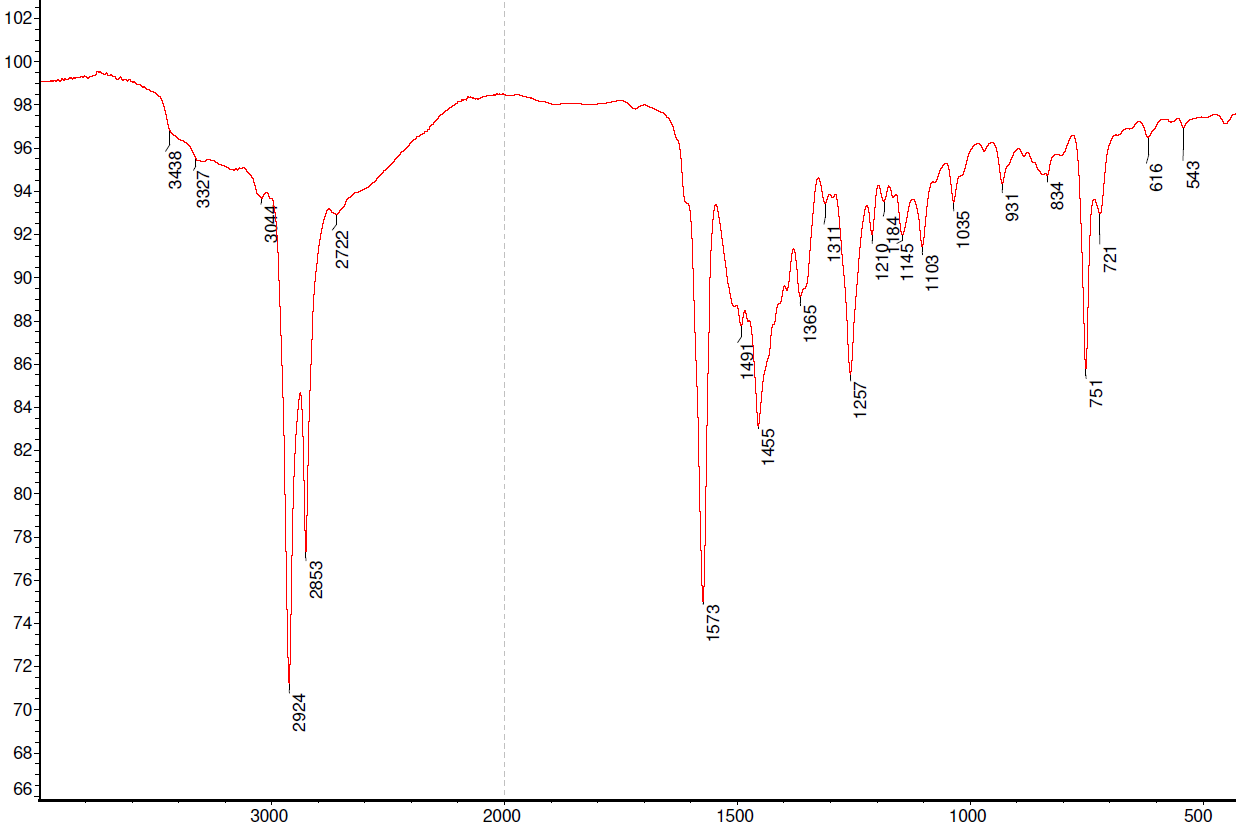
**

# Figure S41. ^1^H NMR spectrum of *N*-hexyl-2,3,5,6,7,8-hexahydro-1*H*-cyclopenta[*b*]quinolin-9-amine (11)


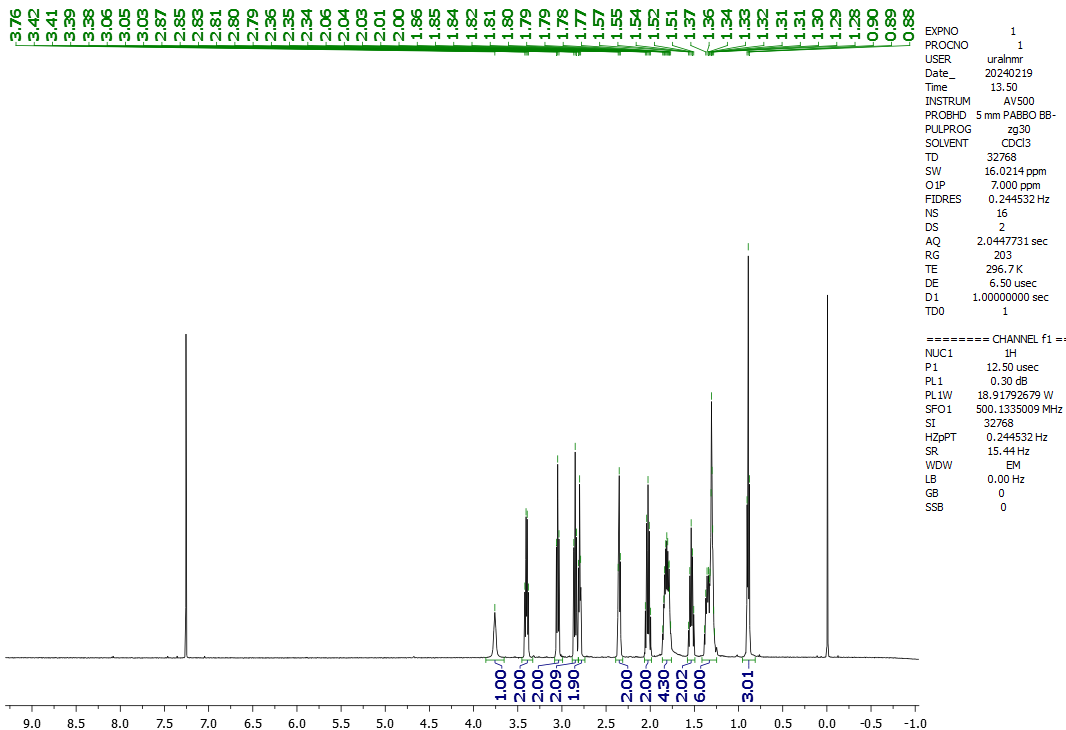


# Figure S42. ^1^C NMR spectrum of *N*-hexyl-2,3,5,6,7,8-hexahydro-1*H*-cyclopenta[*b*]quinolin-9-amine (11)


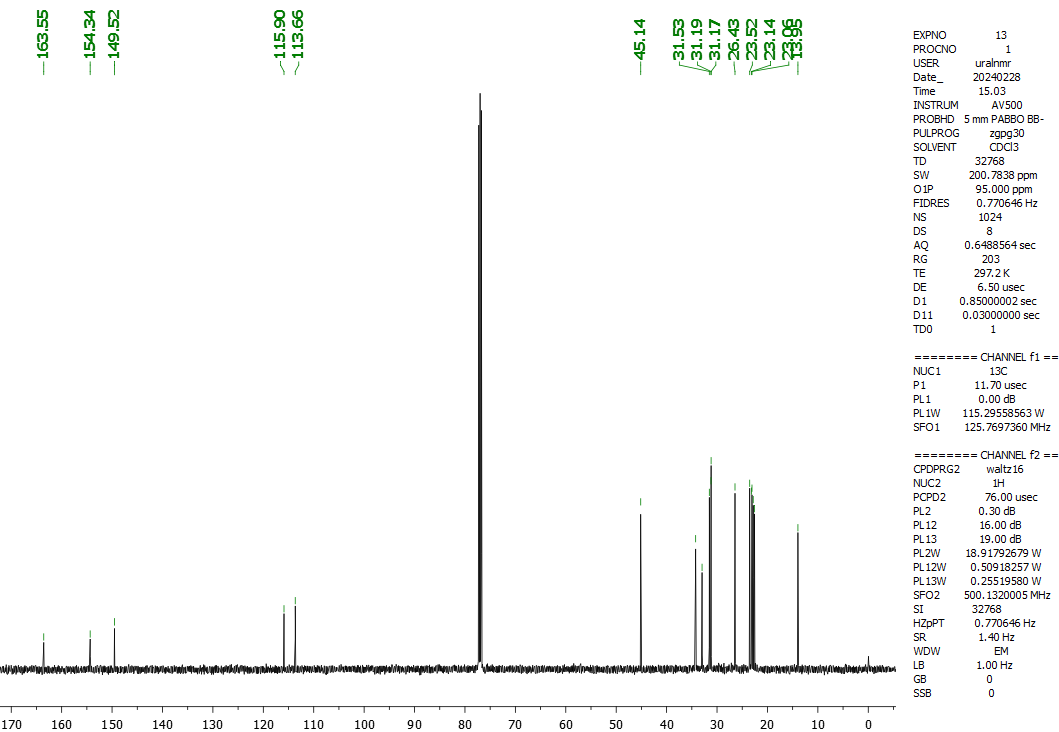


# Figure S43. IR spectrum of *N*-hexyl-2,3,5,6,7,8-hexahydro-1*H*-cyclopenta[*b*]quinolin-9-amine (11)


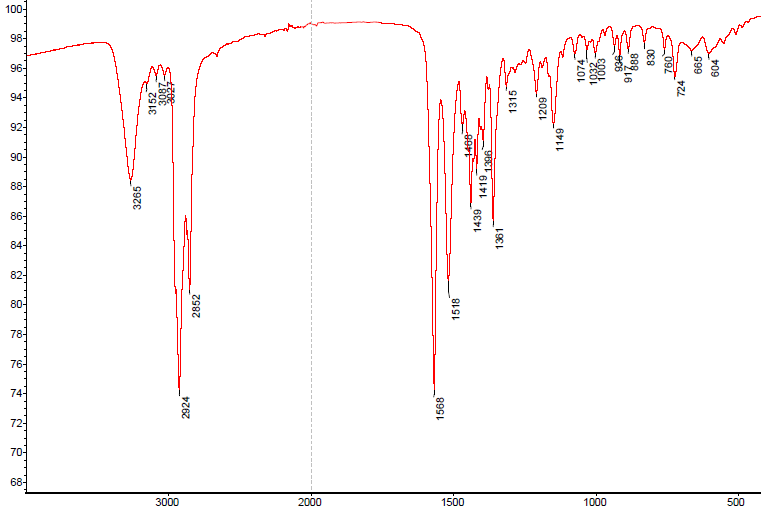


# Figure S44. IR spectrum of 2-[(hexylimino)methyl]phenol (12)


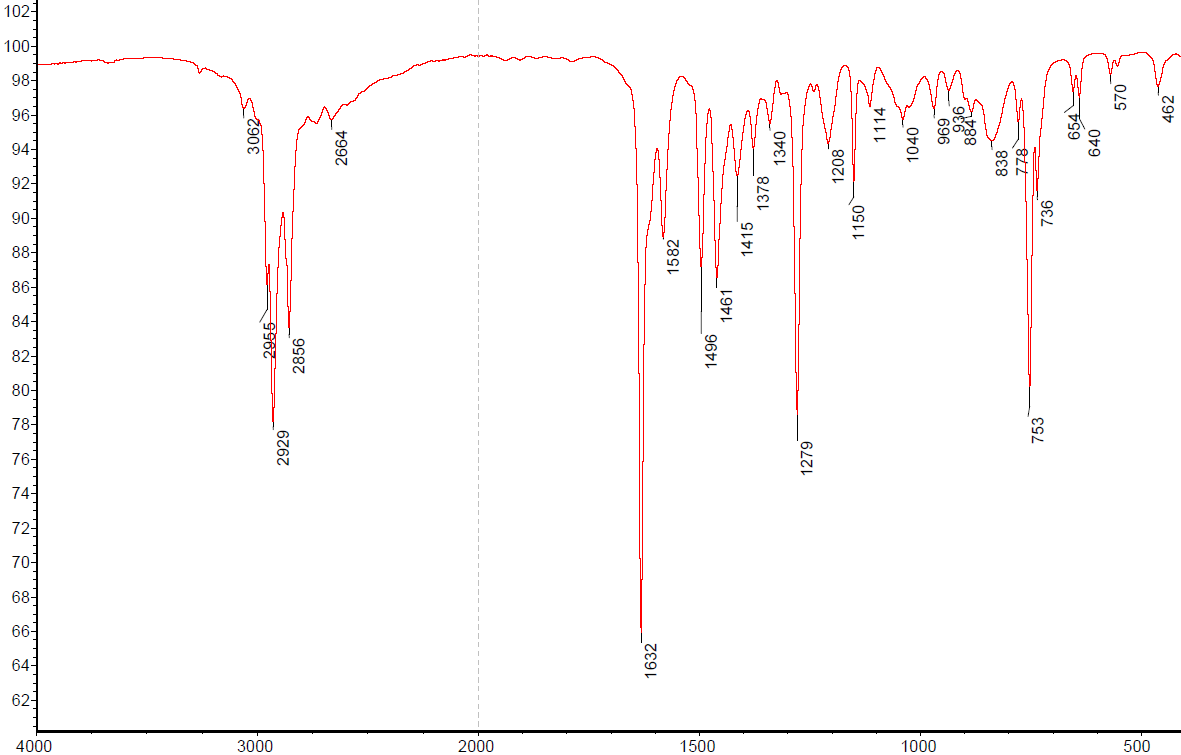


# Figure S45. ^1^H NMR spectrum of 2-[(hexylamino)methyl]phenol (13)


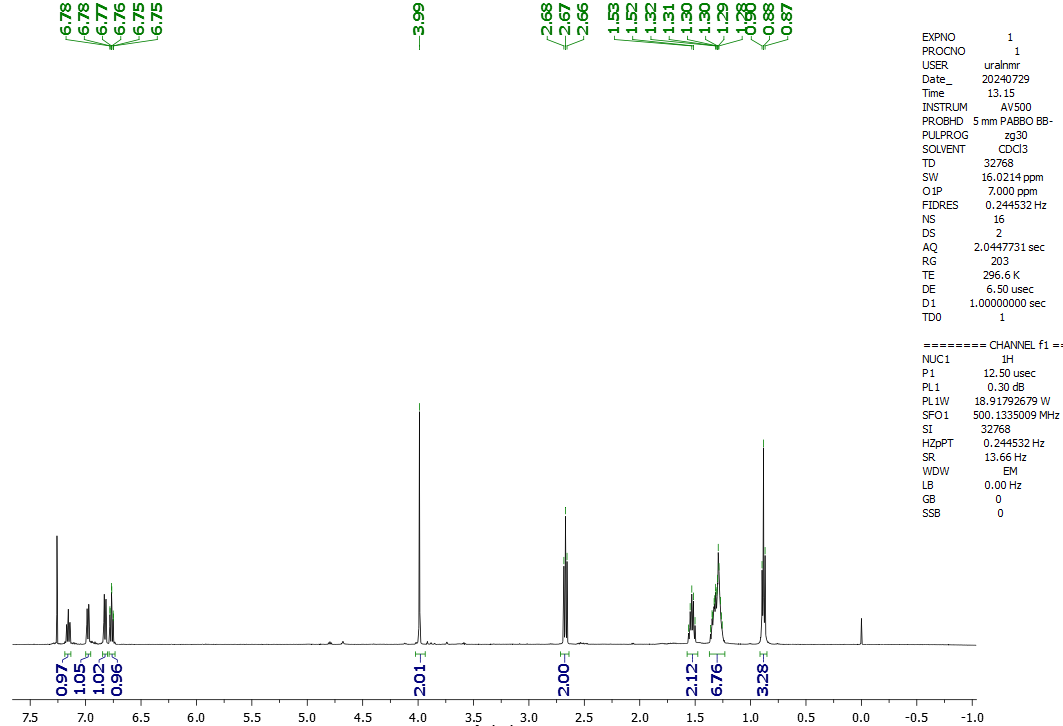


# Figure S46. ^1^C NMR spectrum of 2-[(hexylamino)methyl]phenol (13)


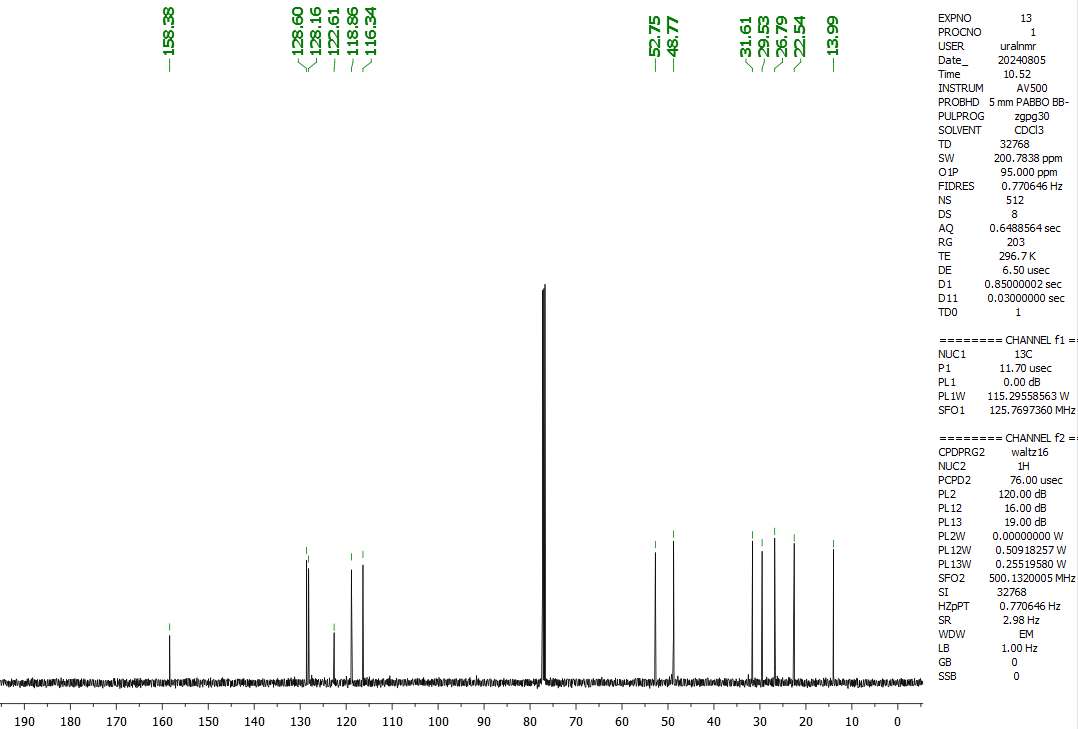

# Figure S47. IR spectrum of 2-[(hexylamino)methyl]phenol (13)


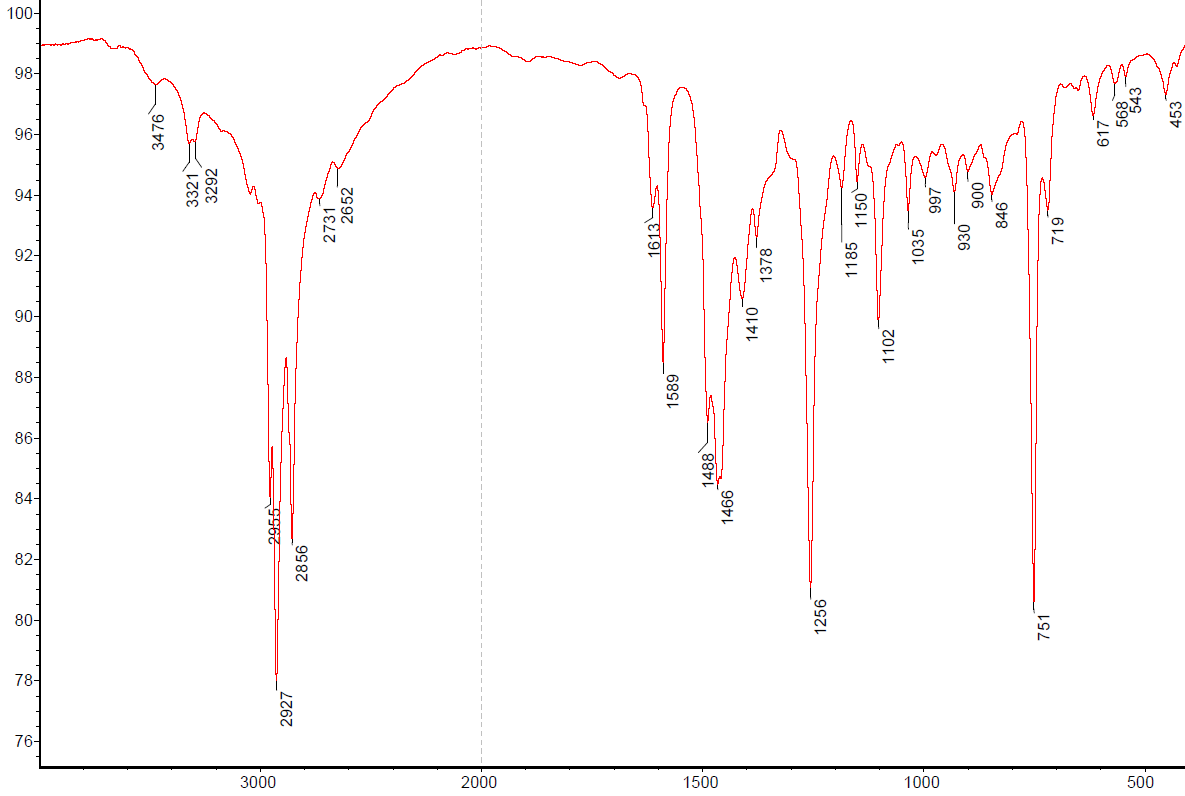


# Figure S48. (a) UV spectra of compound 7c and mixtures of 7c with Cu^2+^, Fe^2+^, and Zn^2+^ ions. (b) UV spectra of compound 9c and mixtures of 9c with Cu^2+^, Fe^2+^, and Zn^2+^ ions

| 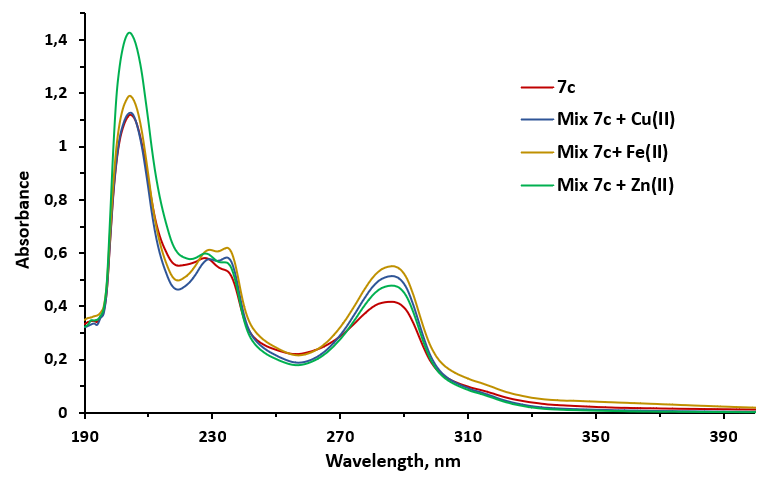 | 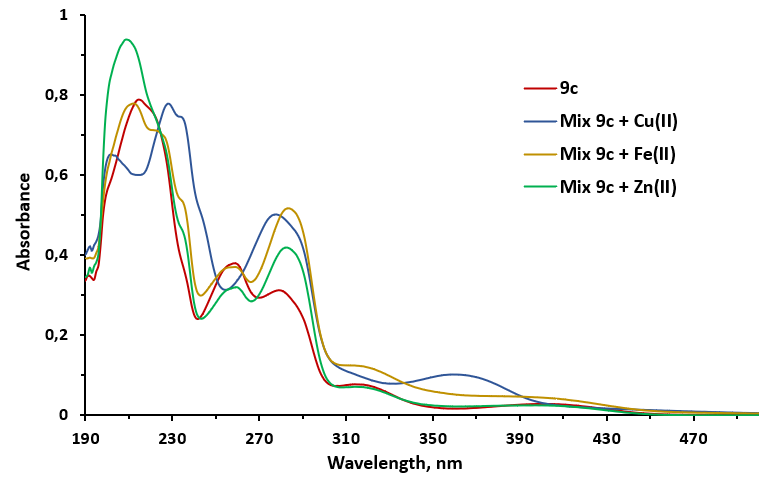 |
| --- | --- |
| **a** | **b** |

# Figure S49. (a) UV spectra of compound 11 and mixtures of 11 with Cu^2+^, Fe^2+^, and Zn^2+^ ions. (b) UV spectra of compound 12 and mixtures of 12 with Cu^2+^, Fe^2+^, and Zn^2+^ ions

| 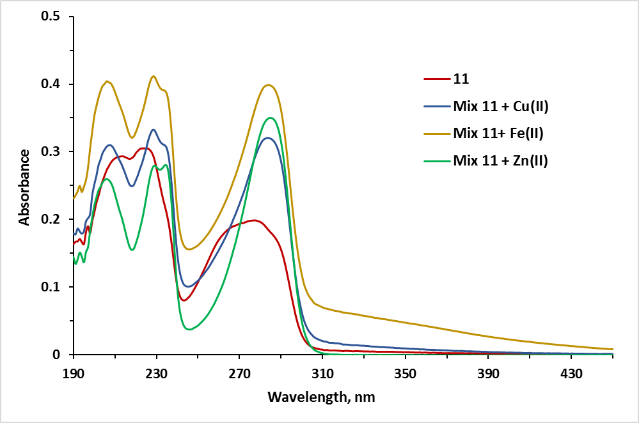 | 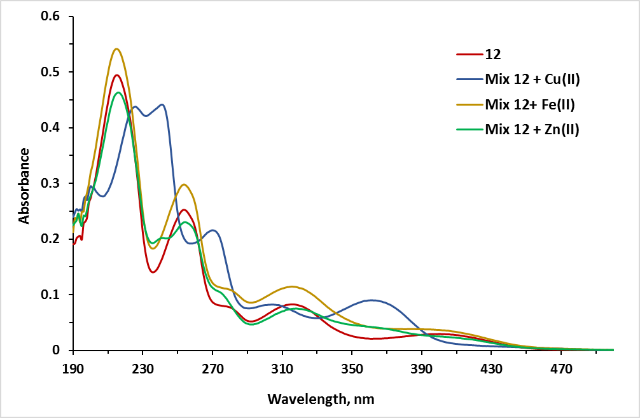 |
| --- | --- |
| **а** | **b** |

# Figure S50. (a) UV spectra of the 7c–Cu^2+^, 14–Cu^2+^, 11–Cu^2+^ complexes, obtained subtracting the spectra of ions and compounds from the spectra of mixtures. (b) UV spectra of the 10c–Cu^2+^, 13–Cu^2+^, 11–Cu^2+^ complexes, obtained subtracting the spectra of ions and compounds from the spectra of mixtures.

| 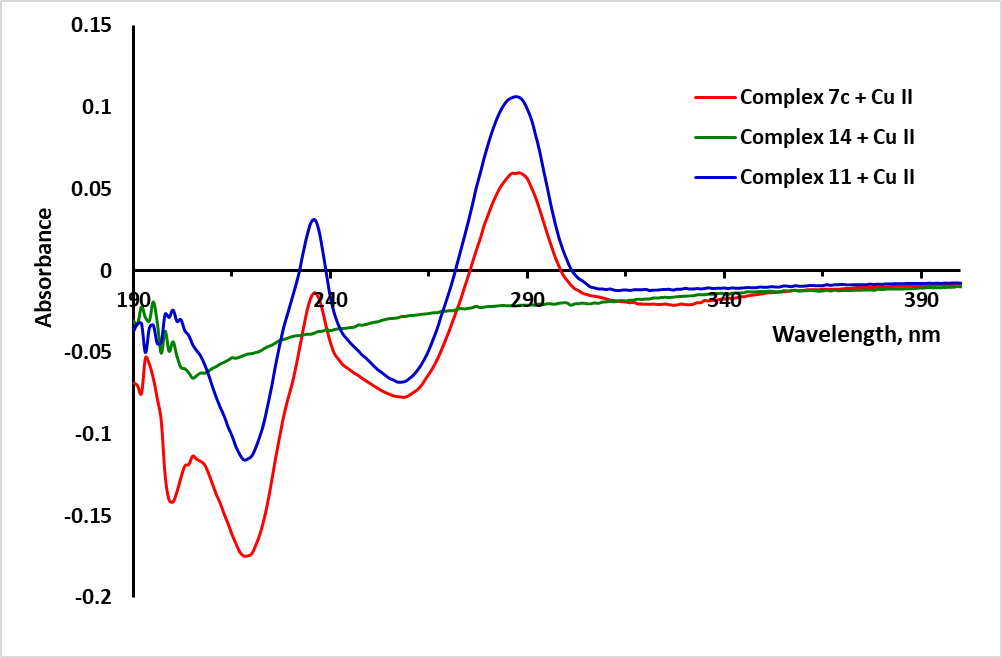 | 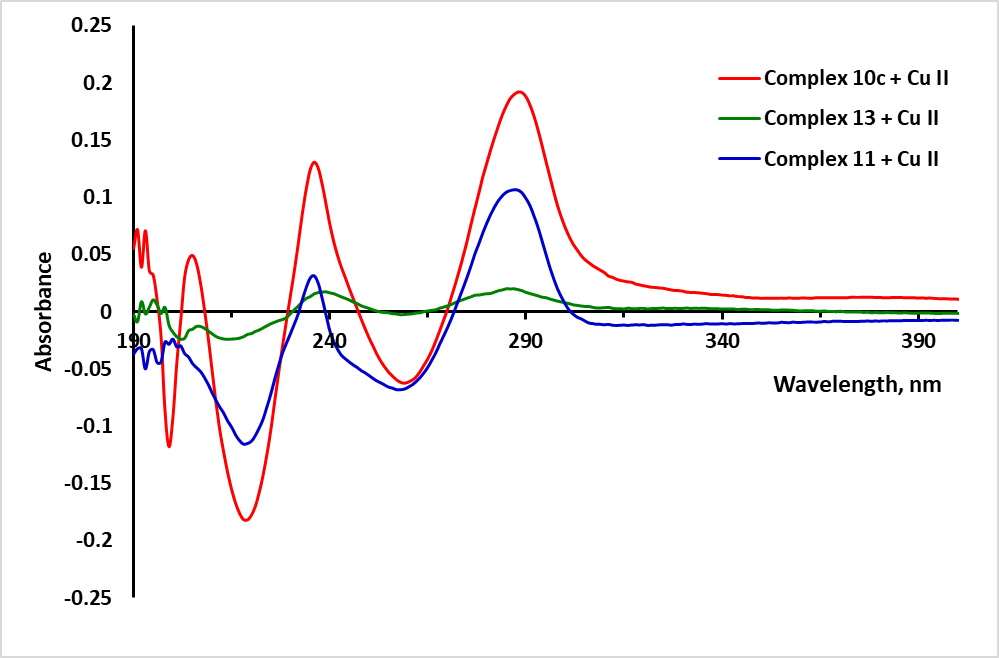 |
| --- | --- |
| **a** | **b** |

# Figure S51. (a) UV absorption spectra of 7c (20 µM) in EtOH after addition of increasing concentrations of CuCl_2_ (2–34 µM). (b) the differential spectra due to the 7c–Cu^2+^ complex formation obtained by numerical subtraction from the spectra of the mixture of the spectra of the Cu^2+^ alone and 7c alone at the corresponding concentrations.

| 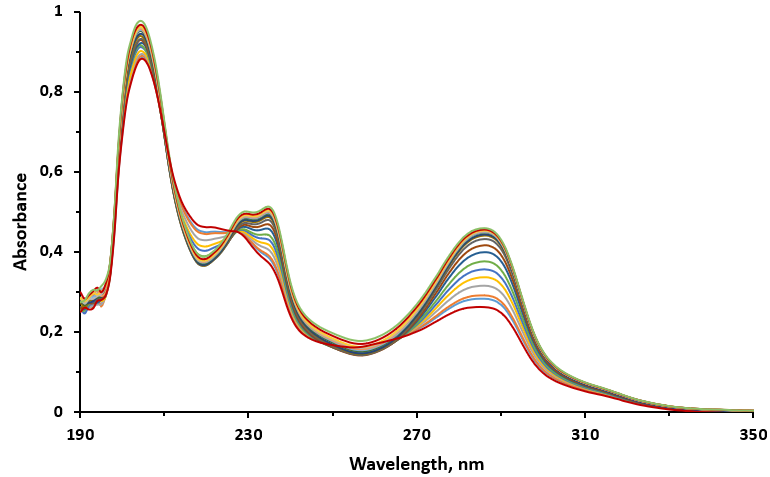 | 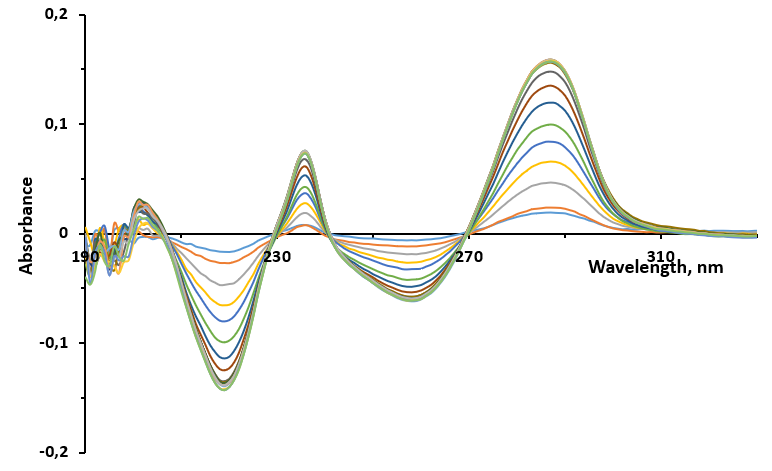 |
| --- | --- |
| **a** | **b** |

# Figure S52. (a) UV absorption spectra of 9c (20 µM) in EtOH after addition of increasing concentrations of CuCl_2_ (2–34 µM). (b) the differential spectra due to the 9c–Cu^2+^ complex formation obtained by numerical subtraction from the spectra of the mixture of the spectra of the Cu^2+^ alone and 9c alone at the corresponding concentrations.

| 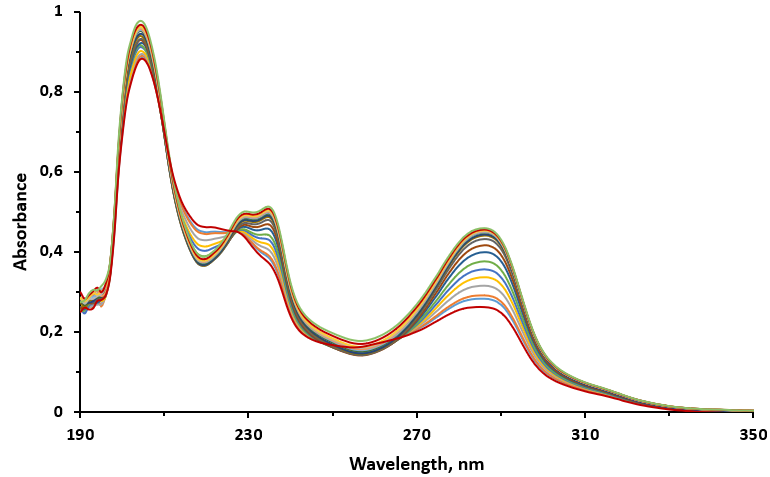 | 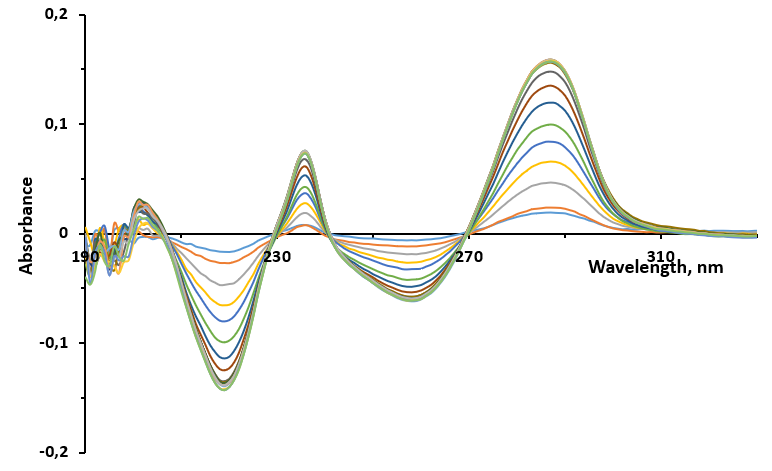 |
| --- | --- |
| **a** | **b** |

# Figure S53. (a) UV absorption spectra of 10c (20 µM) in EtOH after addition of increasing concentrations of CuCl_2_ (2–34 µM). (b) the differential spectra due to the 10c–Cu^2+^ complex formation obtained by numerical subtraction from the spectra of the mixture of the spectra of the Cu^2+^ alone and 10c alone at the corresponding concentrations.

| 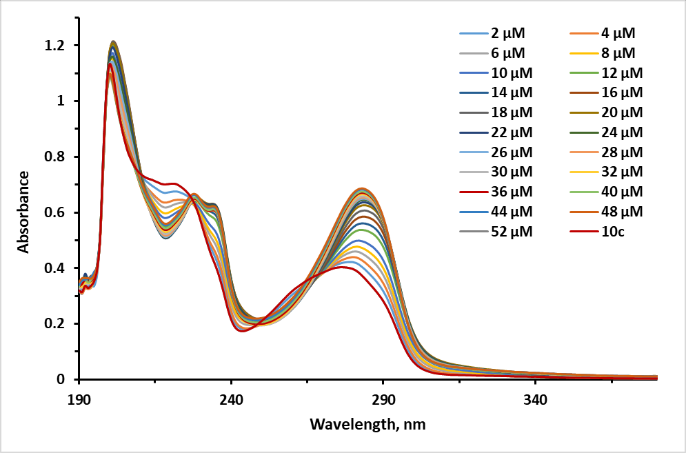 | 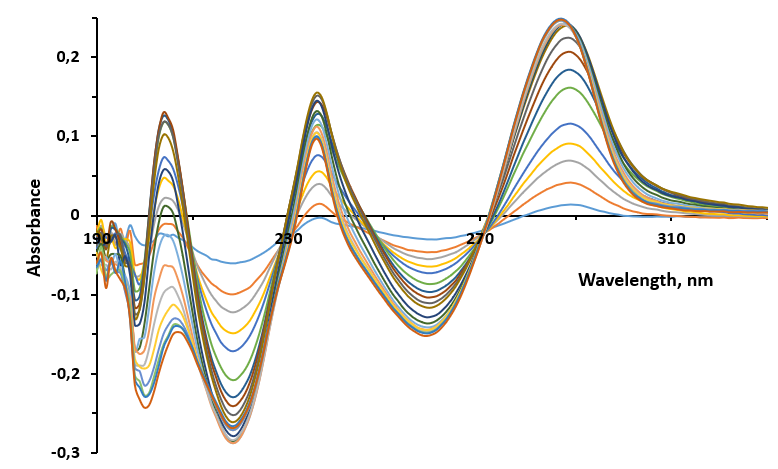 |
| --- | --- |
| **a** | **b** |

# Figure S54. (a) Absorbance of the 7c–Cu^2+^ complex at 352 nm as a function of the concentration of Cu^2+^ (compounds concentration is 20 µM). Vertical dashed lines mark the metal concentration at the breakpoints and indicate ligand–metal molar ratios of 1.05:1. (b) Absorbance of the 9c–Cu^2+^ complex at 352 nm as a function of the concentration of Cu^2+^ (compounds concentration is 40 µM). Vertical dashed lines mark the metal concentration at the breakpoints and indicate ligand–metal molar ratios of 1.7:1.

| 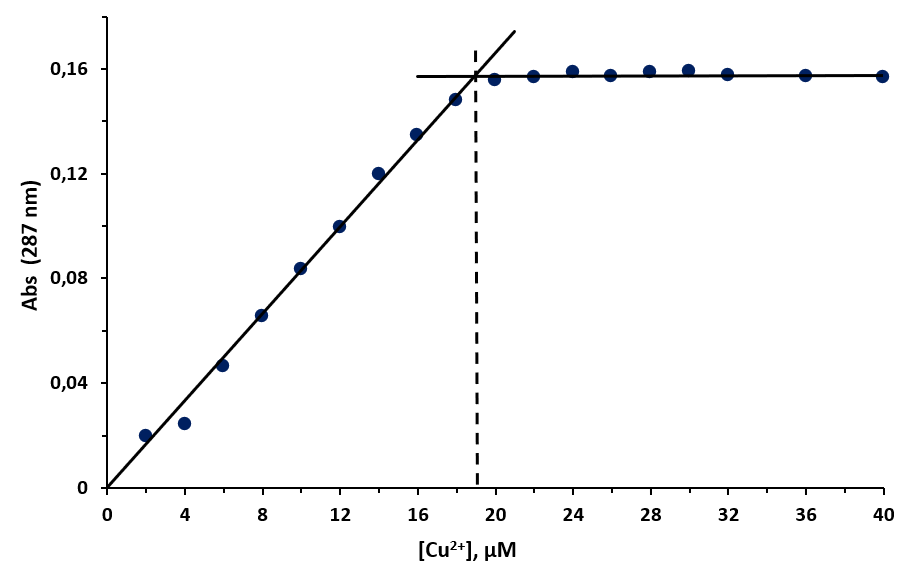 | 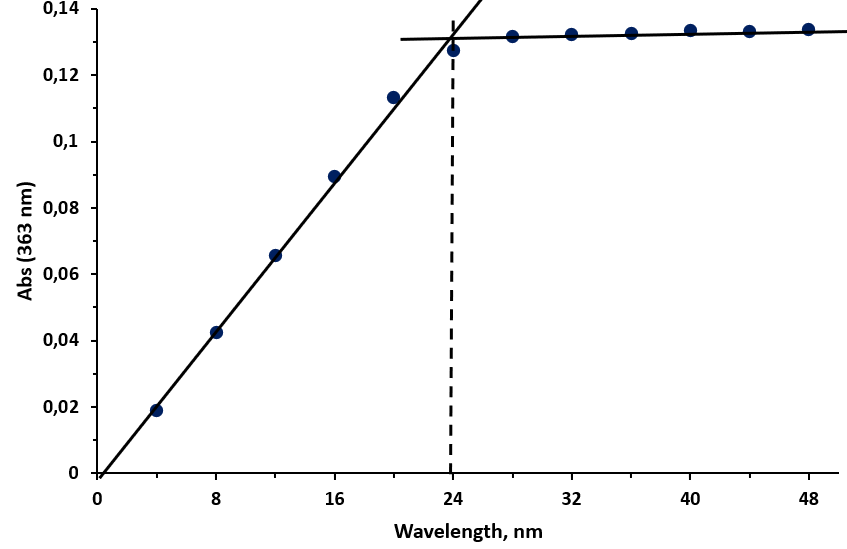 |
| --- | --- |
| **a** | **b** |

# Figure S55. (a) UV absorption spectra of 11 (20 µM) in EtOH after addition of increasing concentrations of CuCl_2_ (2–42 µM). (b) the differential spectra due to the 11–Cu^2+^ complex formation obtained by numerical subtraction from the spectra of the mixture of the spectra of the Cu^2+^ alone and 11 alone at the corresponding concentrations. (с) absorbance of the 11–Cu^2+^ complex at 287 nm as a function of the concentration of Cu^2+^ (compounds concentration is 20 µM). Vertical dashed lines mark the metal concentration at the breakpoints and indicate ligand–metal molar ratios of 0.9:1.
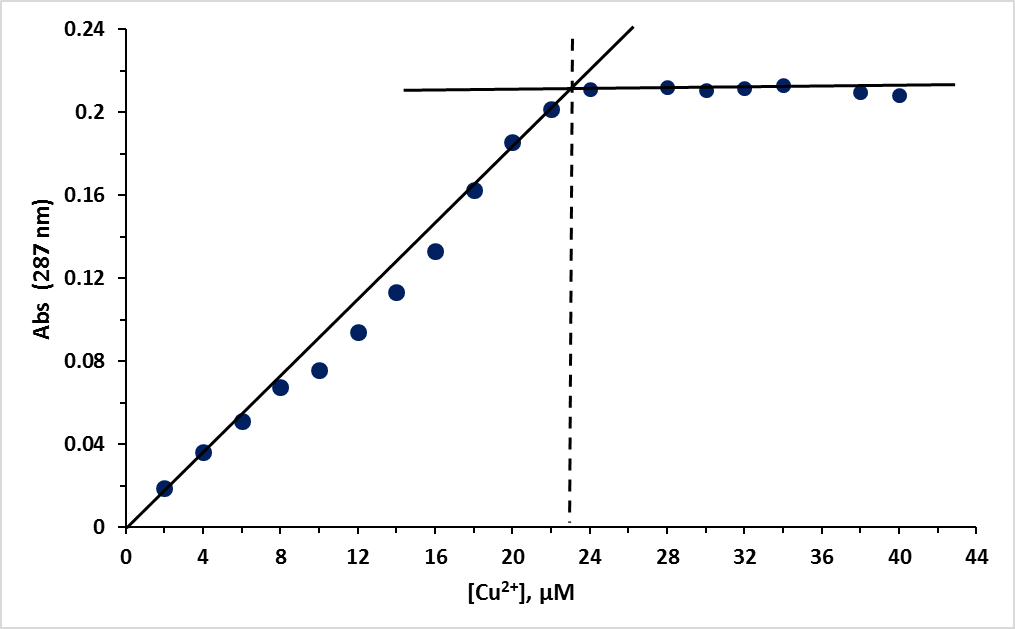


| **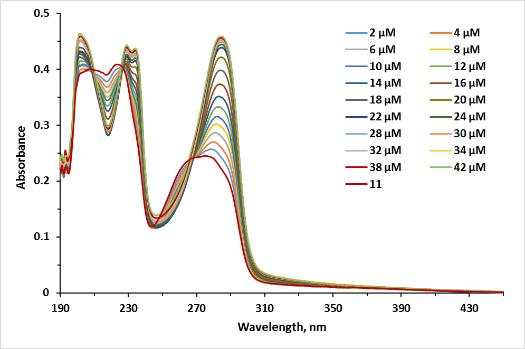a** | 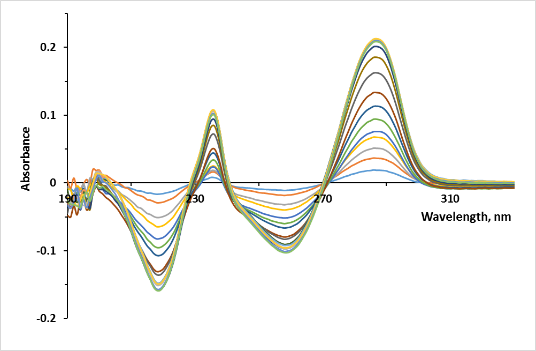**b** |
| --- | --- |
| **c** | |

# Figure S56. Cytotoxic effect (IC_50_, μM; mean ± SEM, *n* = 3) toward HEK293T for conjugates of salicylic derivatives 7a, 9a and 10a.

# Figure S57. Cytotoxic effect (IC_50_, μM; mean ± SEM, *n* = 3) toward SH-SY5Y for conjugates of salicylic derivatives 7a, 9a, and 10a.

# Figure S58. Cytotoxic effect (IC_50_, μM; mean ± SEM, *n* = 3) toward HepG2 for conjugates of salicylic derivatives 7a, 9a, and 10a.

# Figure S59. Cytotoxic effect (IC_50_, μM; mean ± SEM, *n* = 3 ) toward HEK293T for conjugates of amiridine and salicylic derivatives 7c, 9c, and 10c.

# Figure S60. Cytotoxic effect (IC_50_, μM; mean ± SEM, *n* = 3 ) toward SH-SY5Y for conjugates of amiridine and salicylic derivatives 7c, 9c, and 10c.

# Figure S61. Cytotoxic effect (IC_50_, μM; mean ± SEM, *n* = 3) toward HepG2 for conjugates of amiridine and salicylic derivatives 7c, 9c, and 10c.
