# Supplementary material for: Conjugates of amiridine and salicylic derivatives as promising multifunctional CNS agents for potential treatment of Alzheimer's disease
Source: Arch Pharm (Weinheim). 2024 Dec 17;358(1):e2400819. doi: 10.1002/ardp.202400819 (PMC11650361; doi:10.1002/ardp.202400819)
Supplement: Supplementary file 2 — Supporting information. [file ARDP-358-e2400819-s002.doc]

**Supplemental Material: Novel Compounds and Biological Screening Results**

**Conjugates of Amiridine and Salicylic Acid Derivatives as promising multifunctional CNS agents for potential treatment of Alzheimer's disease**

Galina F. Makhaeva^1^, Maria V. Grishchenko^2^, Nadezhda V. Kovaleva^1^, Natalia P. Boltneva^1^, Elena V. Rudakova ^1^, Tatiana Y. Astakhova^1,3^, Elena N. Timokhina^3^, Pavel G. Pronkin^1,3^, Sofya V. Lushchekina^4^, Olga G. Khudina^2^, Ekaterina F. Zhilina^2^, Evgeny V. Shchegolkov^2^, Maria A. Lapshina^1^, Elena S. Dubrovskaya^1^, Eugene V. Radchenko^1,5^, Vladimir A. Palyulin^1,5^, Yanina V. Burgart^2^, Victor I. Saloutin ^2^, Valery N. Charushin^2^, Rudy J. Richardson^6,7.8,9^,*

^1^ Institute of Physiologically Active Compounds at Federal Research Center of Problems of Chemical Physics and Medicinal Chemistry, Russian Academy of Sciences, Chernogolovka 142432, Russia

^2^Postovsky Institute of Organic Synthesis, Urals Branch of the Russian Academy of Sciences, Ekaterinburg 620990, Russia

^3^Emanuel Institute of Biochemical Physics, Russian Academy of Sciences, Moscow 119334, Russia

^4^Department of Brain Sciences, Weizmann Institute of Science, Rehovot 761001, Israel

^5^Department of Chemistry, Lomonosov Moscow State University, Moscow 119991, Russia

^6^Department of Environmental Health Sciences, University of Michigan, Ann Arbor, MI 48109, USA

^7^Department of Neurology, University of Michigan, Ann Arbor, MI 48109, USA

^8^Center of Computational Medicine and Bioinformatics, University of Michigan, Ann Arbor, MI 48109, USA

^9^Michigan Institute for Computational Discovery and Engineering, University of Michigan, Ann Arbor, MI 48109, USA

^*^Corresponding author – Rudy J. Richardson, Molecular Simulations Laboratory, Room M6065 SPH-II 2029, University of Michigan, 1415 Washington Heights

Ann Arbor, Michigan 48109-2029 USA

Email: rjrich@umich.edu

| **Compound No.** | **InChI** | **Inhibitory activity against AChE, BChE and CES^a^** | | | **Propidium displace-ment, %^b^** | **Inhibition of Aβ_42_ self-**  **aggrega-tion, %^c^** |
| --- | --- | --- | --- | --- | --- | --- |
|  |  | **AChE, IC_50_, µM** | **BChE, IC_50_, µM** | **CES, %** |  |  |
| **7a** | InChI=1S/C23H29N3O2/c27-21-13-4-2-9-18(21)23(28)25-15-6-5-14-24-22-16-8-1-3-11-19(16)26-20-12-7-10-17(20)22/h2,4,9,13,27H,1,3,5-8,10-12,14-15H2,(H,24,26)(H,25,28) | 1.52±0.14 | 0.254±0.023 | 5.0±0.1 | 10.8±0.8 | 41.3±3.3 |
| **7b** | InChI=1S/C25H33N3O2/c29-23-15-6-4-11-20(23)25(30)27-17-8-2-1-7-16-26-24-18-10-3-5-13-21(18)28-22-14-9-12-19(22)24/h4,6,11,15,29H,1-3,5,7-10,12-14,16-17H2,(H,26,28)(H,27,30) | 4.24±0.08 | 0.022±0.001 | 12.3±1.2 | 11.6±0.9 | 53.4±4.2 |
| **7c** | InChI=1S/C27H37N3O2/c31-25-17-8-6-13-22(25)27(32)29-19-10-4-2-1-3-9-18-28-26-20-12-5-7-15-23(20)30-24-16-11-14-21(24)26/h6,8,13,17,31H,1-5,7,9-12,14-16,18-19H2,(H,28,30)(H,29,32) | 1.53±0.03 | 0.0111±0.0001 | 32.9±0.9 | 11.8±0.8 | 74.1±5.1 |
| **9a** | InChI=1S/C23H29N3O/c27-22-13-4-1-8-17(22)16-24-14-5-6-15-25-23-18-9-2-3-11-20(18)26-21-12-7-10-19(21)23/h1,4,8,13,16,27H,2-3,5-7,9-12,14-15H2,(H,25,26)/b24-16+ | 3.31±0.02 | 0.642±0.007 | 10.5±0.9 | 12.5±1.1 | 20.1±1.4 |
| **9b** | InChI=1S/C25H33N3O/c29-24-15-6-3-10-19(24)18-26-16-7-1-2-8-17-27-25-20-11-4-5-13-22(20)28-23-14-9-12-21(23)25/h3,6,10,15,18,29H,1-2,4-5,7-9,11-14,16-17H2,(H,27,28)/b26-18+ | 3.25±0.08 | 0.062±0.006 | 12.4±1.2 | 11.7±0.8 | 59.1±4.7 |
| **9c** | InChI=1S/C27H37N3O/c31-26-17-8-5-12-21(26)20-28-18-9-3-1-2-4-10-19-29-27-22-13-6-7-15-24(22)30-25-16-11-14-23(25)27/h5,8,12,17,20,31H,1-4,6-7,9-11,13-16,18-19H2,(H,29,30)/b28-20+ | 1.42±0.14 | 0.025±0.001 | 25.8±0.7 | 11.6±0.9 | 71.7±5.1 |
| **10a** | InChI=1S/C23H31N3O/c27-22-13-4-1-8-17(22)16-24-14-5-6-15-25-23-18-9-2-3-11-20(18)26-21-12-7-10-19(21)23/h1,4,8,13,24,27H,2-3,5-7,9-12,14-16H2,(H,25,26) | 2.40±0.15 | 0.198±0.019 | 6.2±0.5 | 13.6±0.9 | 30.5±2.4 |
| **10b** | InChI=1S/C25H35N3O/c29-24-15-6-3-10-19(24)18-26-16-7-1-2-8-17-27-25-20-11-4-5-13-22(20)28-23-14-9-12-21(23)25/h3,6,10,15,26,29H,1-2,4-5,7-9,11-14,16-18H2,(H,27,28) | 1.19±0.05 | 0.0294±0.0006 | 18.6±0.1 | 15.1±1.2 | 79.2±5.5 |
| **10c** | InChI=1S/C27H39N3O/c31-26-17-8-5-12-21(26)20-28-18-9-3-1-2-4-10-19-29-27-22-13-6-7-15-24(22)30-25-16-11-14-23(25)27/h5,8,12,17,28,31H,1-4,6-7,9-11,13-16,18-20H2,(H,29,30) | 0.265±0.018 | 0.0220±0.0002 | 56.3±0.3  (IC_50_= 10.5±0.1 µM) | 15.8±1.1 | 96.7±6.7 |
| **11** | InChI=1S/C18H28N2/c1-2-3-4-7-13-19-18-14-9-5-6-11-16(14)20-17-12-8-10-15(17)18/h2-13H2,1H3,(H,19,20) | 4.30±0.32 | 0.262±0.008 | 5.6±0.6 | n.d. | n.d. |
| **12** | InChI=1S/C13H19NO/c1-2-3-4-7-10-14-11-12-8-5-6-9-13(12)15/h5-6,8-9,11,15H,2-4,7,10H2,1H3/b14-11- | n.а. | 10.6±0.1%^[d]^ | 1.9±0.1 | n.d. | n.d. |
| **13** | InChI=1S/C13H21NO/c1-2-3-4-7-10-14-11-12-8-5-6-9-13(12)15/h5-6,8-9,14-15H,2-4,7,10-11H2,1H3 | 14.0±0.6%^[d]^ | 26.9±0.5%^[d]^ | 14.8±0.5 | n.d. | n.d. |

^a^ All experiments were carried out in accordance with the standard protocols approved by IPAС RAS. Human erythrocyte AChE, equine serum BChE were purchased from Milamed (Perm, Russia). Porcine liver CES, substrates, and reference compounds were from Sigma-Aldrich (St. Louis, MO, USA). The activity of enzymes was measured spectrophotometrically, as described in detail in ^[1]^ using ATCh iodide, BTCh iodide, and 4-NPA as substrates for AChE, BChE, and CES, respectively. Experimental conditions: K,Na-phosphate buffer (100 mM), 25 °С, pH 7.5 for AChE and BChE and pH 8.0 for CES assay. Measurements were carried out on a FLUOStar Optima microplate reader (BMG Labtech, Ortenberg, Germany). Test compounds were dissolved in DMSO; final concentration of solvent in the incubation mixture was 2% (v/v). Initial assessment of inhibitory activity was carried out by determining the degree of enzyme inhibition at a compound concentration of 20 µM. For active compounds (inhibition ≥35%), IC_50_ values were determined. Values without units of measurement for AChE, BChE and CES inhibition correspond to % inhibition at 20 µM.

^b^ The ability of the test compounds to competitively displace propidium, a selective ligand of the PAS of AChE, was evaluated by the fluorescence method ^[2-3]^ as described in detail in ^[4]^. Propidium iodide, donepezil and Electric eel AChE (*Ee*AChE, type VI-S, lyophilized powder) were purchased from Sigma-Aldrich (Saint Louis, MO, USA). The 7 μM *Ee*AChE was incubated with the test compound at a concentration of 20 μM in 1 mM Tris-HCl buffer pH 8.0, 25 °C, for 15 min. After that, propidium iodide was added at final concentration of 8 μM. The samples were incubated for 15 min and the fluorescence spectrum (530 nm (excitation) and 600 nm (emission)) was taken. Donepezil and tacrine were used as reference compounds. The measurements were carried out in triplicate on a FLUOStar Optima microplate reader. The degree of propidium displacement (% displacement) from the PAS of *Ee*AChE was calculated by formula (1):

% Displacement = 100 – (IF_AChE+ Propidium + inhibitor_ / IF_AChE + Propidium_) × 100 (1),

where IF_AChE + Propidium_ is the fluorescence intensity of the propidium associated with AChE in the absence of the test compound (taken as 100%), and IF_AChE + Propidium + inhibitor_ is the fluorescence intensity of the propidium associated with AChE in the presence of the test compound.

^c^ The inhibitory effect of the test compounds toward Aβ_42_ self-aggregation was determined using the thioflavin T (ThT) fluorescence method ^[5-7]^ with minor modifications as described in detail in ^[8]^. Lyophilized HFIP-pretreated Aβ_42_ from BACHEM (Bubendorf, Switzerland) was used. For the measurement of Aβ_42_ self-aggregation and assessment of inhibition of amyloid fibril formation by the tested compounds, aliquots of 500 μM Aβ_42_ stock solution in DMSO were diluted in 215 mM Na-phosphate buffer pH 8.0 to a final concentration of 50 μM Aβ_42_ and incubated for 24 h at 37 °C in the absence or presence of the tested compounds at a concentration of 100 µM. After that, the samples were incubated with 5 μM ThT in 50 mM glycine-NaOH buffer pH 8.5 for 10 min and the fluorescence was measured at 440 nm (excitation) and 485 nm (emission). Myricetin and propidium iodide were used as reference compounds (positive controls). Analyses were performed with a FLUOStar Optima microplate reader (LabTech, Ortenberg, Germany). The inhibition (%) of Aβ_42_ self-aggregation by the test compounds was calculated by the following equation:

% inhibition = 100 – (IF_i_ / IF_o_) ×100,

where IF_i_ and IF_o_ are the fluorescence intensities obtained for Aβ_42_ in the presence and in the absence of inhibitor, respectively, after subtracting the fluorescence of respective blanks.

^d^ % inhibition at 20 µM

n.d. – not determined. n.a. – not active

All values in the table are mean ± SEM (*n* > 3)

| **Compound No.** | **InChI** | **ABTS^•+^- scavenging activity^e^** | | **Fe^3+^-reducing activity (FRAP)^f^, TE** |
| --- | --- | --- | --- | --- |
|  |  | **TEAC** | **IC_50_, µM** |  |
| **7a** | InChI=1S/C23H29N3O2/c27-21-13-4-2-9-18(21)23(28)25-15-6-5-14-24-22-16-8-1-3-11-19(16)26-20-12-7-10-17(20)22/h2,4,9,13,27H,1,3,5-8,10-12,14-15H2,(H,24,26)(H,25,28) | 0.97±0.04 | 17.6±0.8 | n.a. |
| **7b** | InChI=1S/C25H33N3O2/c29-23-15-6-4-11-20(23)25(30)27-17-8-2-1-7-16-26-24-18-10-3-5-13-21(18)28-22-14-9-12-19(22)24/h4,6,11,15,29H,1-3,5,7-10,12-14,16-17H2,(H,26,28)(H,27,30) | 0.98±0.04 | 17.6±0.7 | n.a. |
| **7c** | InChI=1S/C27H37N3O2/c31-25-17-8-6-13-22(25)27(32)29-19-10-4-2-1-3-9-18-28-26-20-12-5-7-15-23(20)30-24-16-11-14-21(24)26/h6,8,13,17,31H,1-5,7,9-12,14-16,18-19H2,(H,28,30)(H,29,32) | 0.94±0.04 | 19.4±0.6 | n.a. |
| **9a** | InChI=1S/C23H29N3O/c27-22-13-4-1-8-17(22)16-24-14-5-6-15-25-23-18-9-2-3-11-20(18)26-21-12-7-10-19(21)23/h1,4,8,13,16,27H,2-3,5-7,9-12,14-15H2,(H,25,26)/b24-16+ | 0.65±0.03 | 26.6±1.1 | n.a. |
| **9b** | InChI=1S/C25H33N3O/c29-24-15-6-3-10-19(24)18-26-16-7-1-2-8-17-27-25-20-11-4-5-13-22(20)28-23-14-9-12-21(23)25/h3,6,10,15,18,29H,1-2,4-5,7-9,11-14,16-17H2,(H,27,28)/b26-18+ | 0.64±0.03 | 28.2±1.3 | n.a. |
| **9c** | InChI=1S/C27H37N3O/c31-26-17-8-5-12-21(26)20-28-18-9-3-1-2-4-10-19-29-27-22-13-6-7-15-24(22)30-25-16-11-14-23(25)27/h5,8,12,17,20,31H,1-4,6-7,9-11,13-16,18-19H2,(H,29,30)/b28-20+ | 0.63±0.02 | 29.6±1.2 | n.a. |
| **10a** | InChI=1S/C23H31N3O/c27-22-13-4-1-8-17(22)16-24-14-5-6-15-25-23-18-9-2-3-11-20(18)26-21-12-7-10-19(21)23/h1,4,8,13,24,27H,2-3,5-7,9-12,14-16H2,(H,25,26) | 1.5±0.08 | 10.1±0.5 | n.a. |
| **10b** | InChI=1S/C25H35N3O/c29-24-15-6-3-10-19(24)18-26-16-7-1-2-8-17-27-25-20-11-4-5-13-22(20)28-23-14-9-12-21(23)25/h3,6,10,15,26,29H,1-2,4-5,7-9,11-14,16-18H2,(H,27,28) | 1.48±0.07 | 9.5±0.6 | n.a. |
| **10c** | InChI=1S/C27H39N3O/c31-26-17-8-5-12-21(26)20-28-18-9-3-1-2-4-10-19-29-27-22-13-6-7-15-24(22)30-25-16-11-14-23(25)27/h5,8,12,17,28,31H,1-4,6-7,9-11,13-16,18-20H2,(H,29,30) | 1.48±0.05 | 10.1±0.6 | n.a. |
| **11** | InChI=1S/C18H28N2/c1-2-3-4-7-13-19-18-14-9-5-6-11-16(14)20-17-12-8-10-15(17)18/h2-13H2,1H3,(H,19,20) | n.a. | n.d. | n.a. |
| **12** | InChI=1S/C13H19NO/c1-2-3-4-7-10-14-11-12-8-5-6-9-13(12)15/h5-6,8-9,11,15H,2-4,7,10H2,1H3/b14-11- | 0.15±0.008 | 93.3±4.1 | n.a. |
| **13** | InChI=1S/C13H21NO/c1-2-3-4-7-10-14-11-12-8-5-6-9-13(12)15/h5-6,8-9,14-15H,2-4,7,10-11H2,1H3 | 1.52±0.07 | 10.7+0.4 | n.a. |

^e^ Radical scavenging activity of the compounds was evaluated using the ABTS radical cation (2,2ʹ-azinobis-(3-ethylbenzothiazoline-6-sulfonic acid, ABTS^•+^) decolorization assay ^[9]^ with minor modifications described in detail in ^[10]^. The reduction in absorbance was measured spectrophotometrically at 734 nm using a xMark UV/VIS microplate spectrophotometer (Bio-Rad, Hercules, CA, USA) for 1 h compared to a standard synthetic antioxidant Trolox (6-hydroxy-2,5,7,8-tetramethychroman-2-carboxylic acid). The antioxidant activity of the compounds was reported as Trolox equivalent antioxidant capacity (TEAC values) as the ratio of the slopes of the concentration−response curves, test compound/Trolox. The IC_50_ values for active compounds (compound concentration required for 50% reduction of the ABTS radical), were also determined.

^f^ The ferric reducing antioxidant power (FRAP) assay proposed by Benzie and Strain ^[11]^ modified to be performed in 96-well microplates as described in detail in ^[4]^ was used. 10 µL (0.5 mM) of the tested compound or reference compound were mixed with 240 µL of FRAP reagent and the absorbance of mixture was measured spectrophotometrically (λ = 593 nm) with a FLUOStar OPTIMA microplate reader at 600 nm after a 1 h incubation at 37 ⁰C against a blank. Trolox was used as a reference compound. The results were expressed as Trolox equivalents (TE) – the ratio of the concentrations of Trolox and the test compound resulting in the same effect on ferric reducing activity.

n.d. – not determined. n.a. – not active

All values in the table are mean ± SEM (*n* > 3)

**REFERENCES**

[1] G. F. Makhaeva, N. V. Kovaleva, N. P. Boltneva, S. V. Lushchekina, E. V. Rudakova, T. S. Stupina, A. A. Terentiev, I. V. Serkov, A. N. Proshin, E. V. Radchenko, V. A. Palyulin, S. O. Bachurin, R. J. Richardson, *Bioorg. Chem.* **2020**, *94*, 103387, doi 10.1016/j.bioorg.2019.103387

[2] P. Taylor, S. Lappi, *Biochemistry* **1975**, *14*, 1989-1997, doi 10.1021/bi00680a029

[3] P. Taylor, J. Lwebuga-Mukasa, S. Lappi, J. Rademacher, *Mol. Pharmacol.* **1974**, *10*, 703-708

[4] G. F. Makhaeva, N. V. Kovaleva, E. V. Rudakova, N. P. Boltneva, S. V. Lushchekina, Faingold, II, D. A. Poletaeva, Y. V. Soldatova, R. A. Kotelnikova, I. V. Serkov, A. K. Ustinov, A. N. Proshin, E. V. Radchenko, V. A. Palyulin, R. J. Richardson, *Molecules* **2020**, *25*, 5891, doi 10.3390/molecules25245891

[5] M. Bartolini, C. Bertucci, V. Cavrini, V. Andrisano, *Biochem. Pharmacol.* **2003**, *65*, 407-416, doi 10.1016/s0006-2952(02)01514-9

[6] P. Muñoz-Ruiz, L. Rubio, E. García-Palomero, I. Dorronsoro, M. del Monte-Millán, R. Valenzuela, P. Usán, C. de Austria, M. Bartolini, V. Andrisano, A. Bidon-Chanal, M. Orozco, F. J. Luque, M. Medina, A. Martínez, *J. Med. Chem.* **2005**, *48*, 7223-7233, doi 10.1021/jm0503289

[7] H. LeVine, 3rd, *Meth. Enzymol.* **1999**, *309*, 274-284, doi 10.1016/s0076-6879(99)09020-5

[8] G. F. Makhaeva, N. V. Kovaleva, E. V. Rudakova, N. P. Boltneva, M. V. Grishchenko, S. V. Lushchekina, T. Y. Astakhova, O. G. Serebryakova, E. N. Timokhina, E. F. Zhilina, E. V. Shchegolkov, M. V. Ulitko, E. V. Radchenko, V. A. Palyulin, Y. V. Burgart, V. I. Saloutin, S. O. Bachurin, R. J. Richardson, *Int. J. Mol. Sci.* **2023**, *24*, 2285, doi 10.3390/ijms24032285

[9] R. Re, N. Pellegrini, A. Proteggente, A. Pannala, M. Yang, C. Rice-Evans, *Free Radic. Biol. Med.* **1999**, *26*, 1231-1237, doi 10.1016/s0891-5849(98)00315-3

[10] G. F. Makhaeva, N. A. Elkina, E. V. Shchegolkov, N. P. Boltneva, S. V. Lushchekina, O. G. Serebryakova, E. V. Rudakova, N. V. Kovaleva, E. V. Radchenko, V. A. Palyulin, Y. V. Burgart, V. I. Saloutin, S. O. Bachurin, R. J. Richardson, *Bioorg. Chem.* **2019**, *91*, 103097, doi 10.1016/j.bioorg.2019.103097

[11] I. F. Benzie, J. J. Strain, *Meth. Enzymol.* **1999**, *299*, 15-27, doi 10.1016/s0076-6879(99)99005-5
